# Transition-Metal-Free [3+2] Dehydration Cycloaddition of Donor-Acceptor Cyclopropanes With 2-Naphthols

**DOI:** 10.3389/fchem.2021.711257

**Published:** 2021-07-16

**Authors:** Hua Zhao, Peng Shen, Dongru Sun, Hongbin Zhai, Yufen Zhao

**Affiliations:** ^1^ Institute of Drug Discovery Technology, Ningbo University, Ningbo, China; ^2^ State Key Laboratory of Chemical Oncogenomics, School of Chemical Biology and Biotechnology, Shenzhen Graduate School of Peking University, Shenzhen, China

**Keywords:** donor−acceptor cyclopropane, 2-naphthol, brønsted acid, [3+2] cyclization, Naphthalene-fused cyclopentane

## Abstract

A Brønsted acid-catalyzed domino ring-opening cyclization transformation of donor-acceptor (D-A) cyclopropanes and 2-naphthols has been developed. This formal [3+2] cyclization reaction provided novel and efficient access to the naphthalene-fused cyclopentanes in the absence of any transition-metal catalysts or additives. This robust procedure was completed smoothly on a gram-scale to afford the corresponding product with comparable efficiency. Furthermore, the synthetic application of the prepared product has been demonstrated by its transformation into a variety of synthetically useful molecules.

## Introduction

**SCHEME 1:**
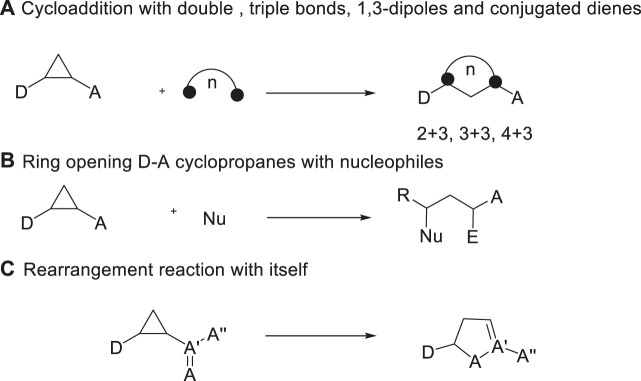
Different types of reactions of D-A cyclopropanes.

The demands for effective assembly of diverse molecular scaffolds are continuously growing along with the development of organic chemistry. Among various methods, domino ring-opening cyclization has recently emerged as a powerful tool for the rapid build-up of molecular complexity (Bhattacharyya et al., 2016; Lin et al., 2017; Sayyad et al., 2017; Yi et al., 2018; Wan and Liu, 2019). As a versatile class of three-atom building blocks, donor-acceptor (D-A) cyclopropanes have experienced an unexpected renaissance in the last 2 decades, which are widely exploited in methodology as well as natural product synthesis (Cavitt et al., 2014; Schneider et al., 2014; Grover et al., 2015; Novikov, 2015; Reiser, 2016; Ivanova and Trushkov, 2019; Werz and Biju, 2020). Due to their property of formation of 1,3-zwitterion intermediates with the help of the ring strain, D-A cyclopropanes could enter multitudinous kinds of chemical transformations with different counterparts in organic synthesis. Among the multiple reactions, Lewis acid-catalyzed (3 + n) ring-opening cyclization of D-A cyclopropanes represent the most convenient method to form the carbocycles and heterocycles, such as (3 + 2) cycloaddition with an unsaturated C-C multiple bond (Augustin et al., 2018; Ding et al., 2019; Huang et al., 2019; Mondal et al., 2019; Verma, et al., 2019; Xie et al., 2019), (3 + 3) cycloaddition with 1,3-dipoles (Dhote and Ramana, 2019; Petzold et al., 2019), and (3 + 4) cycloaddition with conjugated dienes (Ivanova et al., 2008; Garve et al., 2016; Wang et al., 2017; Zhang et al., 2017; Augustin et al., 2019a; Li et al., 2020) (Scheme 1A). In addition, the basic transformation of D-A cyclopropanes usually focuses on straightforward ring-opening reactions with nucleophiles, which allows ready access to 1,3-bifunctionalized derivatives (Garve et al., 2017; Lücht et al., 2017; Wallbaum et al., 2017; Das and DaniliucArmido, 2018; Augustin et al., 2019b; Lücht et al., 2019; Boichenko et al., 2020; Guin et al., 2020) (Scheme 1B). Moreover, the unexpected rearrangement of D-A cyclopropanes could lead to partially unsaturated five-membered heterocycles (Ivanova et al., 2018; Ortega, 2018; Shim et al., 2018) (Scheme 1C).

**SCHEME 2:**
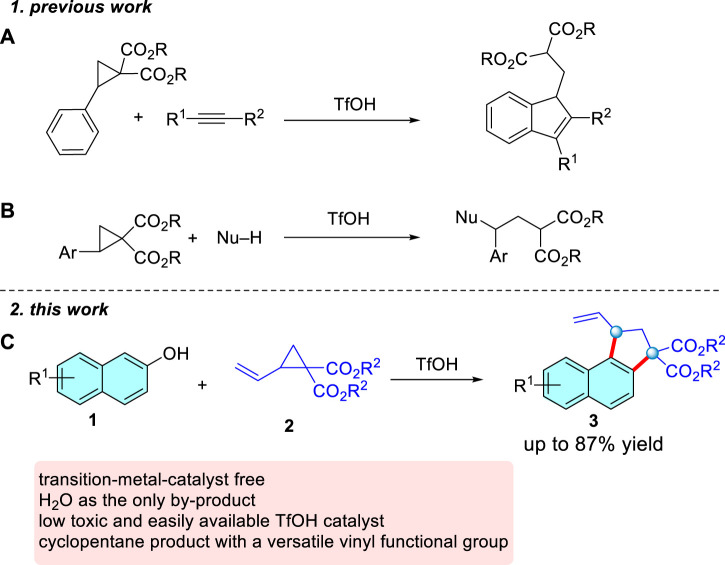
The Brønsted acid-catalyzed reactions of D-A cyclopropanes.

Typically, all the catalytic systems of D-A cyclopropanes employ high loadings of Lewis acidic catalysts, usually rare-earth triflates, with the reactions typically operating at elevated temperatures. Compared with those of Lewis acid-catalyzed reactions, the Brønsted acid-catalyzed conversion of donor-acceptor cyclopropanes has received only scant attention. In 2014, (3 + 2)-annulation of donor-acceptor cyclopropanes with alkynes induced by both Lewis and Brønsted acids was reported by Budynina (Rakhmankulov et al., 2015) (Scheme 2A). In 2018, Moran and co-workers presented an elegant nucleophilic ring opening of D-A cyclopropanes with nucleophiles in the presence of TfOH (Richmond et al., 2018) (Scheme 2B). Thus, developing sustainable alternative to achieve Brønsted acid-catalyzed reactions of donor-acceptor cyclopropanes is highly desirable. We notice that 2-naphthols commonly serve as important aromatic feedstocks in organic chemistry (Zhuo and You, 2013; Wang et al., 2015; Yang et al., 2015; Zheng et al., 2015; Cheng et al., 2016; Shen et al., 2017; Tu et al., 2017; Fang et al., 2018; Liu et al., 2018; Xia et al., 2019; Zhang et al., 2020), and Biju disclosed a formal (3 + 2) cyclopentannulation of 2-naphthols and D-A cyclopropanes catalyzed by Bi(OTf)_3_ and KPF_6_ (Kaicharla et al., 2016). But in the case of a reaction involving D-A cyclopropanes with vinyl as the only substrate, the cyclization product is obtained in an unsatisfactory yield (42%), which greatly inhibits the universality of the reaction. Given the versatility of the vinyl, here we report the successful realization of such a scenario, whereby TfOH acts as a highly active and general catalyst for the (3 + 2) dehydration annulation of D-A cyclopropanes and 2-naphthols (Scheme 2C). The salient features of this transformation include: (a) the use of nonmetallic, low-toxicity, and easily available TfOH as the catalyst, (b) simple and benign reaction conditions in the absence of additives, (c) a broad substrate scope with respect to 2-vinylcyclopropane-1,1-dicarboxylate in moderate to high yields, beyond the yields and scope disclosed in the previous work, and (d) the resulting product is easily transformed into synthetically useful compounds.

## Results and Discussion

**SCHEME 3:**
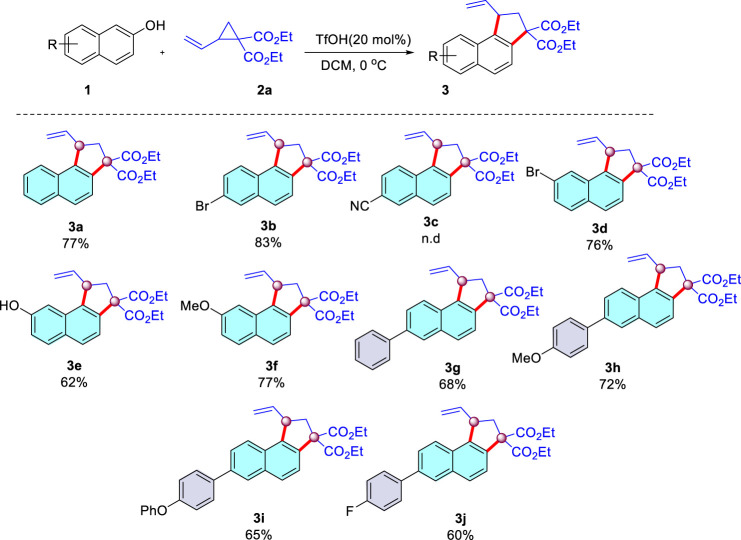
Scope of 2-naphthols.

We commenced our investigation with 2-naphthol Scheme 1A and diethyl 2-vinylcyclopropane-1,1-dicarboxylate Scheme 2A as model substrates. To our delight, treatment of Scheme 1A and Scheme 2A with 20 mol% of TfOH without other additives in toluene at 0°C furnished the (3 + 2) annulation product Scheme 3A in a 40% yield (Table 1, entry 1). Encouraged by the initial result, we then focused on solvent screening, and typical solvents including CH_3_CN, ^*i*^PrOH, DCE, hexane, and DCM were tested for the reaction (Table 1, entries 2–6). The results revealed that the solvents have great influence on the reaction outcome. Notably, DCM gave optimal results (77% yield, Table 1, entry 6) while others led to low yields of Scheme 3A. Next, the evaluation of a series of Brønsted acids were conducted, such as TsOH, MsOH, (±)-CSA, TFA, AcOH, HCl, H_2_SO_4_, and H_3_PO_4_. However, only under the catalysis of TsOH, MsOH, and TFA, the desired product was furnished at a 26–70% yield (Table 1, entries 7, 8, 10). Furthermore, efforts in running the reaction at room temperature proved to be unfruitful, as a slightly decreased yield (60%) of Scheme 3A was observed, and a complex reaction system was obtained when elevating the reaction temperature to 50°C (Table 1, entries 12–13).

**TABLE 1:** Optimization of reaction conditions*^a^*.

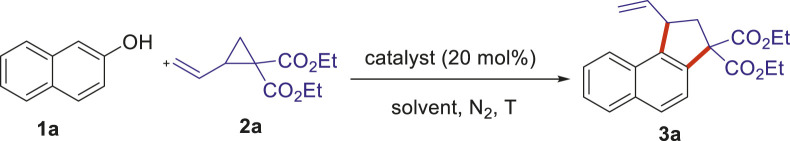

Entry	Catalyst	Solvent	Temp (°C)	Yield*^b^*
1	TfOH	Toluene	0	40
2	TfOH	CH_3_CN	0	44
3	TfOH	^*i*^PrOH	0	0
4	TfOH	DCE	0	64
5	TfOH	Hexane	0	50
**6**	**TfOH**	**DCM**	**0**	**77**
7	TsOH	DCM	0	40
8	MsOH	DCM	0	26
9	(±)-CSA*^c^*	DCM	0	0
10	TFA	DCM	0	70
11	AcOH	DCM	0	0
12	HCl	DCM	0	0
13	H_3_PO_4_	DCM	0	0
14	TfOH	DCM	Rt	60
15	TfOH	DCM	50	Mixture

a Reaction conditions: Scheme 1A (0.20 mmol), Scheme 2A (0.3 mmol), catalyst (20 mol%), solvent (1 ml), N_2_, 0°C, 12 h.

b Isolated yields.

c (±)-CSA = (±)-Camphorsulfonic acid.

With the optimized conditions determined, the generality of substrates with respect to 2-naphthols was then explored. As summarized in Scheme 3, an array of 2-naphthols underwent successful cyclization with diethyl 2-vinylcyclopropane-1,1-dicarboxylate Scheme 2A. First, 6-Br-2-naphthol was reacted with Scheme 2A, and the corresponding product Scheme 3B was obtained in an 83% yield. Whereas more electron-withdrawing cyano substituent decreased the performance of the reaction, providing almost no desirable product Scheme 3C. In addition, when the substrate with Br at the position of C7 of 2-naphthol was subjected to this reaction, it afforded Scheme 3D in a 76% yield. It is worth noting that when 2,7-dinaphthol bearing two reactive sites was chosen as the substrate, much to our surprise, monocyclic product Scheme 3E was isolated in a 62% yield. We speculated that a two-fold annulation product could be hampered by the unfavorable steric effect. Additionally, 2-naphthol with stronger electron-donating methoxy at the C7 position was also suitable for this reaction. Reaction of various 2-naphthol substrates bearing electron-donating or -withdrawing substituents at the phenyl residue provided the desired cyclization products in moderate to good yields (Schemes 3G–J, 60–72%). It is fascinating that the phenoxyphenyl substituent was also suitable to this condition, leading to a 65% yield of Scheme 3I. The structure of the Schemes 3A–J were characterized by ^1^H, ^13^C NMR, and HRMS (See Supplementary Material).

**SCHEME 4:**
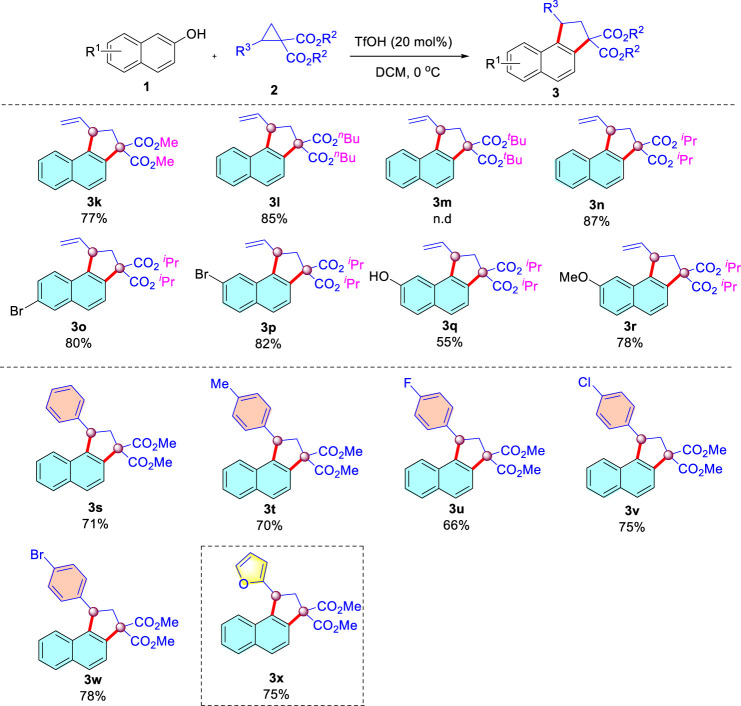
Scope of donor-acceptor cyclopropanes.

Next, we moved our attention to explore the scope of donor-acceptor cyclopropanes under the optimized conditions (Scheme 4). A series of 2-vinylcyclopropane-1,1-dicarboxylate (**2**, R = methyl, isopropyl, *n*-butyl) were compatible with the reaction conditions, leading to the corresponding dehydration annulation products in 77–87% yields. Unfortunately, D-A cyclopropane with tert-butyl shut down the desired transformation, presumably because the tert-butyl was readily hydrolyzed under strong acidic conditions. Similarly, when diisopropyl 2-vinylcyclopropane-1,1-dicarboxylate was reacted with substituted 2-naphthols, the desired products were isolated in 55–82% yields (Schemes 3O–3R). In addition, aromatic donors such as phenyl residues in this protocol were also successful, and an electron-donating substituent attached to the aromatic backbone worked in a moderate yield (Scheme 3T, 70% yield). Whereas more electron-withdrawing groups (F, Cl, Br) were also tolerated (Schemes 3U–W). Replacement of the benzene ring with a furan moiety in the substrate proved to be fine for the transformation (see Scheme 3X). The structure of the Schemes 3K–X were characterized by ^1^H, ^13^C NMR, and HRMS (See Supplementary Material).

**SCHEME 5:**
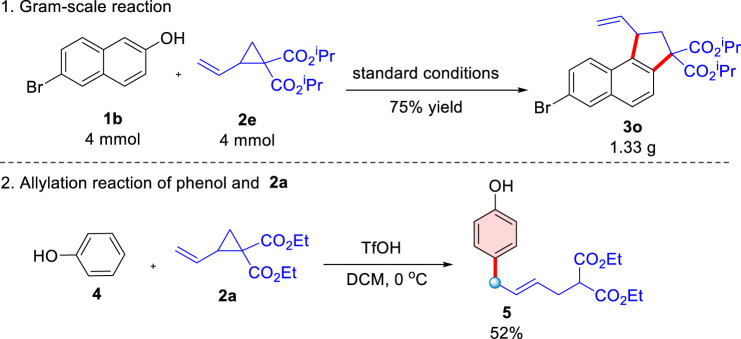
Gram-scale reaction and allylation reaction of phenol and 2-naphthol.

Encouraged by the high efficiency of the domino ring-opening cyclization reaction of donor-acceptor cyclopropanes with 2-naphthols, this TfOH-catalyzed reaction was completed smoothly on a gram-scale to afford the corresponding naphthalene-fused cyclopentane Scheme 3O with comparable efficiency (75% yield, Scheme 5). Interestingly, an extraordinary ring-opening reaction initiated at the end of the double bond of D-A cyclopropane Scheme 2A could be accessed when phenol was used as the substrate, uncyclized product Scheme 5 was afforded in a 52% yield, which suggested that ring-opening occurred *via* an S_N_2′-like mechanistic pathway. The structure of the Scheme 5 was characterized in the Supplementary Material.

**SCHEME 6:**
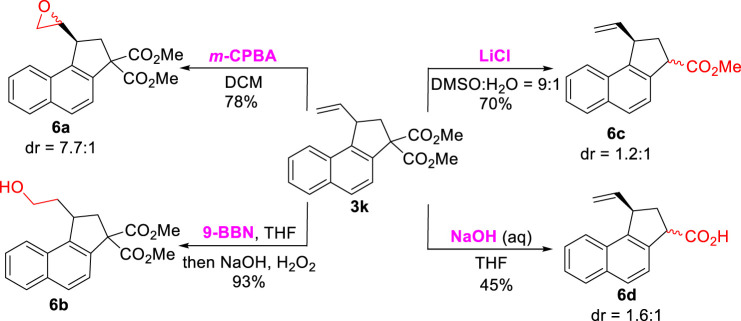
Transformation of **3k**.

To illustrate the application of this protocol, the transformation reactions with respect to product Scheme 3K were investigated (Scheme 6). First, efforts were focused on the versatile vinyl functional group, and the epoxidation of Scheme 3K with *m*-CPBA gave Scheme 6A in a 78% yield. In the presence of 9-BBN, Scheme 3K underwent hydroboration-oxidation to deliver primary alcohol Scheme 6B (93% yield). Furthermore, the treatment of Scheme 3K with LiCl in DMSO and H_2_O (9:1) furnished the selective decarboxylic product Scheme 6C in a 70% yield. Finally, the hydrolysis/decarboxylation reaction of Scheme 3K under an alkaline condition led to monocarboxyl product Scheme 6D in a 45% yield. The structure of the Schemes 6A–D were characterized by ^1^H, ^13^C NMR, and HRMS (See Supplementary Material).

**SCHEME 7:**
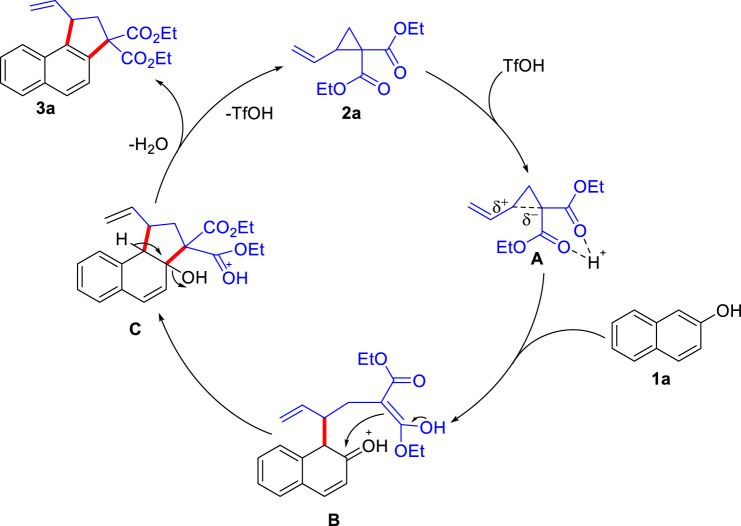
The proposed reaction mechanism.

Based on the previous report, we proposed a plausible mechanism of this Brønsted acid-catalyzed reaction (Scheme 7). Initial protonation of the “acceptor-motif” of cyclopropane Scheme 2A by TfOH possibly generates the intermediate **A**, in which the polarization of C−C bond increases. Ring-opening reaction of Scheme 1A to **A** generates the intermediate **B**. The subsequent intermolecular aldol reaction generates the cyclopentane intermediate **C**, which eliminates a molecule of water, and then forms the final product Scheme 3A, along with the regeneration of the TfOH catalyst which enters the next catalytic cycle.

## Conclusion

In summary, we have developed a robust strategy involving a Brønsted acid-facilitated domino ring-opening cyclization reaction, which provides efficient access to ubiquitous cyclopenta (a)naphthalene in moderate to good yields with high regioselectivity. Most importantly, this transformation avoids the use of metal-catalysts and external additives. Notably, a useful gram-scale reaction was completed smoothly *via* this protocol. Further applications involving Brønsted acid as a catalyst are under investigation in our laboratory and will be reported in due course.

## Data Availability

The original contributions presented in the study are included in the article/Supplementary Material, further inquiries can be directed to the corresponding authors.
