# Supplementary material for: Transition-Metal-Free [3+2] Dehydration Cycloaddition of Donor-Acceptor Cyclopropanes With 2-Naphthols
Source: Front Chem. 2021 Jul 16;9:711257. doi: 10.3389/fchem.2021.711257 (PMC8322234; doi:10.3389/fchem.2021.711257)
Supplement: Supplementary file 1 [file DataSheet1.docx]

Supplementary Material

# Supplementary Data

**1.1 Materials and methods**

All reactions were carried out under Argon atmosphere with dry solvents under anhydrous conditions, unless otherwise noted. All the chemicals were purchased commercially, and used without further purification. Anhydrous THF was distilled from sodium-benzophenone. Dichloromethane and was distilled from calcium hydride. Thin-layer chromatography (TLC) was conducted with 0.25 mm Tsingdao silica gel plates (60F-254) and visualized by exposure to UV light (254 nm) or stained with potassium permanganate. Flash column chromatography was performed on Tsingdao silica gel (200-300 mesh) and neutral/basic aluminum oxide (200-300mesh). ^1^H NMR Spectrum were recorded on Bruker spectrometers (at 300, 400 or 500 MHz) and reported relative to deuterated solvent signals or tetramethylsilane internal standard signals. Data for ^1^H NMR Spectrum were reported as follows: chemical shift (δ/ppm), multiplicity (s = singlet, d = doublet, t = triplet, q = quartet, m = multiplet, br = broad.), coupling constant (*J*/Hz) and integration. ^13^C NMR Spectrum were recorded on Bruker Spectrometers (100 or 125 MHz). Data for ^13^C NMR Spectrum were reported in terms of chemical shift. ^19^F NMR Spectrum were recorded on Bruker Spectrometers (376 MHz). High-resolution mass spectrometry (HRMS) was conducted on Bruker Apex IV RTMS.

## General procedure for Brønsted acid-catalyzed [3+2] Dehydration Cycloaddition of Donor−Acceptor Cyclopropanes with 2-Naphthols

A mixture of 2-substituted cyclopropane 1,1-dicarboxylates (0.3 mmol) with 2-naphthols (0.2 mmol) was added to a 25 mL sealed tube at 0 °C, and then TfOH (20 mol%) was added in the reaction system. The mixture was stirred at 0 °C for 12 h. After warming to room temperature, the reaction mixture was diluted with 5.0 mL of ethyl acetate and filtered through a plug of celite, followed by washing with 70 mL of ethyl acetate. The combined residue was concentrated under reduced pressure, and then the resulting crude product was purified by column chromatography on to provide the product.

**Diethyl 1-vinyl-1,2-dihydro-3H-cyclopenta[a]naphthalene-3,3-dicarboxylate (3a):** Eluent = petroleum ether/EtOAc (15:1). 52 mg, 77% yield. **^1^H NMR** (500 MHz, CDCl_3_) δ 8.05 – 8.01 (m, 1H), 7.90 – 7.86 (m, 1H), 7.81 (d, *J* = 8.5 Hz, 1H), 7.69 (d, *J* = 8.5 Hz, 1H), 7.49 – 7.47 (m, 2H), 6.01 (ddd, *J* = 18.5, 10.0, 8.5 Hz, 1H), 5.24 (ddd, *J* = 18.5, 2.0, 1.0 Hz, 1H), 5.13 (ddd, *J* = 10.0, 2.0, 1.0 Hz, 1H), 4.44 (td, *J* = 8.5, 4.5 Hz, 1H), 4.28 – 4.22 (m, 4H), 3.19 (dd, *J* = 13.5, 8.5 Hz, 1H), 2.72 (dd, *J* = 13.5, 4.0 Hz, 1H), 1.30 – 1.27 (m, 6H). **^13^C NMR** (125 MHz, CDCl_3_) δ 170.9, 170.8, 141.2, 141.2, 136.5, 134.0, 130.3, 128.5, 128.5, 126.0, 125.9, 124.8, 124.1, 115.3, 65.7, 61.8, 61.75, 47.4, 40.9, 14.1, 14.0. **HRMS** (ESI-TOF): *m/z* calcd. for C_21_H_23_O_4_: 339.1596 [M+H^+^]; found: 339.1592.

**Diethyl 7-bromo-1-vinyl-1,2-dihydro-3H-cyclopenta[a]naphthalene-3,3-dicarboxylate (3b):** Eluent = petroleum ether/EtOAc (12:1). 69 mg, 83% yield. **^1^H NMR** (500 MHz, CDCl_3_) δ 8.02 (s, 1H), 7.89 (d, J = 8.5 Hz, 1H), 7.69 (s, 2H), 7.53 (dd, J = 9.0, 0.5 Hz, 1H), 5.99 – 5.92 (m, 1H), 5.20 (d, J = 17.0 Hz, 1H), 5.11 (d, J = 10.0 Hz, 1H), 4.39 (td, J = 8.5, 4.5 Hz, 1H), 4.23 – 4.21 (m, 4H), 3.19 – 3.14 (m, 1H), 2.70 – 2.66 (m, 1H), 1.27 (t, J = 7.0 Hz, 6H). **^13^C NMR** (125 MHz, CDCl_3_) δ 170.7, 170.5, 141.5, 140.9, 137.1, 135.3, 130.5, 129.4, 128.8, 127.38, 126.6, 125.3, 120.1, 115.7, 65.7, 62.0, 61.9, 47.4, 40.8, 14.1, 14.0. **HRMS** (ESI-TOF): *m/z* calcd. for C_21_H_22_BrO_4_: 417.0701 [M+H^+^]; found: 417.0693.

**Diethyl 8-bromo-1-vinyl-1,2-dihydro-3H-cyclopenta[a]naphthalene-3,3-dicarboxylate (3d):** Eluent = petroleum ether/EtOAc (12:1). 63 mg, 76% yield. **^1^H NMR** (500 MHz, CDCl_3_) δ 8.17 (d, *J* = 2.0 Hz, 1H), 7.75 (d, *J* = 8.5 Hz, 1H), 7.73 (d, *J* = 8.5 Hz, 1H), 7.68 (d, *J* = 8.5 Hz, 1H), 7.54 (dd, *J* = 8.5, 2.0 Hz, 1H), 5.96 (ddd, *J* = 17.0, 10.0, 8.5 Hz, 1H), 5.22 (dt, *J* = 17.0, 6.0 Hz, 1H), 5.15 (dd, *J* = 10.0, 2.0 Hz, 1H), 4.36 (td, *J* = 8.5, 4.5 Hz, 1H), 4.27 – 4.20 (m, 4H), 3.16 (dd, *J* = 14.0, 9.0 Hz, 1H), 2.68 (dd, *J* = 13.5, 4.5 Hz, 1H), 1.27 (t, *J* = 7.5 Hz, 6H). **^13^C NMR** (125 MHz, CDCl_3_) δ 170.7, 170.6, 140.7, 140.4, 137.8, 132.5, 131.4, 130.1, 129.4, 128.2, 127.2, 124.6, 120.3, 115.9, 65.6, 62.0, 61.9, 47.3, 40.8, 14.1, 14.0. **HRMS** (ESI-TOF): *m/z* calcd. for C_21_H_22_BrO_4_: 417.0701 [M+H^+^]; found: 417.0693.

**Diethyl 8-hydroxy-1-vinyl-1,2-dihydro-3H-cyclopenta[a]naphthalene-3,3-dicarboxylate (3e):** Eluent = petroleum ether/EtOAc (5:1). 44 mg, 62% yield. **^1^H NMR** (500 MHz, CDCl_3_) δ 7.71 (d, *J* = 9.0 Hz, 1H), 7.69 (d, *J* = 8.5 Hz, 1H), 7.50 (d, *J* = 8.5 Hz, 1H), 7.29 (d, *J* = 2.5 Hz, 1H), 7.04 (dd, *J* = 8.5, 2.5 Hz, 1H), 5.93 (ddd, *J* = 17.0, 10.0, 8.5 Hz, 1H), 5.70 (s, 1H), 5.19 – 5.15 (m, 1H), 5.07 (dd, *J* = 10.0, 1.5 Hz, 1H), 4.29 – 4.20 (m, 5H), 3.14 (dd, *J* = 13.5, 8.5 Hz, 1H), 2.65 (dd, *J* = 4.0, 4.5 Hz, 1H), 1.28 – 1.25 (m, 6H). **^13^C NMR** (125 MHz, CDCl_3_) δ 171.1, 171.0, 153.8, 141.1, 139.6, 137.1, 131.5, 130.3, 129.3, 128.1, 121.5, 117.8, 115.4, 107.0, 65.7, 61.9, 61.8, 47.4, 40.9, 14.1, 14.0. **HRMS** (ESI-TOF): *m/z* calcd. for C_21_H_23_O_5_: 355.1545 [M+H^+^]; found: 355.1540.

**Diethyl 8-methoxy-1-vinyl-1,2-dihydro-3H-cyclopenta[a]naphthalene-3,3-dicarboxylate (3f):** Eluent = petroleum ether/EtOAc (12:1). 57 mg, 77% yield. **^1^H NMR** (500 MHz, CDCl_3_) δ 7.77 (d, *J* = 8.5 Hz, 1H), 7.73 (d, *J* = 8.5 Hz, 1H), 7.55 (d, *J* = 8.5 Hz, 1H), 7.34 (d, *J* = 2.0 Hz, 1H), 7.14 (dd, *J* = 9.5, 1.5 Hz, 1H), 6.03 – 5.96 (m, 1H), 5.28 (d, *J* = 17.5 Hz, 1H), 5.15 (dd, *J* = 10.0, 1.5 Hz, 1H), 4.38 (td, *J* = 8.5, 4.5 Hz, 1H), 4.29 – 4.20 (m, 4H), 3.86 (s, 3H), 3.18 (dd, *J* = 14.0, 9.0 Hz, 1H), 2.68 (dd, *J* = 13.5, 4.5 Hz, 1H), 1.30 – 1.26 (m, 6H). **^13^C NMR** (125 MHz, CDCl_3_) δ 171.0, 170.8, 157.7, 141.4, 139.9, 137.1, 131.4, 129.9, 129.4, 127.9, 121.7, 118.4, 115.2, 103.5, 65.7, 61.8, 61.7, 55.2, 47.7, 40.8, 14.1, 14.1. **HRMS** (ESI-TOF): *m/z* calcd. for C_22_H_25_O_5_: 369.1702 [M+H^+^]; found: 369.1693.

**Diethyl 7-phenyl-1-vinyl-1,2-dihydro-3H-cyclopenta[a]naphthalene-3,3-dicarboxylate (3g):** Eluent = petroleum ether/EtOAc (15:1). 57 mg, 68% yield. **^1^H NMR** (500 MHz, CDCl_3_) δ 8.09 (d, *J* = 8.5 Hz, 1H), 8.06 (d, *J* = 2.0 Hz, 1H), 7.85 (d, *J* = 8.5 Hz, 1H), 7.73 (dd, *J* = 8.5, 2.0 Hz, 1H), 7.70 (d, *J* = 7.5 Hz, 3H), 7.47 (t, *J* = 7.5 Hz, 2H), 7.47 (tt, *J* = 7.5, 1.0 Hz, 1H), 6.01 (ddd, *J* = 17.0, 10.0, 8.5 Hz, 1H), 5.26 (dt, *J* = 17.0, 1.5 Hz, 1H), 5.14 (dd, *J* = 10.0, 1.5 Hz, 1H), 4.44 (td, *J* = 8.5, 4.0 Hz, 1H), 4.24 – 4.20 (m, 4H), 3.19 (dd, *J* = 13.5, 8.5 Hz, 1H), 2.71 (dd, *J* = 13.5, 4.0 Hz, 1H), 1.27 (t, *J* = 7.0 Hz, 6H). **^13^C NMR** (125 MHz, CDCl_3_) δ 170.9, 170.8, 141.2, 141.0, 138.7, 136.7, 134.4, 129.4, 128.9, 128.6, 127.4, 127.3, 126.4, 125.8, 125.4, 124.6, 115.5, 65.7, 61.9, 61.8, 47.5, 40.9, 14.2, 14.1. **HRMS** (ESI-TOF): *m/z* calcd. for C_27_H_27_O_4_: 415.1909 [M+H^+^]; found: 415.1898.

**Diethyl 7-(4-methoxyphenyl)-1-vinyl-1,2-dihydro-3H-cyclopenta[a]naphthalene-3,3-dicarboxylate (3h):**  Eluent = petroleum ether/EtOAc (15:1). 64mg, 72% yield. **^1^H NMR** (500 MHz, CDCl_3_) δ 8.06 (d, *J* = 8.5 Hz, 1H), 8.01 (d, *J* = 2.0 Hz, 1H), 7.70 (dd, *J* = 9.0, 2.0 Hz, 1H), 7.68 (d, *J* = 8.5 Hz, 1H), 7.64 (d, *J* = 8.5 Hz, 2H), 7.01 (d, *J* = 8.5 Hz, 2H), 6.01 (ddd, *J* = 18.5, 10.0, 8.5 Hz, 1H), 5.25 (ddd, *J* = 17.0, 1.5, 1.0 Hz, 1H), 5.13 (ddd, *J* = 10.0, 1.5, 0.5 Hz, 1H), 4.44 (td, *J* = 9.0, 4.5 Hz, 1H), 4.27 – 4.21 (m, 4H), 3.87 (s, 3H), 3.18 (dd, *J* = 13.5, 8.5 Hz, 1H), 2.70 (dd, *J* = 13.5, 4.0 Hz, 1H), 1.28 (t, *J* = 7.0 Hz, 3H), 1.27 (t, *J* = 7.5 Hz, 3H). **^13^C NMR** (125 MHz, CDCl_3_) δ 171.0, 170.8, 159.3, 141.3, 141.2, 138.3, 136.4, 134.4, 133.5, 129.1, 128.5, 128.4, 125.6, 125.5, 125.3, 124.5, 115.4, 114.4, 65.7, 61.8, 61.7, 55.4, 47.4, 40.9, 14.1, 14.0. **HRMS** (ESI-TOF): *m/z* calcd. for C_28_H_29_O_5_: 445.2015 [M+H^+^]; found: 445.2008.

**Diethyl 7-(4-phenoxyphenyl)-1-vinyl-1,2-dihydro-3H-cyclopenta[a]naphthalene-3,3-dicarboxylate (3i):** Eluent = petroleum ether/EtOAc (15:1). 66 mg, 65% yield. **^1^H NMR** (500 MHz, CDCl_3_) δ 8.08 (d, *J* = 8.5 Hz, 1H), 8.03 (d, *J* = 2.0 Hz, 1H), 7.83 (d, *J* = 8.5 Hz, 1H), 7.72 – 7.65 (m, 4H), 7.38 – 7.35 (m, 2H), 7.15 – 7.07 (m, 5H), 6.01 (ddd, *J* = 17.0, 10.5, 8.5 Hz, 1H), 5.25 (td, *J* = 8.5, 1.5 Hz, 1H), 5.14 (dd, *J* = 10.0, 1.5 Hz, 1H), 4.44 (td, *J* = 8.5, 4.0 Hz, 1H), 4.27 – 4.21 (m, 4H), 3.19 (dd, *J* = 13.5, 8.5 Hz, 1H), 2.70 (dd, *J* = 14.0, 4.5 Hz, 1H), 1.28 (t, *J* = 7.0 Hz, 6H). **^13^C NMR** (125 MHz, CDCl_3_) δ 170.9, 170.7, 157.1, 157.0, 141.2, 138.0, 136.6, 136.0, 134.4, 129.8, 129.3, 128.7, 128.5, 126.0, 125.6, 125.4, 124.6, 123.5, 119.2, 119.1, 115.5, 65.7, 61.9, 61.8, 47.5, 40.9, 14.1, 14.0. **HRMS** (ESI-TOF): *m/z* calcd. for C_33_H_31_O_5_: 507.2171 [M+H^+^]; found: 507.2160.

**Diethyl 7-(4-fluorophenyl)-1-vinyl-1,2-dihydro-3H-cyclopenta[a]naphthalene-3,3-dicarboxylate (3j):** Eluent = petroleum ether/EtOAc (15:1). 52 mg, 60% yield. **^1^H NMR** (500 MHz, CDCl_3_) δ 8.08 (d, *J* = 8.5 Hz, 1H), 8.00 (d, *J* = 2.0 Hz, 1H), 7.83 (d, *J* = 8.5 Hz, 1H), 7.70 (d, *J* = 8.5 Hz, 1H), 7.67 (dd, *J* = 9.0, 2.0 Hz, 1H), 7.66 – 7.62 (m, 2H), 7.17 – 7.13 (m, 2H), 6.01 (ddd, *J* = 17.0, 10.0, 8.5 Hz, 1H), 5.25 (dt, *J* = 17.0, 1.0 Hz, 1H), 5.13 (dd, *J* = 10.0, 1.5 Hz, 1H), 4.44 (td, *J* = 9.0, 4.5 Hz, 1H), 4.27 – 4.21 (m, 4H), 3.19 (dd, *J* = 13.5, 8.5 Hz, 1H), 2.71 (dd, *J* = 13.5, 4.0 Hz, 1H), 1.28 (t, *J* = 7.0 Hz, 3H), 1.27 (t, *J* = 7.0 Hz, 3H). **^13^C NMR** (125 MHz, CDCl_3_) δ 170.9, 170.7, 162.6 (d, *J* = 245 Hz), 141.2, 141.1, 137.7, 137.1 (d, *J* = 2.5 Hz), 136.8, 134.3, 129.3, 128.9 (d, *J* = 7.5 Hz), 128.5, 126.2, 125.6, 125.5, 124. 7, 115.7 (d, *J* = 21.3 Hz), 115.5, 65.7, 61.9, 61.8, 47.4, 40.9, 14.1, 14.0. **HRMS** (ESI-TOF): *m/z* calcd. for C_27_H_26_FO_4_: 433.1815 [M+H^+^]; found: 433.1806.

**Dimethyl 1-vinyl-1,2-dihydro-3H-cyclopenta[a]naphthalene-3,3-dicarboxylate (3k)：**Eluent = petroleum ether/EtOAc (20:1). 48 mg, 77% yield. **^1^H NMR** (500 MHz, CDCl_3_) δ 8.03 – 8.01 (m, 1H), 7.87 – 7.85 (m, 1H), 7.79 (d, *J* = 8.5 Hz, 1H), 7.65 (d, *J* = 8.5 Hz, 1H), 7.49 – 7.45 (m, 2H), 5.98 (ddd, *J* = 17.0, 10.0, 8.5 Hz, 1H), 5.21 (ddd, *J* = 17.0, 2.0, 1.0 Hz, 1H), 5.12 (ddd, *J* = 10.0, 2.0, 1.0 Hz, 1H), 4.42 (td, *J* = 8.5, 4.0 Hz, 1H), 3.77 (s, 3H), 3.76 (s, 3H), 3.18 (dd, *J* = 13.5, 8.5 Hz, 1H), 2.71 (dd, *J* = 13.8, 4.3 Hz, 1H). **^13^C NMR** (125 MHz, CDCl_3_) δ 171.4, 171.3, 141.3, 141.0, 136.4, 134.1, 130.3, 128.5, 128.4, 126.2, 126.1, 124.8, 124.0, 115.5, 65.6, 53.0, 52.9, 47.4, 41.1. HRMS (ESI-TOF): *m/z* calcd. for C_19_H_19_O_4_: 311.1283 [M+H^+^]; found: 311.1274.

**Dibutyl 1-vinyl-1,2-dihydro-3H-cyclopenta[a]naphthalene-3,3-dicarboxylate (3l):** Eluent = petroleum ether/EtOAc (20:1). 67 mg, 85% yield. **^1^H NMR** (500 MHz, CDCl_3_) δ 8.02 – 8.01 (m, 1H), 7.87 – 7.85 (m, 1H), 7.79 (d, *J* = 8.5 Hz, 1H), 7.68 – 7.66 (m, 1H), 7.47 – 7.45 (m, 2H), 6.02 – 5.95 (m, 1H), 5.21 (dd, *J* = 17.0, 1.5 Hz, 1H), 5.11 (dd, *J* = 10.0, 1.5 Hz, 1H), 4.42 (td, *J* = 8.5, 4.5 Hz, 1H), 4.19 – 4.13 (m, 4H), 3.17 (dd, *J* = 13.5, 9.0 Hz, 1H), 2.70 (dd, *J* = 13.5, 4.3 Hz, 1H), 1.63 – 1.59 (m, 4H), 1,39 – 1.34 (m, 4H), 0.91 (t, *J* = 7.5 Hz, 6H). **^13^C NMR** (125 MHz, CDCl_3_) δ 171.0, 170.9, 141.2, 136.6, 134.1, 130.3, 128.5, 128.2, 126.1, 126.0, 124.9, 124.2, 115.3, 65.8, 65.7, 65.6, 47.5, 41.0, 30.6, 30.5, 19.1, 13.7. **HRMS** (ESI-TOF): *m/z* calcd. for C_25_H_13_O_4_: 395.2222 [M+H^+^]; found: 395.2214.

**Diisopropyl 1-vinyl-1,2-dihydro-3H-cyclopenta[a]naphthalene-3,3-dicarboxylate (3n)：**Eluent = petroleum ether/EtOAc (15:1). 64 mg, 87% yield. **^1^H NMR** (500 MHz, CDCl_3_) δ 8.03 – 7.99 (m, 1H), 7.87 – 7.85 (m, 1H), 7.78 (d, *J* = 8.5 Hz, 1H), 7.67 (d, *J* = 8.5 Hz, 1H), 7.48 – 7.44 (m, 2H), 5.99 (ddd, *J* = 17.0, 10.0, 8.5 Hz, 1H), 5.21 (ddd, *J* = 17.0, 1.5, 1.0 Hz, 1H), 5.10 (dd, *J* = 10.0, 1.5 Hz, 1H), 5.10 – 5.04 (m, 2H), 4.41 (td, *J* = 9.0, 4.0 Hz, 1H), 3.15 (dd, *J* = 14.0, 9.0 Hz, 1H), 2.66 (dd, *J* = 13.5, 4.0 Hz, 1H), 1.29 – 1.26 (m, 6H), 1.23 (t, *J* = 6.5 Hz, 6H). **^13^C NMR** (125 MHz, CDCl_3_) δ 170.4, 170.3, 141.4, 141.2, 136.7, 134.0, 130.3, 128.5, 128.1, 126.0, 125.9, 124.8, 124.12, 115.2, 69.3, 69.2, 65.8, 47.4, 40.7, 21.6, 21.5. **HRMS** (ESI-TOF): *m/z* calcd. for C_23_H_27_O_4_: 367.1909 [M+H^+^]; found: 367.1902.

**Diisopropyl 7-bromo-1-vinyl-1,2-dihydro-3H-cyclopenta[a]naphthalene-3,3-dicarboxylate (3o)：**Eluent = petroleum ether/EtOAc (15:1). 71 mg, 80% yield. **^1^H NMR** (500 MHz, CDCl_3_) δ 8.02 (d, *J* = 2.0 Hz, 1H), 7.89 (d, *J* = 9.0 Hz, 1H), 7.69 (s, 2H), 7.53 (dd, *J* = 8.5, 2.0 Hz, 1H), 5.95 (ddd, *J* = 17.0, 10.0, 8.5 Hz, 1H), 5.19 (dt, *J* = 17.0, 1.0 Hz, 1H), 5.11 (dd, *J* = 10.0, 1.5 Hz, 1H), 5.09 – 5.04 (m, 2H), 4.38 (td, *J* = 8.5, 4.0 Hz, 1H), 3.14 (dd, *J* = 13.5, 8.5 Hz, 1H), 2.64 (dd, *J* = 14.0, 4.5 Hz, 1H), 1.28 (d, *J* = 1.5 Hz, 3H), 1.26 (d, *J* = 1.5 Hz, 3H), 1.24 (d, *J* = 4.5 Hz, 3H), 1.23 (d, *J* = 5.0 Hz, 3H). **^13^C NMR** (125 MHz, CDCl_3_) δ 170.2, 170.0, 141.5, 141.1, 137.3, 135.2, 130.5, 129.3, 128.8, 127.2, 126.6, 125.3, 120.0, 115.5, 69.5, 69.3, 65.7, 47.3, 40.6, 21.6, 21.5, 21.5. **HRMS** (ESI-TOF): *m/z* calcd. for C_23_H_26_BrO_4_: 445.1014 [M+H^+^]; found: 445.1010.

**Diisopropyl 8-bromo-1-vinyl-1,2-dihydro-3H-cyclopenta[a]naphthalene-3,3-dicarboxylate (3p):** Eluent = petroleum ether/EtOAc (15:1). 73 mg, 82% yield. **^1^H NMR** (500 MHz, CDCl_3_) δ 8.17 (d, *J* = 1.5 Hz, 1H), 7.75 (d, *J* = 9.0 Hz, 1H), 7.72 (d, *J* = 8.5 Hz, 1H), 7.68 (d, *J* = 8.5 Hz, 1H), 7.53 (dd, *J* = 8.5, 2.0 Hz, 1H), 5.96 (ddd, *J* = 17.0, 10.0, 8.5 Hz, 1H), 5.22 (dt, *J* = 17.0, 1.5 Hz, 1H), 5.14 (dd, *J* = 10.5, 1.5 Hz, 1H), 5.09 – 5.04 (m, 2H), 4.35 (td, *J* = 8.5, 4.0 Hz, 1H), 3.14 (dd, *J* = 14.0, 9.0 Hz, 1H), 2.65 (dd, *J* = 14.0, 4.5 Hz, 1H), 1.28 (d, *J* = 2.5 Hz, 3H), 1.26 (d, *J* = 2.5 Hz, 3H), 1.24 – 1.22 (m, 6H). **^13^C NMR** (125 MHz, CDCl_3_) δ 170.2, 170.0, 140.9, 140.4, 138.0, 132.4, 131.5, 130.1, 129.3, 128.0, 127.2, 124.6, 120.2, 115.8, 69.5, 69.3, 65.7, 47.2, 40.6, 21.6, 21.5, 21.5. **HRMS** (ESI-TOF): *m/z* calcd. for C_23_H_26_BrO_4_: 445.1014 [M+H^+^]; found: 445.1012.

**Diisopropyl 8-hydroxy-1-vinyl-1,2-dihydro-3H-cyclopenta[a]naphthalene-3,3-dicarboxylate (3q)：**Eluent = petroleum ether/EtOAc (8:1). 42 mg, 55% yield. **^1^H NMR** (500 MHz, CDCl_3_) δ 7.71 (d, *J* = 9.0 Hz, 1H), 7.68 (d, *J* = 8.5 Hz, 1H), 7.50 (d, *J* = 8.5 Hz, 1H), 7.28 (d, *J* = 2.5 Hz, 1H), 7.03 (dd, *J* = 8.5, 2.5 Hz, 1H), 5.94 (ddd, *J* = 17.0, 10.0, 8.5 Hz, 1H), 5.57 (s, 1H), 5.17 (dt, *J* = 16.5, 1.5 Hz, 1H), 5.11 – 5.03 (m, 3H), 4.27 (td, *J* = 8.5, 4.0 Hz, 1H), 3.12 (dd, *J* = 13.5, 8.5 Hz, 1H), 2.62 (dd, *J* = 13.5, 4.0 Hz, 1H), 1.27 (d, *J* = 6.5 Hz, 6H), 1.24 (d, *J* = 6.5 Hz, 3H), 1.23 (d, *J* = 6.5 Hz, 3H). **^13^C NMR** (125 MHz, CDCl_3_) δ 170.6, 170.5, 153.7, 141.3, 139.6, 137.2, 131.5, 130.3, 129.3, 128.0, 121.6, 117.7, 115.3, 107.0, 69.5, 69.3, 65.8, 47.4, 40.7, 21.6, 21.5, 21.5. **HRMS** (ESI-TOF): *m/z* calcd. for C_23_H_27_O_5_: 383.1858 [M+H^+^]; found: 383.1852.

**Diisopropyl 8-methoxy-1-vinyl-1,2-dihydro-3H-cyclopenta[a]naphthalene-3,3-dicarboxylate(3r)：**Eluent = petroleum ether/EtOAc (15:1). 62 mg, 78% yield. **^1^H NMR** (500 MHz, CDCl_3_) δ 7.76 (d, *J* = 9.0 Hz, 1H), 7.71 (d, *J* = 8.5 Hz, 1H), 7.53 (d, *J* = 9.0 Hz, 1H), 7.32 (d, *J* = 2.5 Hz, 1H), 7.13 (dd, *J* = 9.0, 2.5 Hz, 1H), 5.98 (ddd, *J* = 17.0, 10.0, 8.5 Hz, 1H), 5.26 (ddd, *J* = 17.0, 2.0, 1.0 Hz, 1H), 5.13 (dd, *J* = 10.0, 2.0 Hz, 1H), 5.10 – 5.03 (m, 2H), 4.35 (td, *J* = 8.5, 4.5 Hz, 1H), 3.86 (s, 3H), 3.14 (dd, *J* = 13.5, 9.0 Hz, 1H), 2.63 (dd, *J* = 13.5, 4.5 Hz, 1H), 1.27 (d, *J* = 3.0 Hz, 3H), 1.26 (d, *J* = 3.0 Hz, 3H), 1.23 (d, *J* = 6.5 Hz, 6H). **^13^C NMR** (125MHz, CDCl_3_) δ 170.5, 170.3, 157.6, 141.6, 139.9, 137.2, 131.4, 129.9, 129.4, 127.8, 121.8, 118.4, 115.0, 103.5, 69.3, 69.2, 65.8, 55.2, 47.6, 40.7, 21.6, 21.5. **HRMS** (ESI-TOF): *m/z* calcd. for C_24_H_29_O_5_: 397.2015 [M+H^+^]; found: 397.2027.

**Dimethyl 1-phenyl-1,2-dihydro-3H-cyclopenta[a]naphthalene-3,3-dicarboxylate(3s)：**Eluent = petroleum ether/EtOAc (8:1). 51 mg, 71% yield. **^1^H NMR** (500 MHz, CDCl_3_) δ 7.86 – 7.83 (m, 2H), 7.73 (d, *J* = 8.5 Hz, 1H), 7.39 – 7.37 (m, 2H), 7.26 – 7.22 (m, 3H), 7.21 – 7.17 (m, 1H), 7.11 – 7.09 (m, 2H), 4.98 (dd, *J* = 9.0, 6.5 Hz, 1H), 3.75 (s, 3H), 3.73 (s, 3H), 3.55 (dd, *J* = 13.5, 8.5 Hz, 1H), 2.67 (dd, *J* = 14.0, 6.5 Hz, 1H). **^13^C NMR** (125 MHz, CDCl_3_) δ 171.5, 171.1, 145.2, 141.9, 137.4, 134.4, 129.9, 128.8, 128.7, 128.5, 127.8, 126.6, 126.1, 126.0, 125.3, 123.9, 65.7, 53.1, 52.9, 49.3, 44.7. **HRMS** (ESI-TOF): *m/z* calcd. for C_23_H_21_O_4_: 361.1440 [M+H^+^]; found: 361.1431.

**Dimethyl 1-(p-tolyl)-1,2-dihydro-3H-cyclopenta[a]naphthalene-3,3-dicarboxylate (3t):** Eluent = petroleum ether/EtOAc (15:1). 52 mg, 70% yield. **^1^H NMR** (500 MHz, CDCl_3_) δ 7.84 (t, *J* = 8.0 Hz, 2H), 7.72 (dd, *J* = 9.0, 1.5 Hz, 1H), 7.41 (d, *J* = 8.0 Hz, 1H), 7.39 (d, *J* = 8.5 Hz, 1H), 7.25 – 7.23 (m, 1H), 7.06 (d, *J* = 7.0 Hz, 2H), 6.99 (d, *J* = 6.5 Hz, 2H), 4.95 (t, *J* = 7.5 Hz, 1H), 3.75 – 3.73 (m, 6H), 3.53 (ddd, *J* = 13.5, 8.5, 2.0 Hz, 1H), 2.64 (ddd, *J* = 14.0, 6.5, 2.0 Hz, 1H), 2.30 (s, 3H). **^13^C NMR** (125 MHz, CDCl_3_) δ 171.5, 171.2, 142.2, 142.0, 137.3, 136.1, 134.3, 130.0, 129.4, 128.6, 128.5, 127.6, 126.1, 126.0, 125.3, 123.9, 65.7, 53.1, 52.9, 48.9, 44.8, 21.1. **HRMS** (ESI-TOF): *m/z* calcd. for C_24_H_23_O_4_: 375.1596 [M+H^+^]; found: 375.1590.

**Dimethyl 1-(4-fluorophenyl)-1,2-dihydro-3H-cyclopenta[a]naphthalene-3,3-dicarboxylate (3u):** Eluent = petroleum ether/EtOAc (15:1). 50 mg, 66% yield. **^1^H NMR** (500 MHz, CDCl_3_) δ 7.85 (dd, *J* = 8.5, 3.0 Hz, 2H), 7.73 (d, *J* = 8.5 Hz, 1H), 7.40 (ddd, *J* = 8.0, 6.5, 1.5 Hz, 1H), 7.36 (d, *J* = 8.5 Hz, 1H), 7.25 (ddd, *J* = 8.5, 6.5, 1.5 Hz, 1H), 7.05 (dd, *J* = 8.5, 5.5 Hz, 2H), 6.92 (t, *J* = 8.6 Hz, 2H), 4.97 (dd, *J* = 8.8, 6.0 Hz, 1H), 3.75 (s, 3H), 3.73 (s, 3H), 3.53 (dd, *J* = 13.5, 8.5 Hz, 1H), 2.68 – 2.59 (m, 1H). **^13^C NMR** (125 MHz, CDCl_3_) δ 171.4, 171.1, 161.6 (d, *J* = 243.7 Hz), 141.5, 140.7 (d, *J* = 3.7 Hz), 137.4, 134.4, 129.8, 129.2 (d, *J* = 7.5 Hz), 128.9, 128.6, 126.2, 126.1, 125.1, 123.9, 115.6 (d, *J* = 21.3 Hz), 65.7, 53.1, 53.0, 48.5, 44.6. **^19^F NMR** (470 MHz, CDCl_3_) δ -116.34. **HRMS** (ESI-TOF): *m/z* calcd. for C_23_H_20_FO_4_: 379.1346 [M+H^+^]; found: 379.1337.

**Dimethyl 1-(4-chlorophenyl)-1,2-dihydro-3H-cyclopenta[a]naphthalene-3,3-dicarboxylate (3v):** Eluent = petroleum ether/EtOAc (15:1). 59 mg, 75% yield. **^1^H NMR** (500 MHz, CDCl_3_) δ 7.86 (t, *J* = 7.5 Hz, 2H), 7.72 (d, *J* = 8.5 Hz, 1H), 7.42 (ddd, *J* = 8.0, 7.0, 1.5 Hz, 1H), 7.35 (d, *J* = 8.5 Hz, 1H), 7.29 – 7.27 (m, 1H), 7.22 (d, *J* = 8.5 Hz, 2H), 7.03 (d, *J* = 8.0 Hz, 2H), 4.96 (dd, *J* = 9.0, 6.0 Hz, 1H), 3.76 (s, 3H), 3.74 (s, 3H), 3.53 (dd, *J* = 13.5, 8.5 Hz, 1H), 2.63 (dd, *J* = 13.5, 6.0 Hz, 1H). **^13^C NMR** (125 MHz, CDCl_3_) δ 171.4, 171.0, 143.6, 141.2, 137.5, 134.3, 132.3, 129.7, 129.1, 129.0, 128.9, 128.6, 126.3, 126.1, 125.1, 123.9, 65.7, 53.1, 53.0, 48.6, 44.4. **HRMS** (ESI-TOF): *m/z* calcd. for C_23_H_20_ClO_4_: 395.1050 [M+H^+^]; found: 395.1045.

**Dimethyl 1-(4-bromophenyl)-1,2-dihydro-3H-cyclopenta[a]naphthalene-3,3-dicarboxylate (3w):** Eluent = petroleum ether/EtOAc (15:1). 68 mg, 78% yield. **^1^H NMR** (500 MHz, CDCl_3_) δ 7.86 (d, *J* = 4.0 Hz, 1H), 7.85 (d, *J* = 4.0 Hz, 1H), 7.73 (dd, *J* = 8.5, 4.0 Hz, 1H), 7.44 – 7.39 (m, 1H), 7.38 – 7.34 (m, 3H), 7.29 – 7.24 (m, 1H), 6.98 – 6.96 (m, 2H), 4.97 – 4.93 (m, 1H), 3.76 (s, 3H), 3.73 (s, 3H), 3.54 (dd, *J* = 13.5, 4.0 Hz, 1H), 2.63 (dd, *J* = 13.5, 6.0 Hz, 1H). **^13^C NMR** (125 MHz, CDCl_3_) δ 171.4, 171.0, 144.2, 141.1, 137.5, 134.4, 131.9, 129.7, 129.5, 129.0, 128.7, 126.3, 126.2, 125.1, 123.9, 120.4, 65.7, 53.2, 53.0, 48.6, 44.4. **HRMS** (ESI-TOF): *m/z* calcd. for C_23_H_20_BrO_4_: 439.0545 [M+H^+^]; found: 439.0540.

**Dimethyl 1-(furan-2-yl)-1,2-dihydro-3H-cyclopenta[a]naphthalene-3,3-dicarboxylate (3x):** Eluent = petroleum ether/EtOAc (15:1). 53 mg, 75% yield. **^1^H NMR** (500 MHz, CDCl_3_) δ 7.87 (d, *J* = 8.0 Hz, 1H), 7.84 (d, *J* = 8.5 Hz, 1H), 7.69 (d, *J* = 8.5 Hz, 1H), 7.66 (d, *J* = 8.5 Hz, 1H), 7.45 (t, *J* = 7.0 Hz, 1H), 7.39 (t, *J* = 8.0 Hz, 1H), 7.30 (d, *J* = 1.5 Hz, 1H), 6.24 (dd, *J* = 3.0, 2.0 Hz, 1H), 5.87 (d, *J* = 3.0 Hz, 1H), 5.05 (dd, *J* = 8.5, 4.5 Hz, 1H), 3.78 (s, 3H), 3.71 (s, 3H), 3.35 (dd, *J* = 13.5, 9.0 Hz, 1H), 3.00 (dd, *J* = 13.5, 5.0 Hz, 1H). **^13^C NMR** (125 MHz, CDCl_3_) δ 171.1, 171.0, 156.4, 141.6, 139.6, 136.9, 134.1, 130.0, 128.8, 128.5, 126.4, 126.1, 124.5, 123.9, 110.3, 106.3, 65.6, 53.1, 52.9, 42.0, 41.0. **HRMS** (ESI-TOF): *m/z* calcd. for C_21_H_19_O_5_: 351.1232 [M+H^+^]; found:351.1228.

**Diethyl (E)-2-(4-(4-hydroxyphenyl)but-2-en-1-yl)malonate (5)**: Eluent = petroleum ether/EtOAc (10:1). 32 mg, 52% yield. **^1^H NMR** (500 MHz, CDCl_3_) δ 6.99 (d, *J* = 7.0 Hz, 2H), 6.74 (d, *J* = 7.0 Hz, 2H), 5.65 (dt, *J* = 16.0, 7.5 Hz, 1H), 5.44 (dt, *J* = 16.0, 7.5 Hz, 1H), 5.12 (s, 1H), 4.18 – 4.14 (m, 4H), 3.39 (t, *J* = 7.5 Hz, 1H), 3.23 (d, *J* = 6.5 Hz, 2H), 2.62 (d, *J* = 8.0 Hz, 2H), 1.27 – 1.23 (m, 6H). **^13^C NMR** (125 MHz, CDCl_3_) δ 169.1, 154.0, 132.8, 132.3, 129.5, 126.5, 115.2, 61.4, 52.2, 38.0, 31.7, 14.1. **HRMS** (ESI-TOF): *m/z* calcd. for C_17_H_23_O_5_: 307.1545 [M+H^+^]; found: 307.1541.

3k (124 mg, 0.4 mmol, 1.00 equiv.) was dissolved in CH2Cl2 (4.0 mL) followed by addition of m-CPBA (138 mg, 0.8 mmol, 2.00 equiv.) and stirred overnight. The solution was then diluted with CH2Cl2 (20.0 mL) und washed with NaOH solution (2.0 M, 3 × 10 mL) and with brine (10 mL). The organic phase was dried over Na2SO4 and the solvent was removed in vacuo, the residual was purified by column chromatography (SiO_2_, petroleum ether: ethyl acetate = 8:1) affording the compound **6a** in 78% yield (dr = 7.7:1, 102 mg).

**Dimethyl 1-(oxiran-2-yl)-1,2-dihydro-3H-cyclopenta[a]naphthalene-3,3-dicarboxylate (6a)：** **^1^H NMR** (500 MHz, CDCl_3_) δ 7.85 – 7.80 (m, 2H), 7.75 (d, *J* = 8.5 Hz, 1H), 7.60 (d, *J* = 8.5 Hz, 1H), 7.47 – 7.39 (m, 2H), 3.72 (s, 3H), 3.71 (s, 3H), 3.69 – 3.68 (m, 1H), 3.08 – 3.02 (m, 2H), 2.95 (dd, *J* = 14.0, 3.0 Hz, 1H), 2.79 (dd, *J* = 5.0, 3.5 Hz, 1H), 2.66 (dd, *J* = 5.0, 3.0 Hz, 1H). **^13^C NMR** (125 MHz, CDCl_3_) δ 170.4, 170.2, 138.2, 136.3, 133.0, 129.3, 127.8, 127.6, 125.5, 125.2, 123.8, 123.0, 64.6, 54.2, 52.1, 52.0, 47.4, 44.1, 36.3. **HRMS** (ESI-TOF): *m/z* calcd. for C_19_H_18_NaO_5_: 349.1052 [M+Na^+^]; found: 349.1042.

A solution of **3k** (62 mg, 0.2 mmol) in dry THF (2 mL) at room temperation was added dropwise 9-BBN (0.5 M in THF, 1.2 mL). The mixture was stirred at 25 ^o^C for 2h, and this mixture was added dropwise water (0.6 mL), aq NaOH (3 M, 3 mL), then 30% aq H_2_O_2_ (3 mL). After being stirred at 40 ^o^C for 2 h, the mixture was extracted three time with ether (3×15 mL). The organic layer was combined and dried (Na_2_SO_4_). After remove all of the solvents under reduced pressure, the residual was purified by column chromatography (SiO_2_, petroleum ether: ethyl acetate = 5:1 to 3:1) affording the compound **6b** in 93% yield (61 mg).

**Dimethyl 1-(2-hydroxyethyl)-1,2-dihydro-3H-cyclopenta[a]naphthalene-3,3-dicarboxylate (6b)：^1^H NMR** (500 MHz, CDCl_3_) δ 7.93 (dd, *J* = 7.5, 1.5 Hz, 1H), 7.87 (dd, *J* = 7.5, 2.0 Hz, 1H), 7.77 (d, *J* = 8.5 Hz, 1H), 7.62 (d, *J* = 8.5 Hz, 1H), 7.53 – 7.40 (m, 2H), 3.99 – 3.94 (m, 1H), 3.90 – 3.84 (m, 2H), 3.78 (s, 3H), 3.76 (s, 3H), 2.94 – 2.93 (m, 2H), 2.26 – 2.19 (m, 1H), 1.78 – 1.65 (m, 2H). **^13^C NMR** (125 MHz, CDCl_3_) δ 171.9, 171.8, 144.2, 135.3, 134.1, 129.4, 128.7, 128.0, 126.3, 126.1, 124.5, 124.2, 66.0, 61.6, 53.1, 53.0, 39.3, 38.6, 38.1. **HRMS** (ESI-TOF): *m/z* calcd. for C_19_H_21_NaO_5_: 351.1208 [M+Na^+^]; found: 351.1200.

A mixture of **3k** (62 mg, 0.2 mmol) in DMSO (2 mL) was heated for 140 ^o^C for 7 h. Then the mixture was cooled to rt, and then diluted with water, extracted with EtOAc three times. The combined organic layers were washed with brine and filtered. The filtrate was evaporated in vacuo. the residual was purified by column chromatography (SiO_2_, petroleum ether: ethyl acetate = 10:1) affording the compound **6c** in 70% yield (dr = 1.2:1, 35 mg).

**Methyl-1-vinyl-2,3-dihydro-1H-cyclopenta[a]naphthalene-3-carboxylate (6c):**

For major isomer: **^1^H NMR** (500 MHz, CDCl_3_) 7.88 – 7.85 (m, 1H), 7.77 (d, *J* = 3.5 Hz, 1H), 7.66 (d, *J* = 3.5 Hz, 1H), 7.39 – 7.35 (m, 3H), 5.93 – 5.85 (m, 1H), 5.03 – 4.98 (m, 2H), 4.37 (td, *J* = 8.0, 4.0 Hz, 1H), 4.22 – 4.21 (m, 1H), 3.69 (d, *J* = 3.5 Hz, 3H), 2.79 – 2.72 (m, 1H), 2.30 – 2.23 (m, 1H). **^13^C NMR** (125 MHz, CDCl_3_) δ 174.4, 142.1, 140.7, 137.8, 133.5, 130.41, 128.5, 128.3, 126.1, 125.5, 124.7, 122.7, 114.7, 52.2, 49.5, 47.4, 36.4. For minor isomer: **^1^H NMR** (500 MHz, CDCl_3_) δ 7.97 – 7.92 (m, 1H), 7.78 (d, *J* = 3.0 Hz, 1H), 7.68 (d, *J* = 3.0 Hz, 1H), 7.45 – 7.41 (m, 3H), 6.04 – 5.96 (m, 1H), 5.16 (dd, *J* = 17.0, 3.0 Hz, 1H), 4.97 (dd, *J* = 17.0, 3.5 Hz, 1H), 4.25 – 4.23 (m, 1H), 4.10 (td, *J* = 9.0, 4.5 Hz, 1H), 3.67 (d, *J* = 3.5 Hz, 3H), 2.71 – 2.65 (m, 1H), 2.41 – 2.36 (m, 1H). ^13^C NMR (125 MHz, CDCl_3_) δ 174.4, 140.6, 140.5, 137.8, 133.6, 130.6, 128.5, 128.3, 126.1, 125.5, 124.7, 123.2, 114.6, 52.2, 50.0, 48.6, 35.7. **HRMS** (ESI-TOF): *m/z* calcd. for C_17_H_16_NaO_2_: 275.1048 [M+ Na^+^]; found: 275.1038

A solution of **3k** (62 mg, 0.2 mmol) in THF (2 mL) was added aq. NaOH (3M, 3mL), the mixture heated for 40 ^o^C for 10 h. Then the mixture was cooled to rt, and then adjust the pH with with hydrochloric acid (2M), extracted with EtOAc three times. The combined organic layers were washed with brine and filtered. The filtrate was evaporated in vacuo. the residual was purified by column chromatography (SiO_2_, petroleum ether: ethyl acetate = 2:1) affording the compound **6d** in 45% yield (dr = 1.6:1, 22 mg).

**1-vinyl-2,3-dihydro-1H-cyclopenta[a]naphthalene-3-carboxylic acid (6d):** For major isomer: **^1^H NMR** (500 MHz, CDCl_3_) 7.94 (d, *J* = 8.0 Hz, 1H), 7.85 (d, *J* = 8.0 Hz, 1H), 7.76 (d, *J* = 8.5 Hz, 1H), 7.56 (d, *J* = 8.5 Hz, 1H), 7.51 – 7.46 (m, 2H), 5.97 (ddd, *J* = *J* = 17.0, 10.0, 7.5 Hz, 1H), 5.10 – 5.07 (m, 2H), 4.46 (td, *J* = 8.5, 3.5 Hz, 1H), 4.33 (t, *J* = 8.0 Hz, 1H), 2.85 – 2.80 (m, 1H), 2.36 (ddd, *J* = 13.0, 8.0, 3.5 Hz, 1H). **^13^C NMR** (125 MHz, CDCl_3_) δ 179.7, 140.8, 140.5, 137.1, 133.6, 130.4, 128.6, 128.3, 126.2, 125.6, 124.7, 122.7, 114.9, 49.3, 47.4, 36.3. For minor isomer: **^1^H NMR** (500 MHz, CDCl_3_) δ 8.02 (dd, *J* = 7.0, 2.5 Hz,1H), 7.84 (d, *J* = 8.0 Hz, 1H), 7.76 (d, *J* = 8.5 Hz, 1H), 7.56 (d, *J* = 8.5 Hz, 1H), 7.45 – 7.43 (m, 2H), 6.07 (ddd, *J* = 17.0, 10.0, 8.5 Hz, 1H), 5.26 – 5.22 (m, 1H), 5.07 – 5.05 (m, 1H), 4.34 – 4.30 (m, 1H), 4.20 (dd, *J* = 9.5, 4.5 Hz, 1H), 2.81 – 2.77 (m, 1H), 2.47 (dt, *J* = 13.5, 4.0 Hz, 1H). **^13^C NMR** (126 MHz, CDCl_3_) δ 179.7, 142.0, 140.7, 137.1, 133.7, 130.6, 128.6, 128.4, 126.1, 125.6, 124.7, 123.2, 114.7, 49.9, 48.6, 35.4. **HRMS** (ESI-TOF): *m/z* calcd. for C_16_H_15_O_2_: 239.1072 [M+H^+^]; found: 239.1065.

**2. ^1^H, ^13^C and ^19^F NMR Spectra of Compounds**


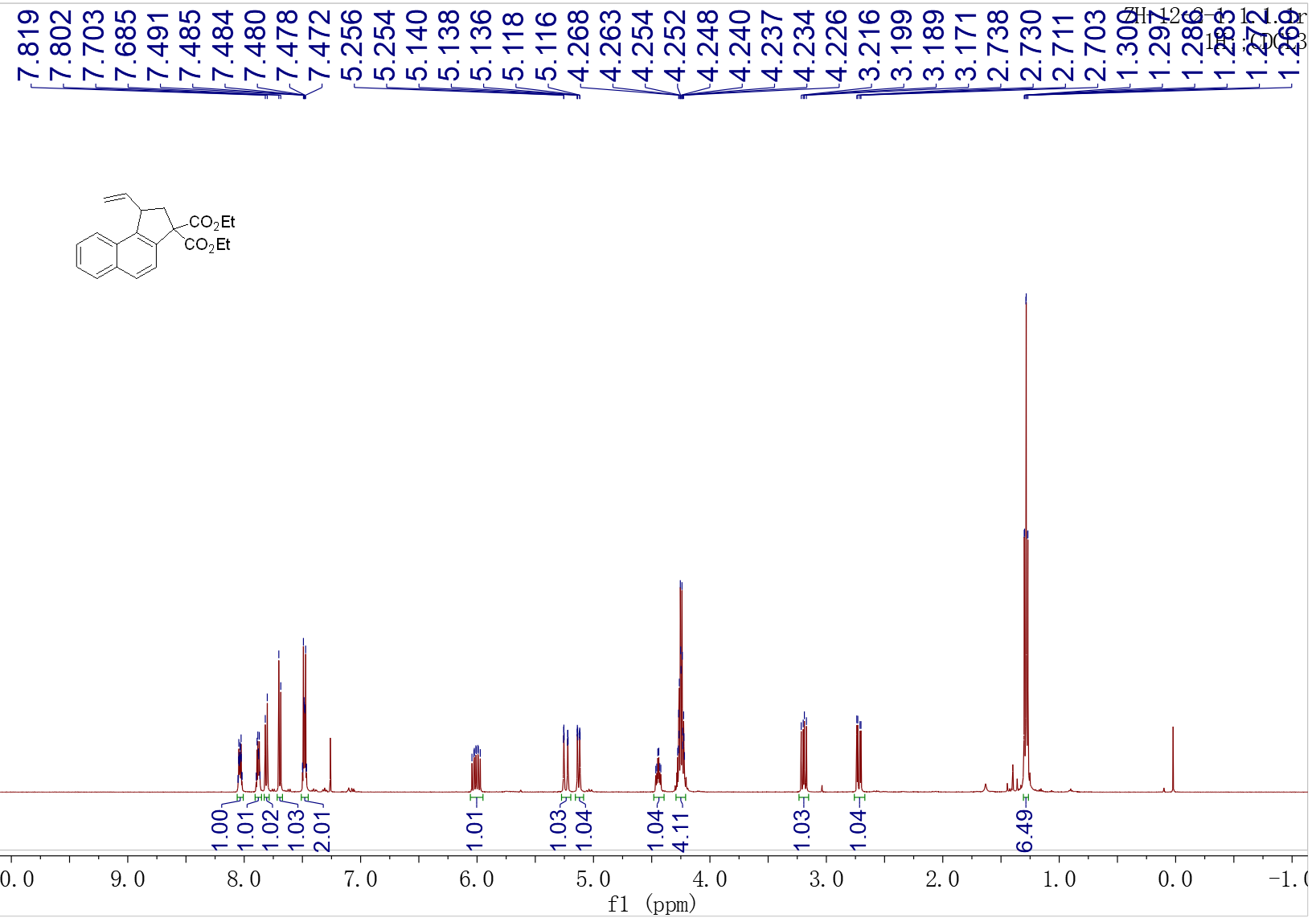


**Supplementary Figure 1. ^1^H NMR spectrum of 3a (500 MHz, CDCl_3_)**


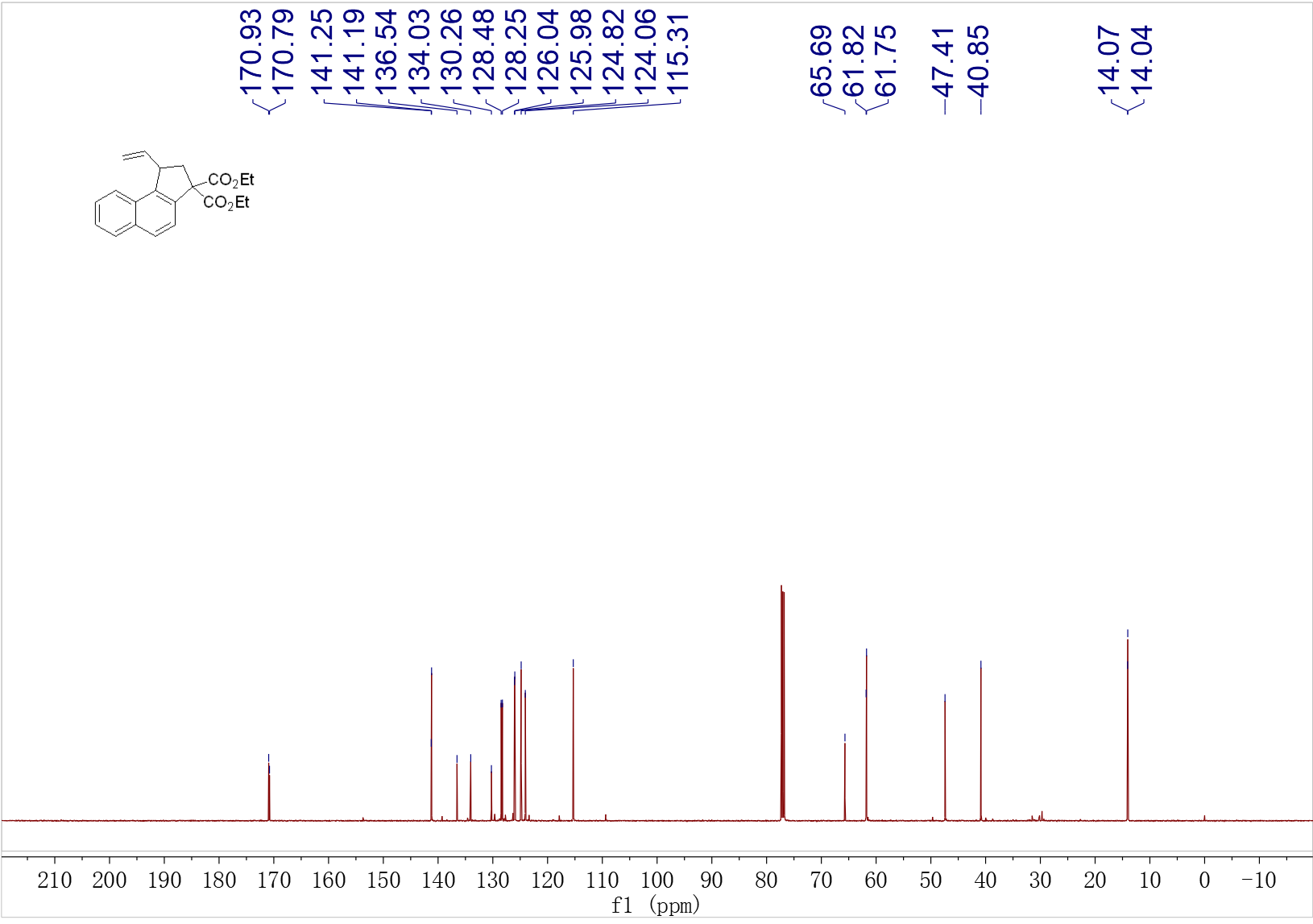


**Supplementary Figure 2. ^13^C NMR spectrum of 3a (125 MHz, CDCl_3_)**


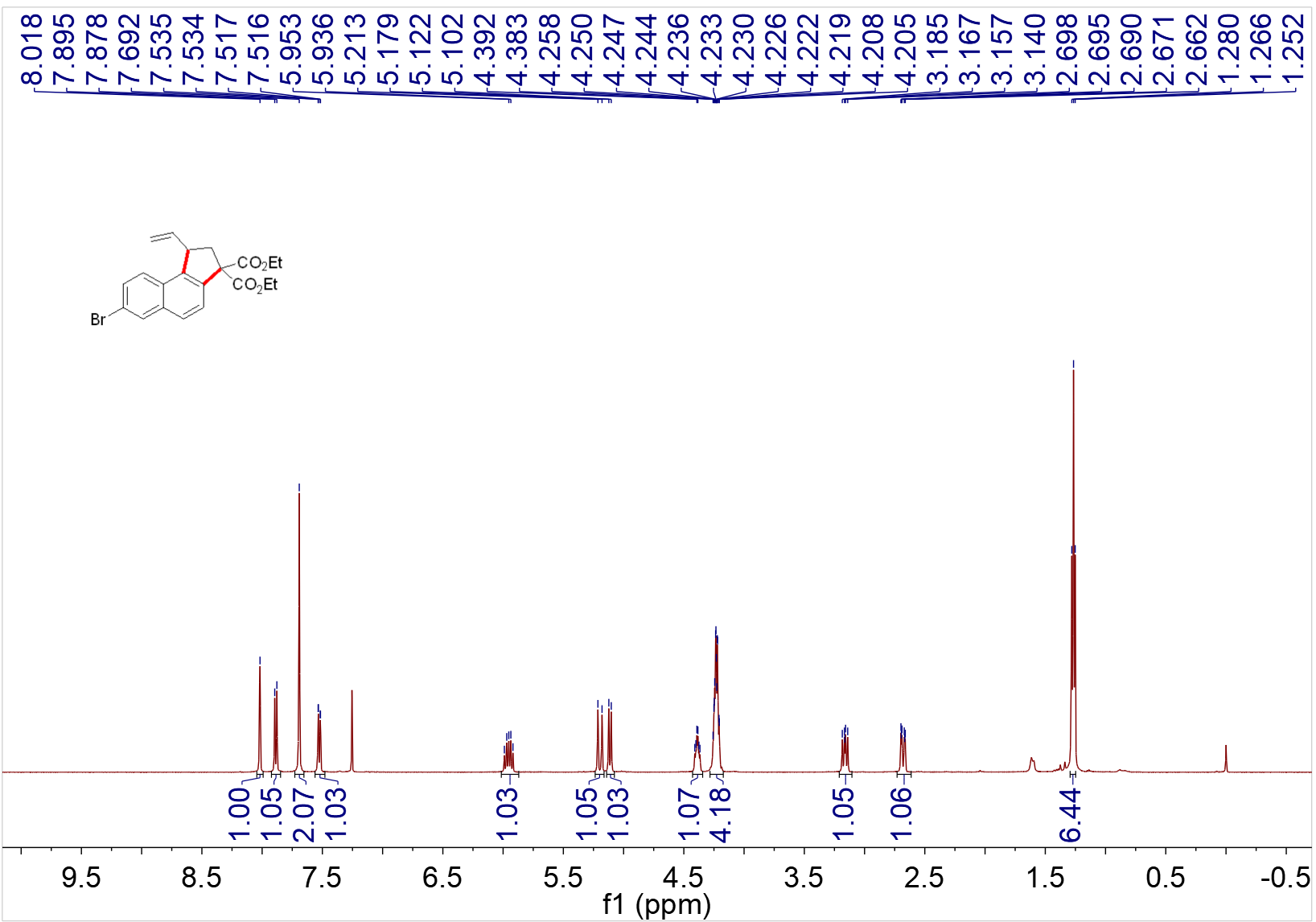


**Supplementary Figure 3. ^1^H NMR spectrum of 3b (500 MHz, CDCl_3_)**


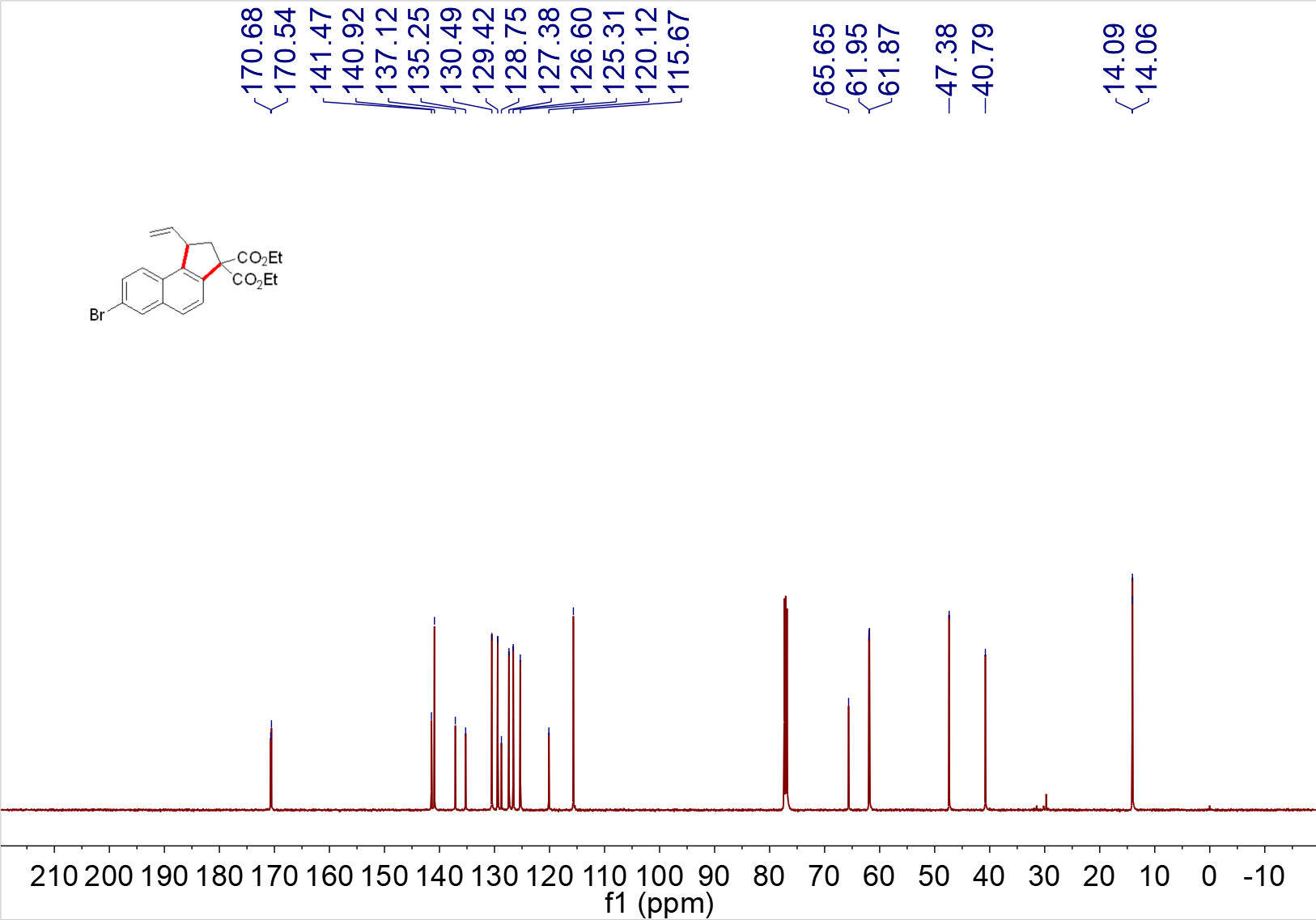


**Supplementary Figure 4. ^13^C NMR spectrum of 3b (125 MHz, CDCl_3_)**


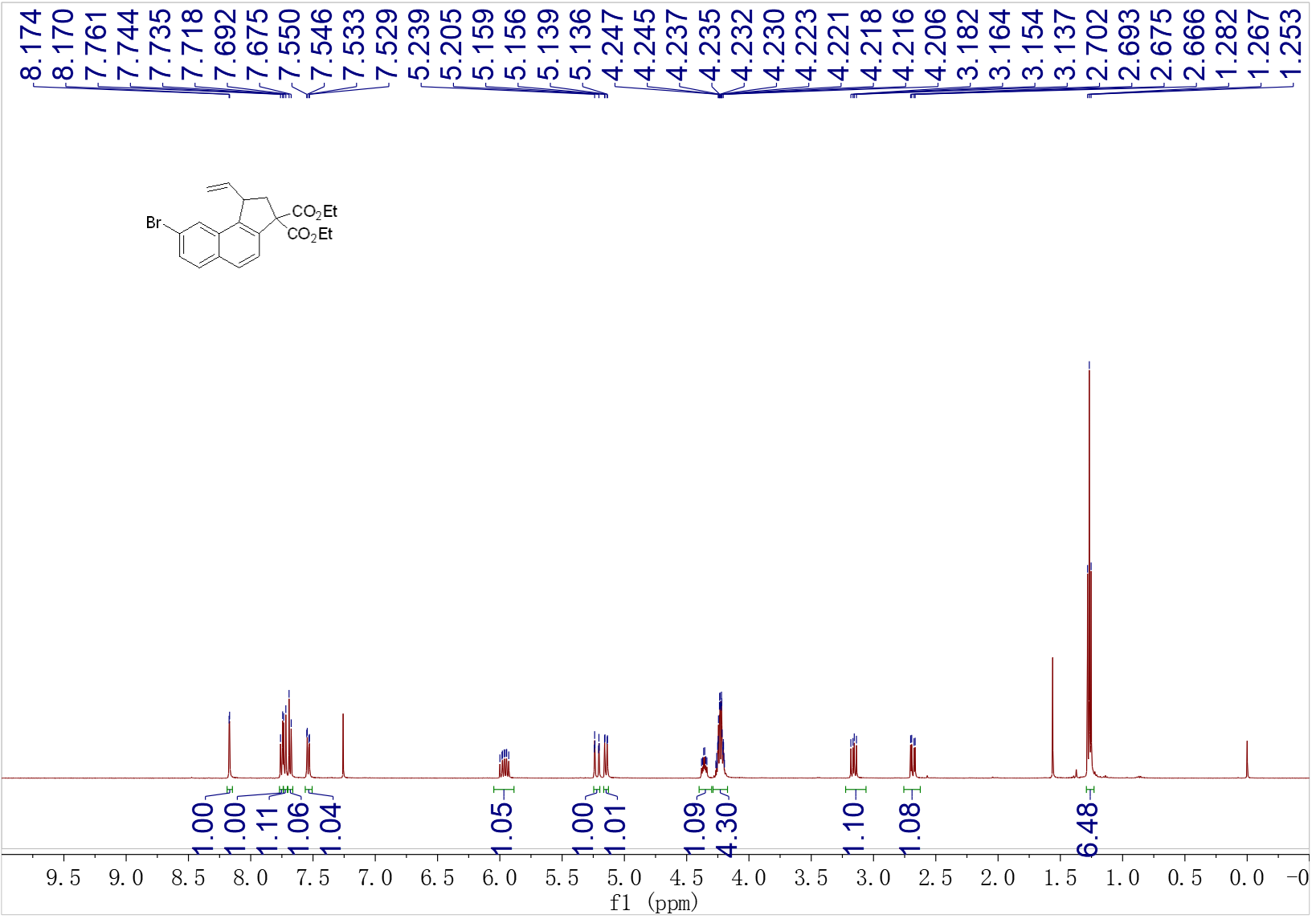


**Supplementary Figure 5. ^1^H NMR spectrum of 3d (500 MHz, CDCl_3_)**


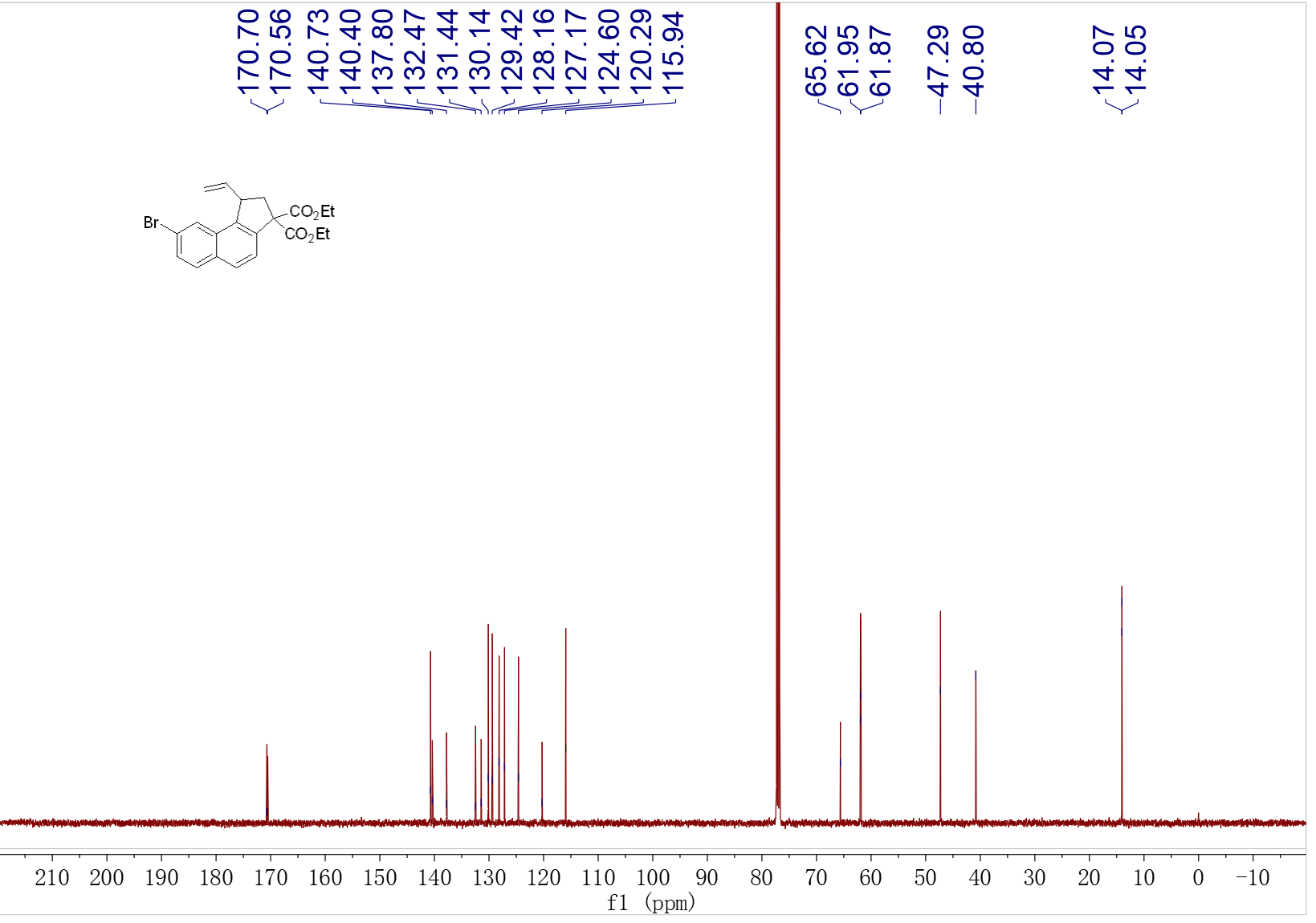


**Supplementary Figure 6. ^13^C NMR spectrum of 3d (125 MHz, CDCl_3_)**


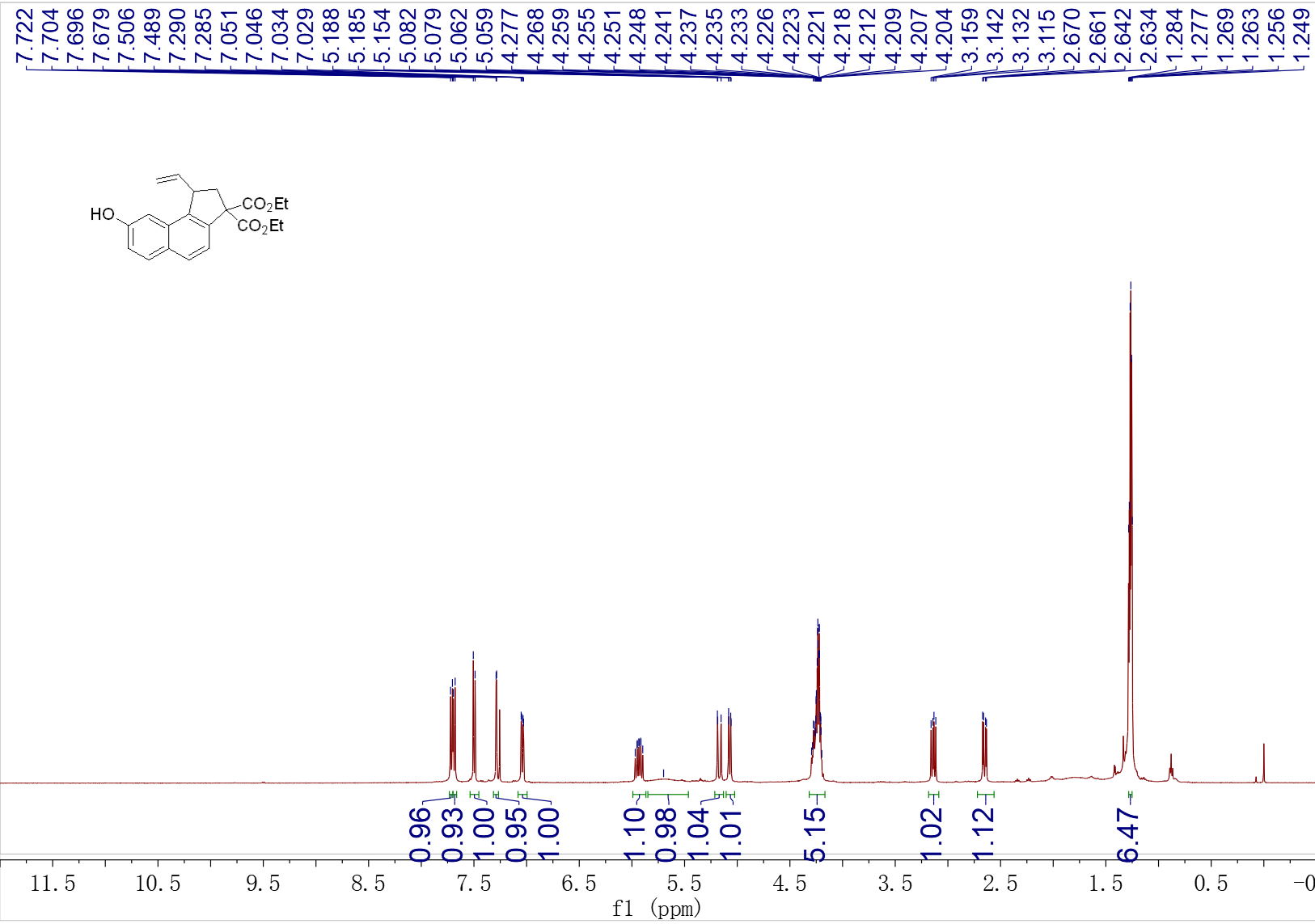


**Supplementary Figure 7. ^1^H NMR spectrum of 3e (500 MHz, CDCl_3_)**


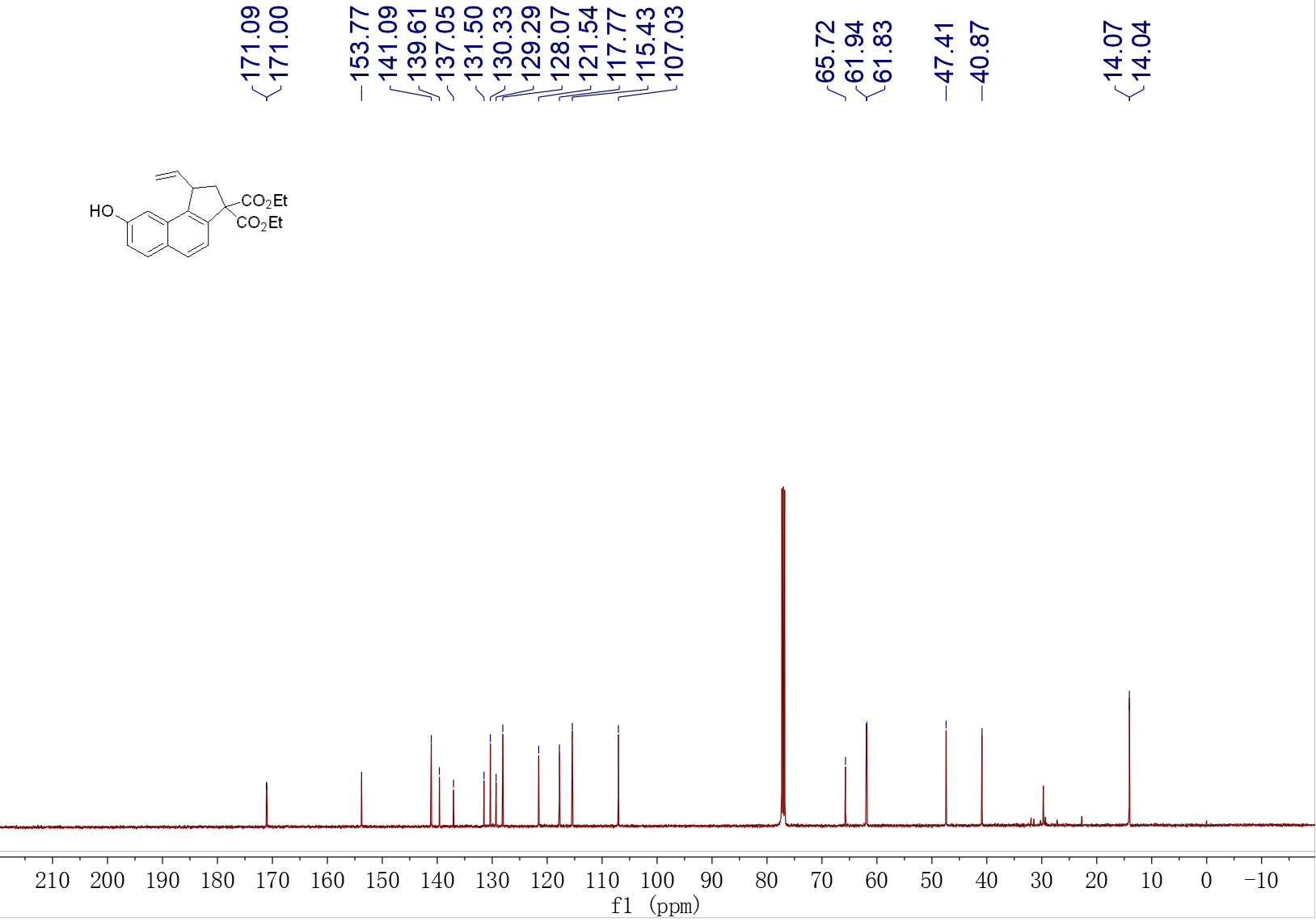


**Supplementary Figure 8. ^13^C NMR spectrum of 3e (125 MHz, CDCl_3_)**


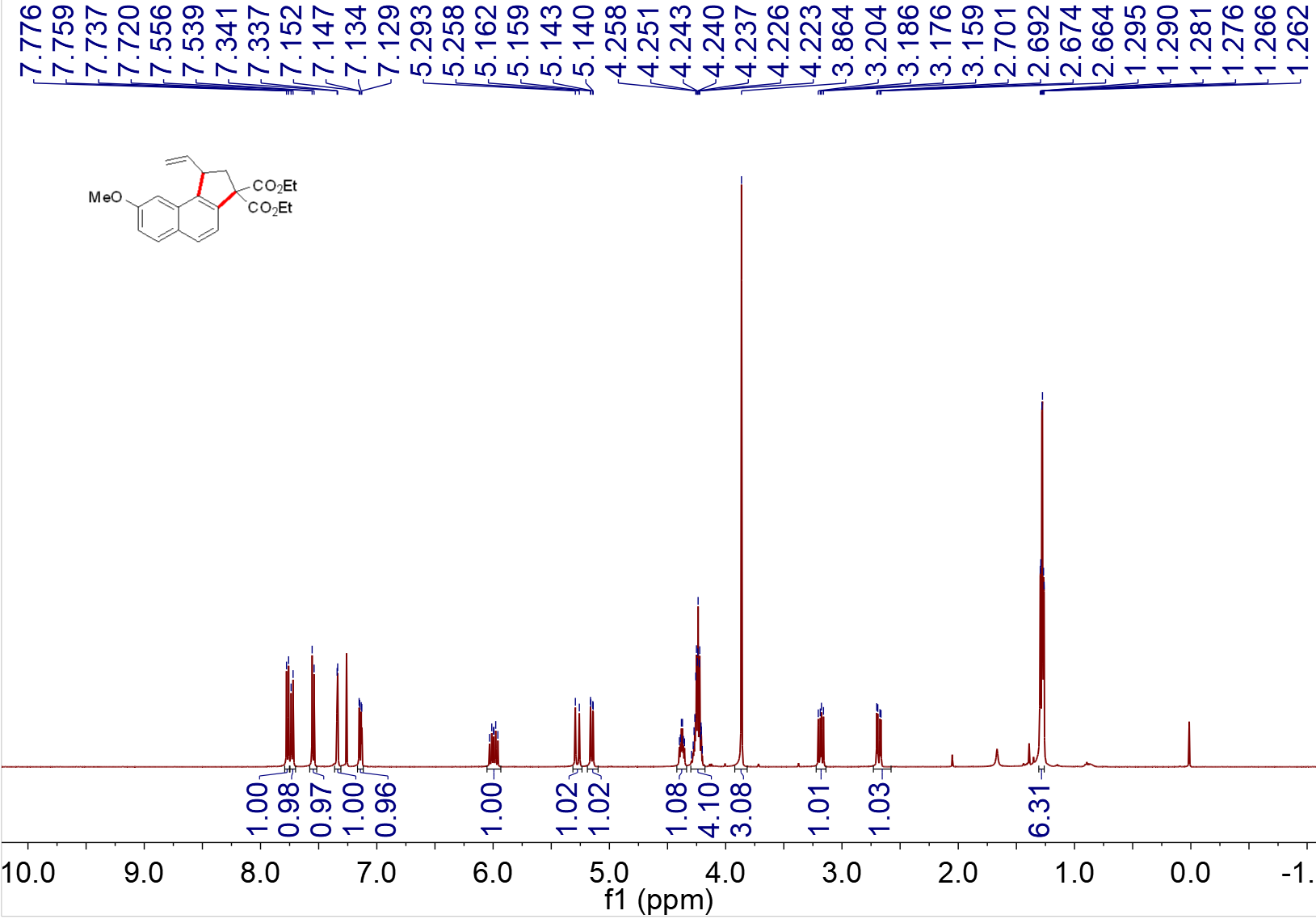


**Supplementary Figure 9. ^1^H NMR spectrum of 3f (500 MHz, CDCl_3_)**


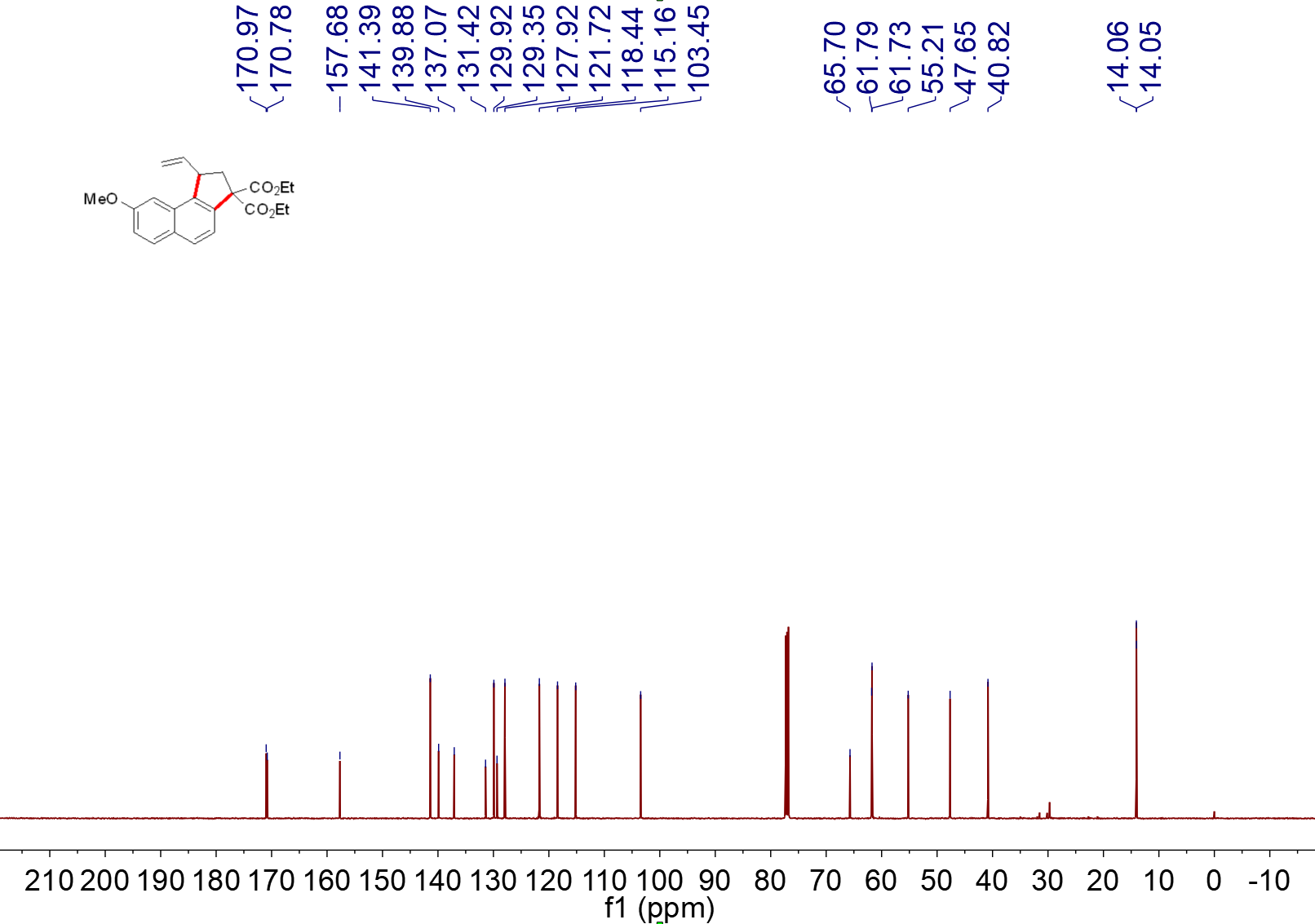


**Supplementary Figure 10. ^13^C NMR spectrum of 3f (125 MHz, CDCl_3_)**


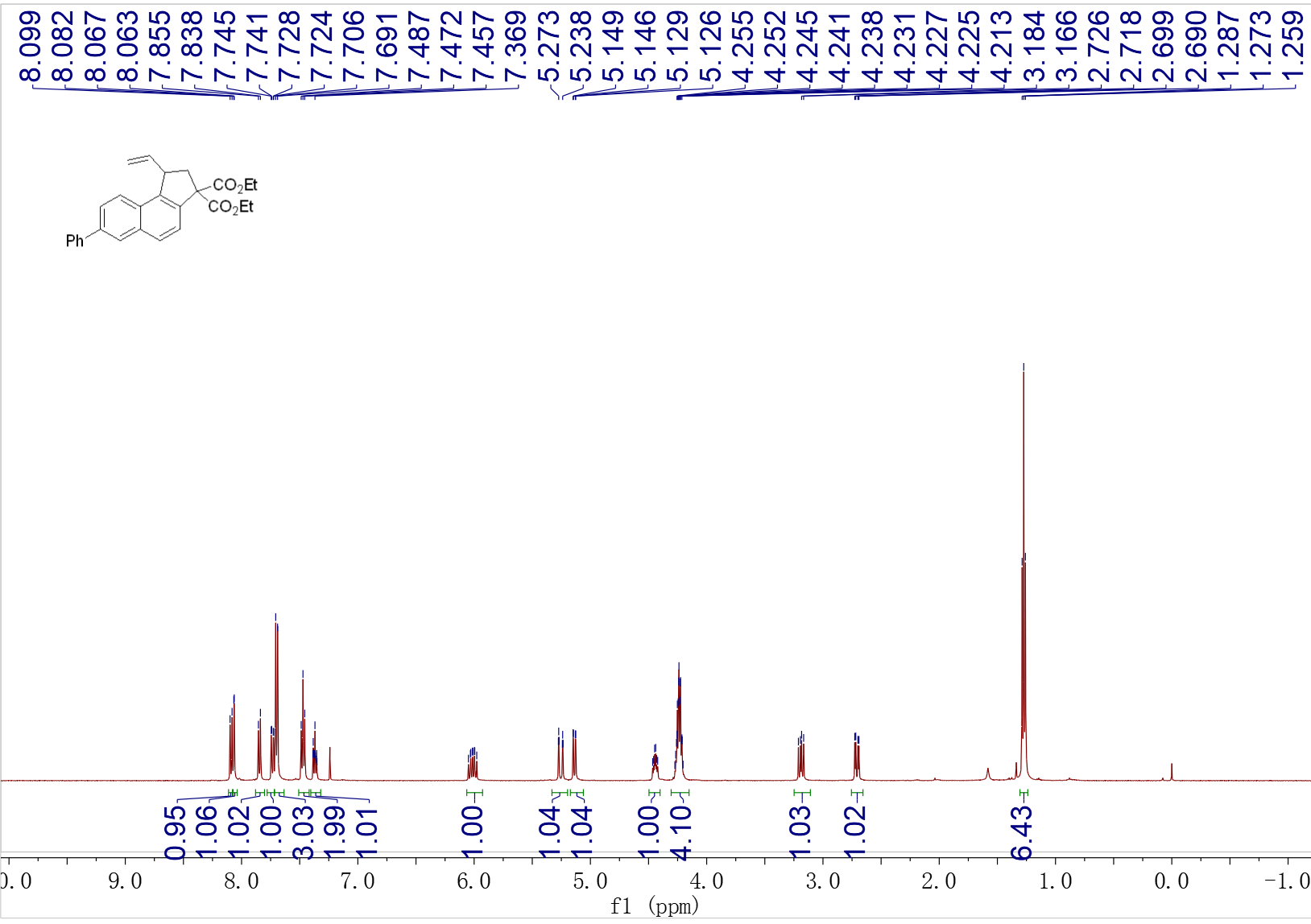


**Supplementary Figure 11. ^1^H NMR spectrum of 3g (500 MHz, CDCl_3_)**


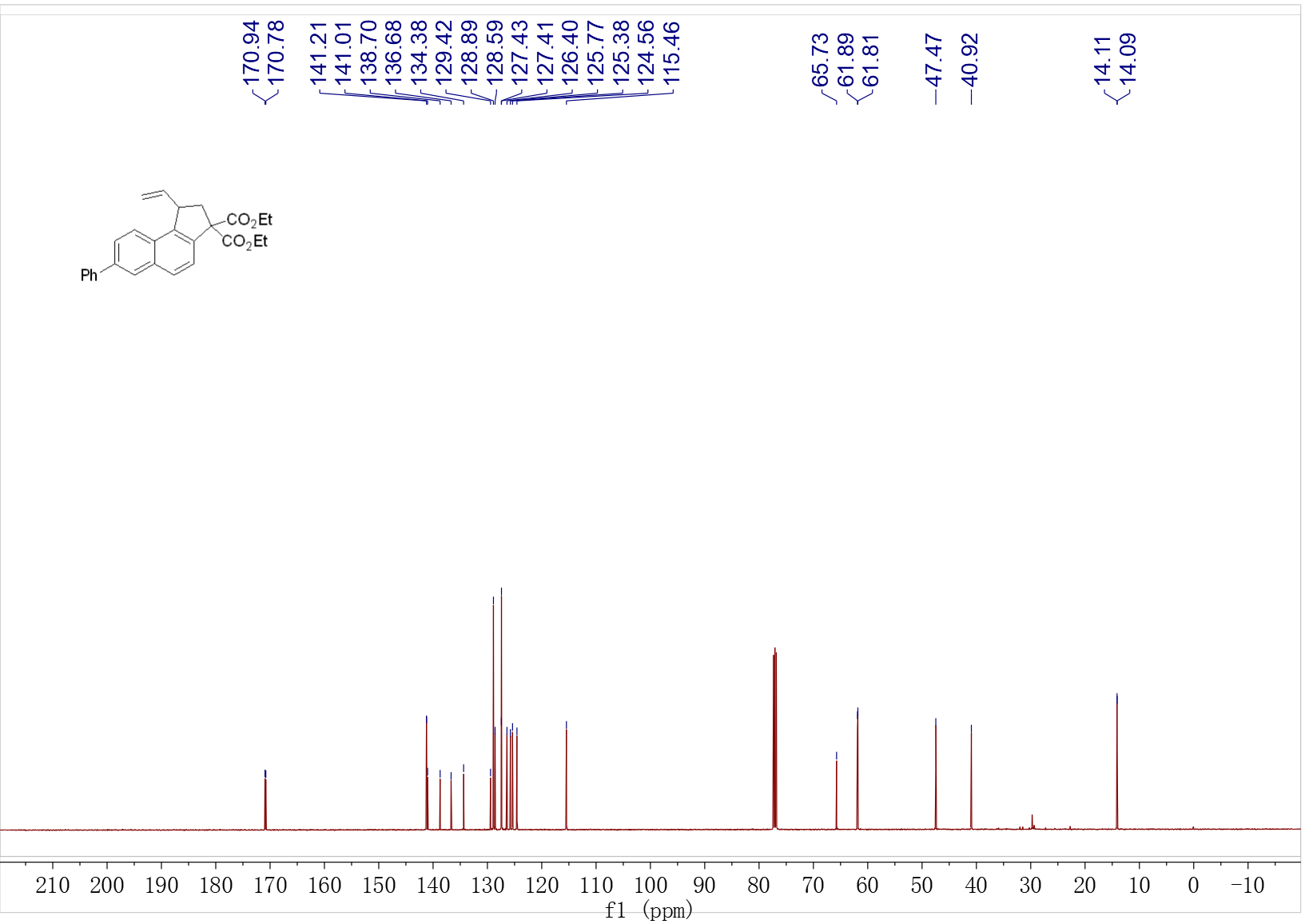


**Supplementary Figure 12. ^13^C NMR spectrum of 3g (125 MHz, CDCl_3_)**


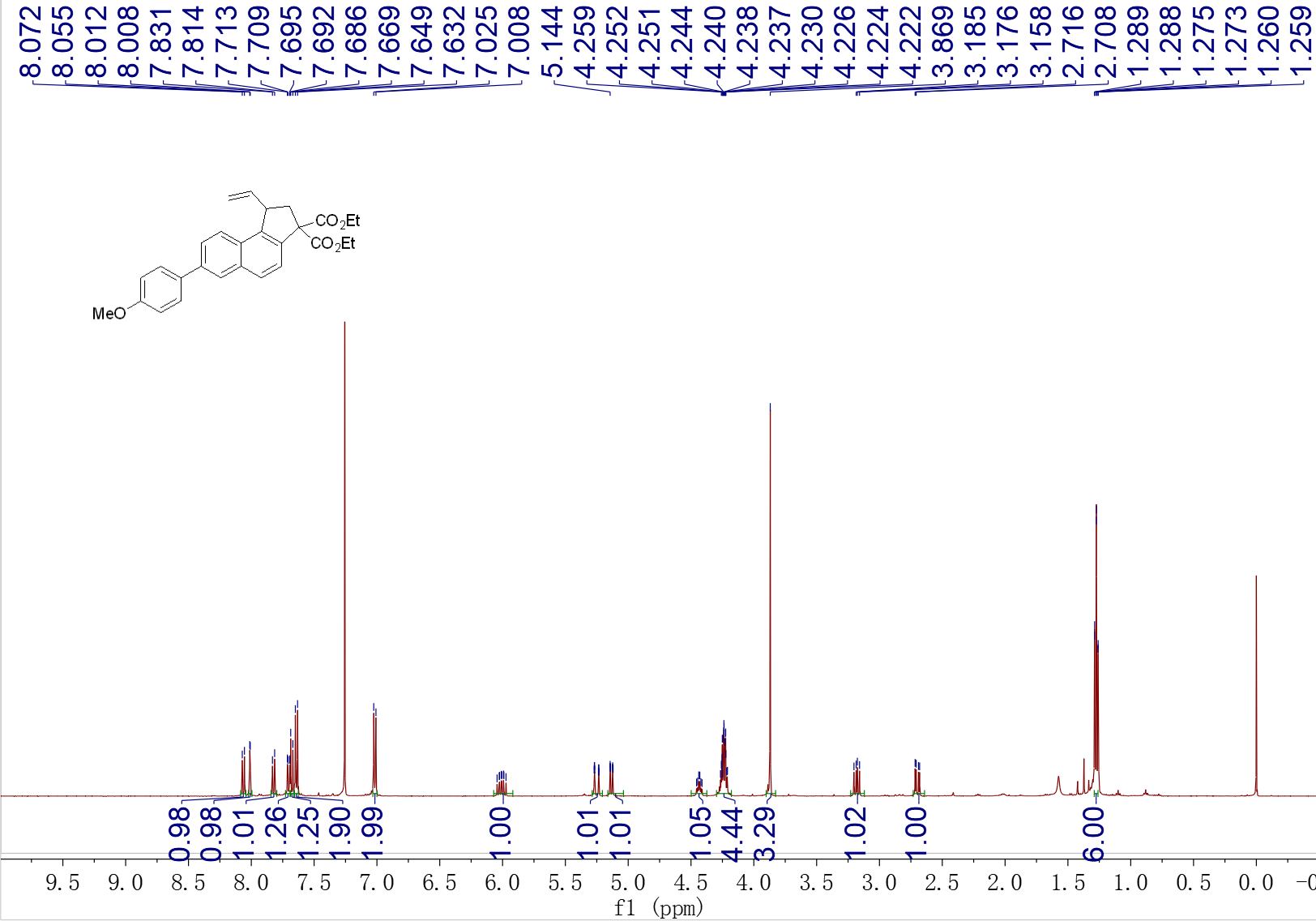


**Supplementary Figure 13. ^1^H NMR spectrum of 3h (500 MHz, CDCl_3_)**


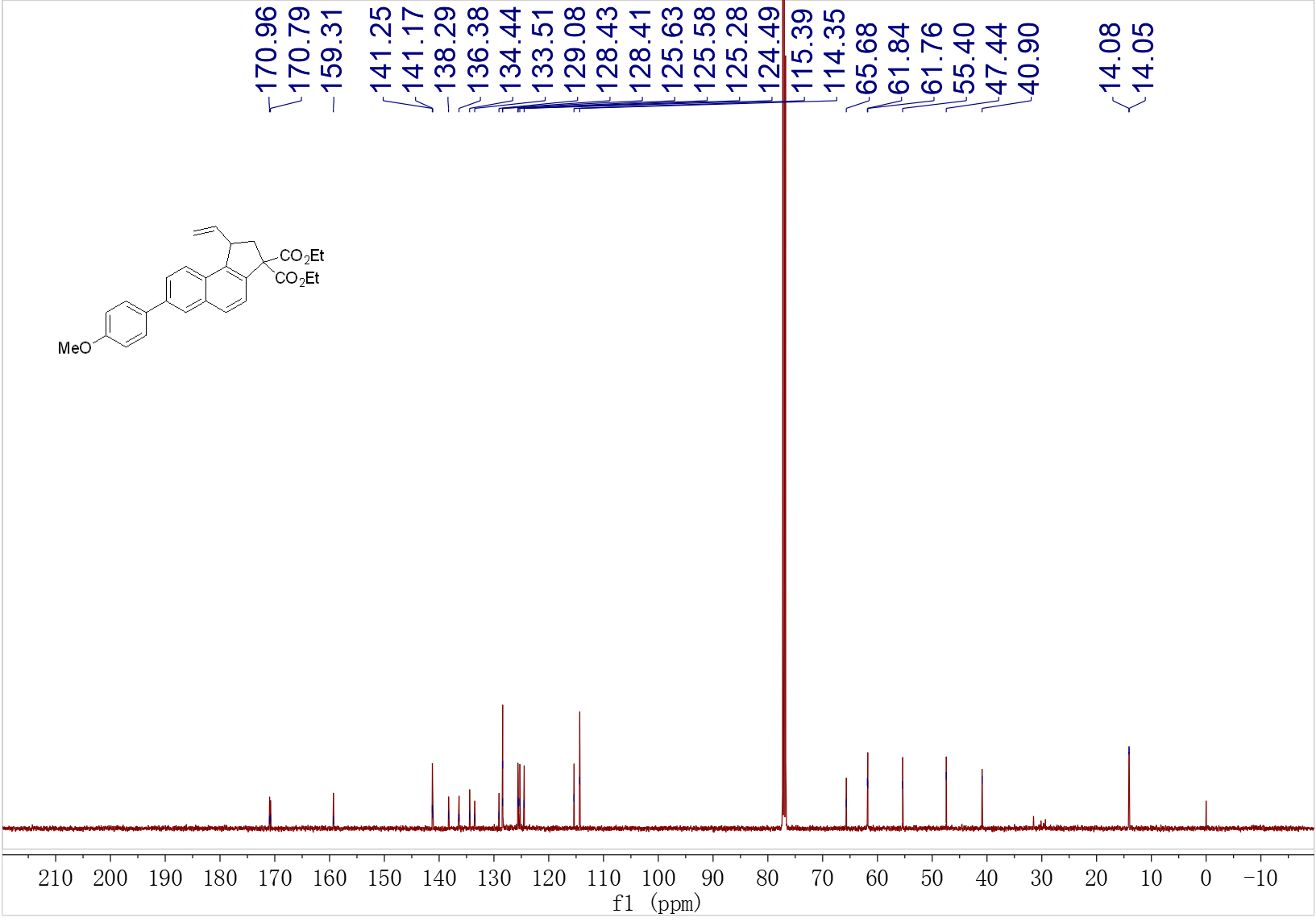


**Supplementary Figure 14. ^13^C NMR spectrum of 3h (125 MHz, CDCl_3_)**


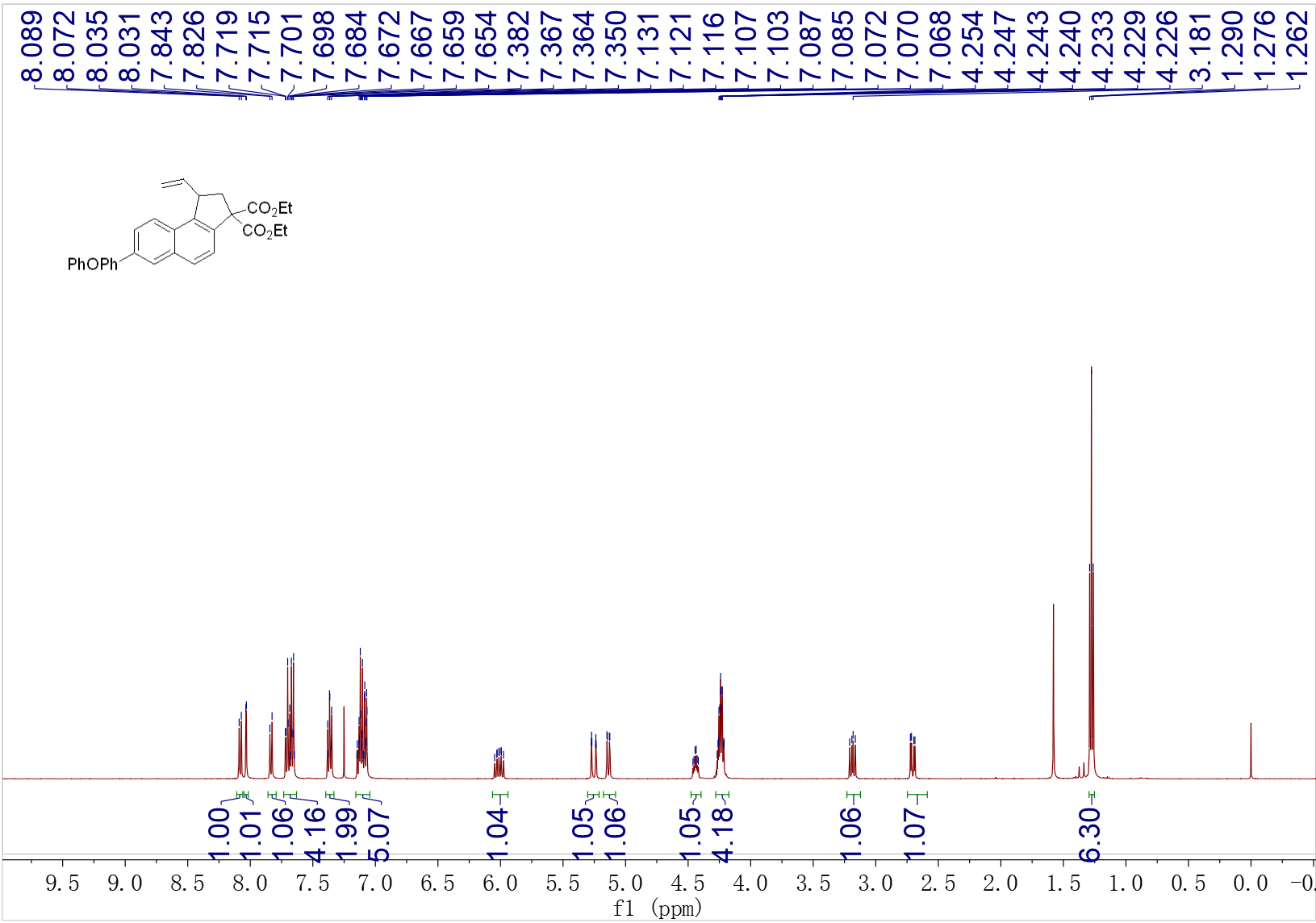


**Supplementary Figure 15. ^1^H NMR spectrum of 3i (500 MHz, CDCl_3_)**


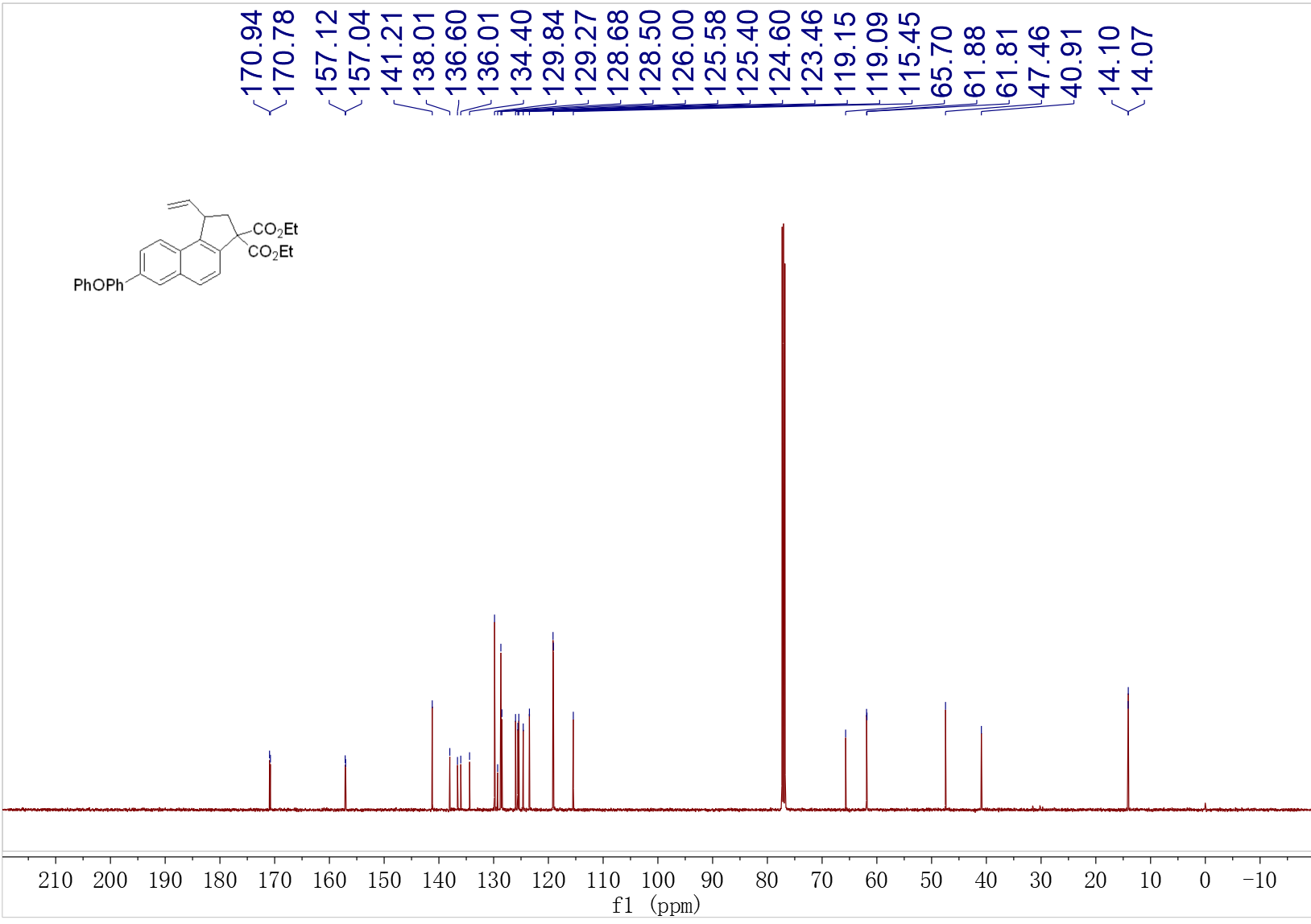


**Supplementary Figure 16. ^13^C NMR spectrum of 3i (125 MHz, CDCl_3_)**


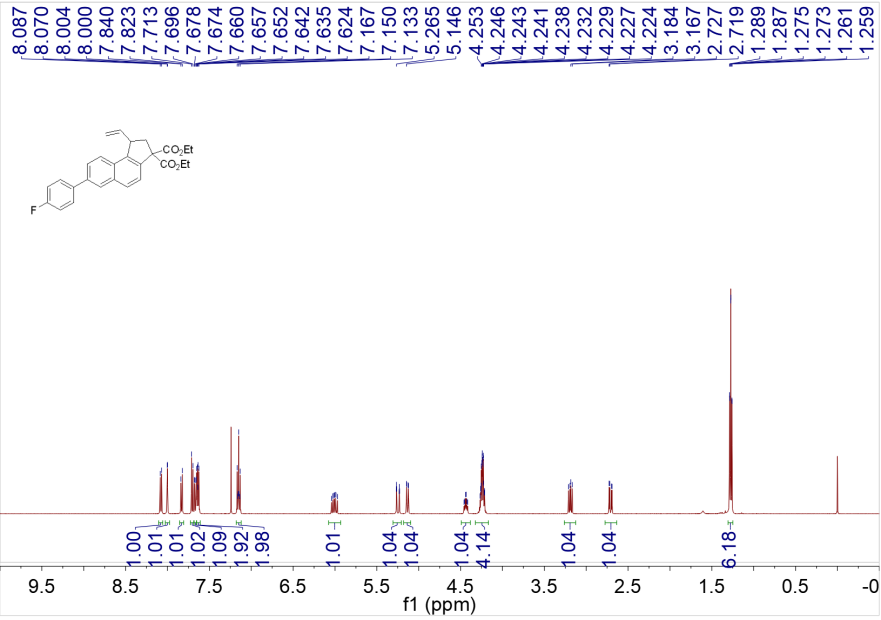


**Supplementary Figure 17. ^1^H NMR spectrum of 3j (500 MHz, CDCl_3_)**


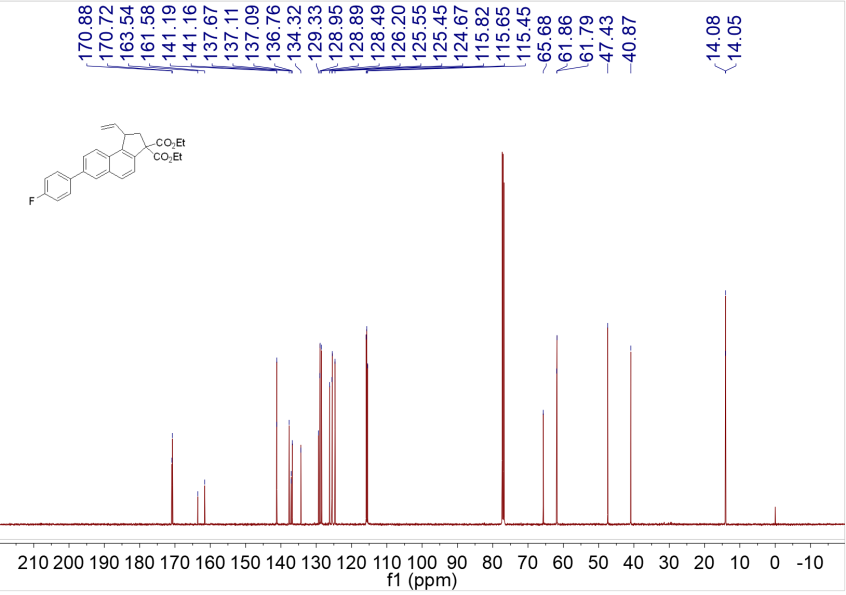


**Supplementary Figure 18. ^13^C NMR spectrum of 3j (125 MHz, CDCl_3_)**


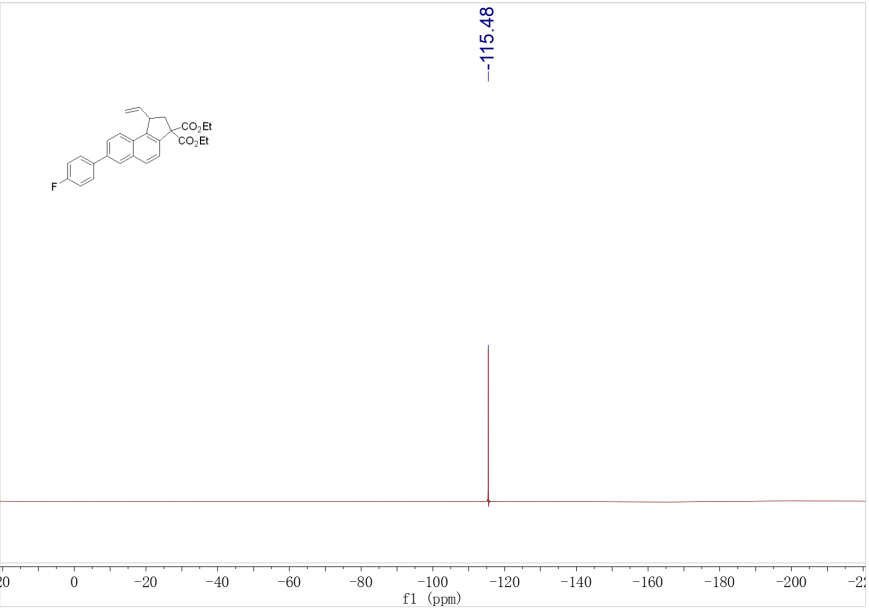


**Supplementary Figure 19. ^19^F NMR spectrum of 3j (470 MHz, CDCl_3_)**


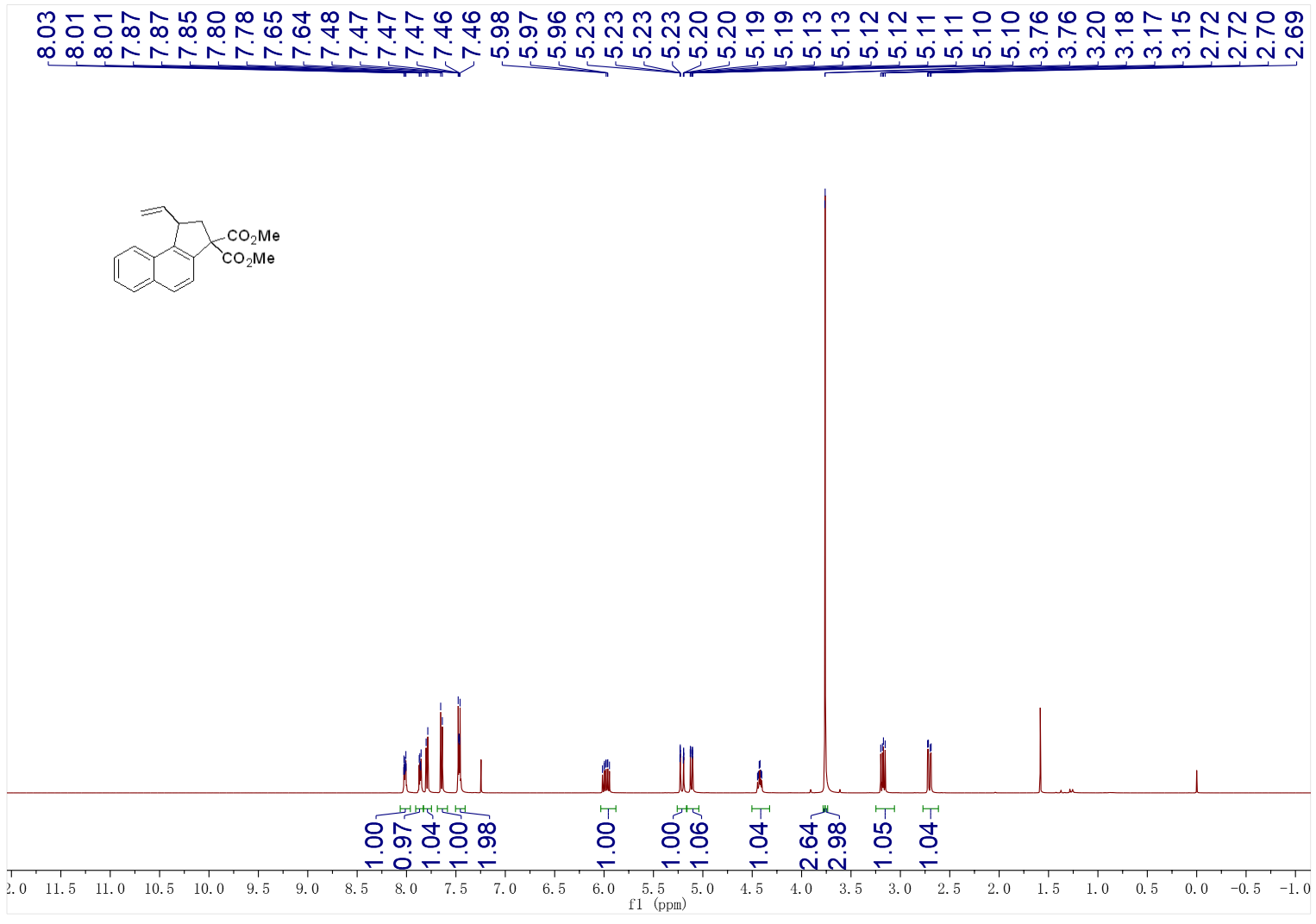


**Supplementary Figure 20. ^1^H NMR spectrum of 3k (500 MHz, CDCl_3_)**


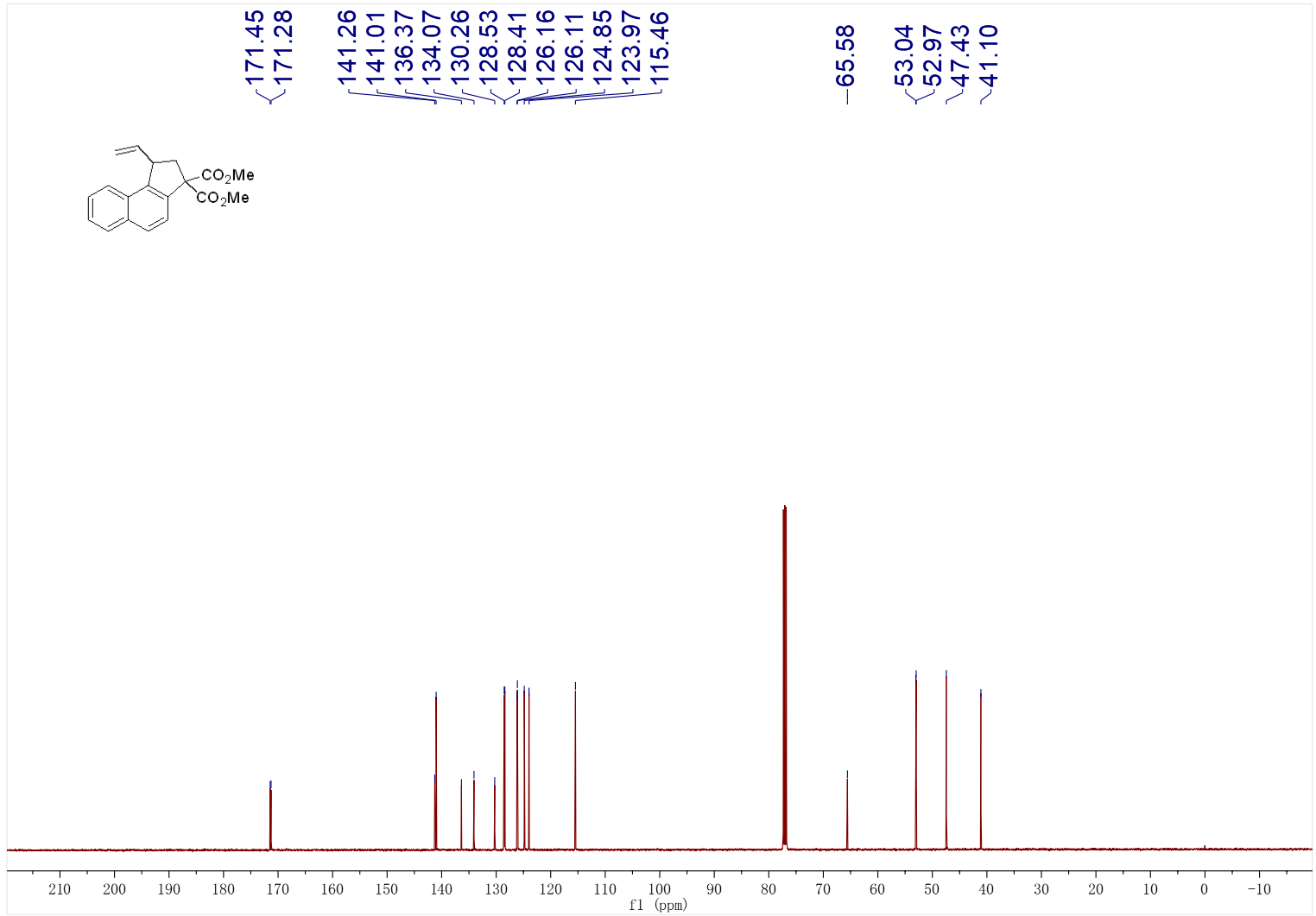


**Supplementary Figure 21. ^13^C spectrum NMR of 3k (125 MHz, CDCl_3_)**


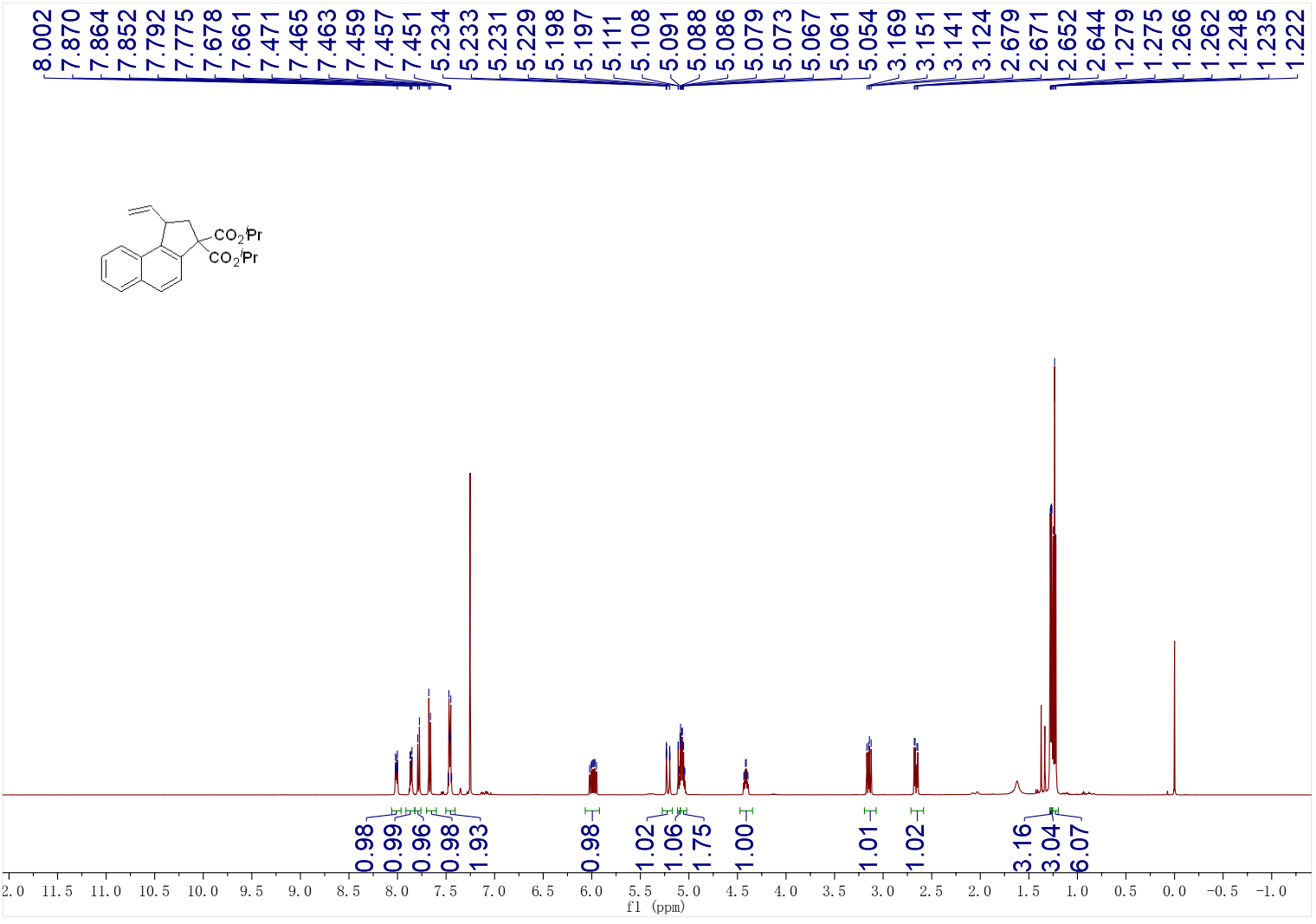


**Supplementary Figure 22. ^1^H NMR spectrum of 3l (500 MHz, CDCl_3_)**


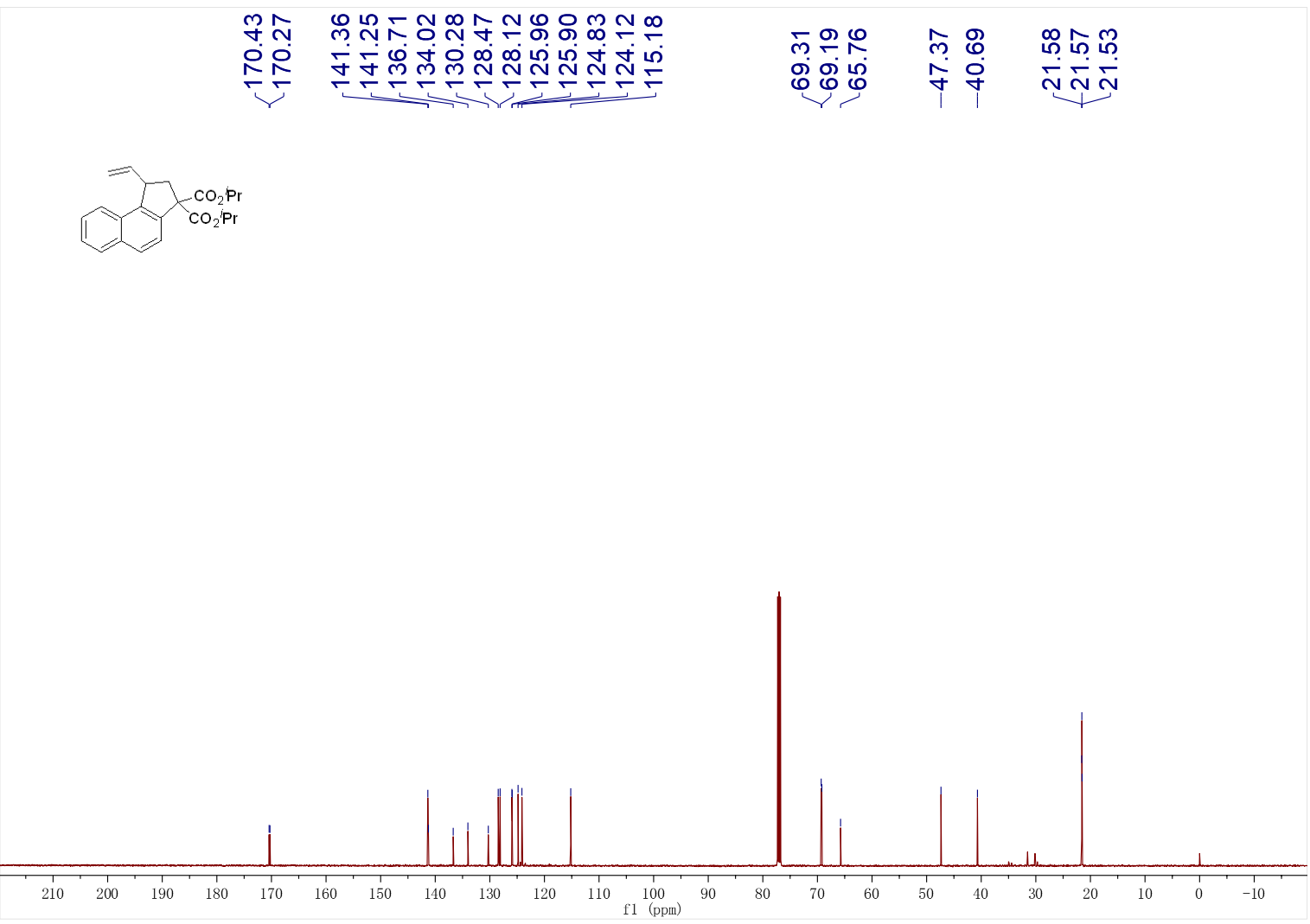


**Supplementary Figure 23. ^13^C NMR spectrum of 3l (125 MHz, CDCl_3_)**


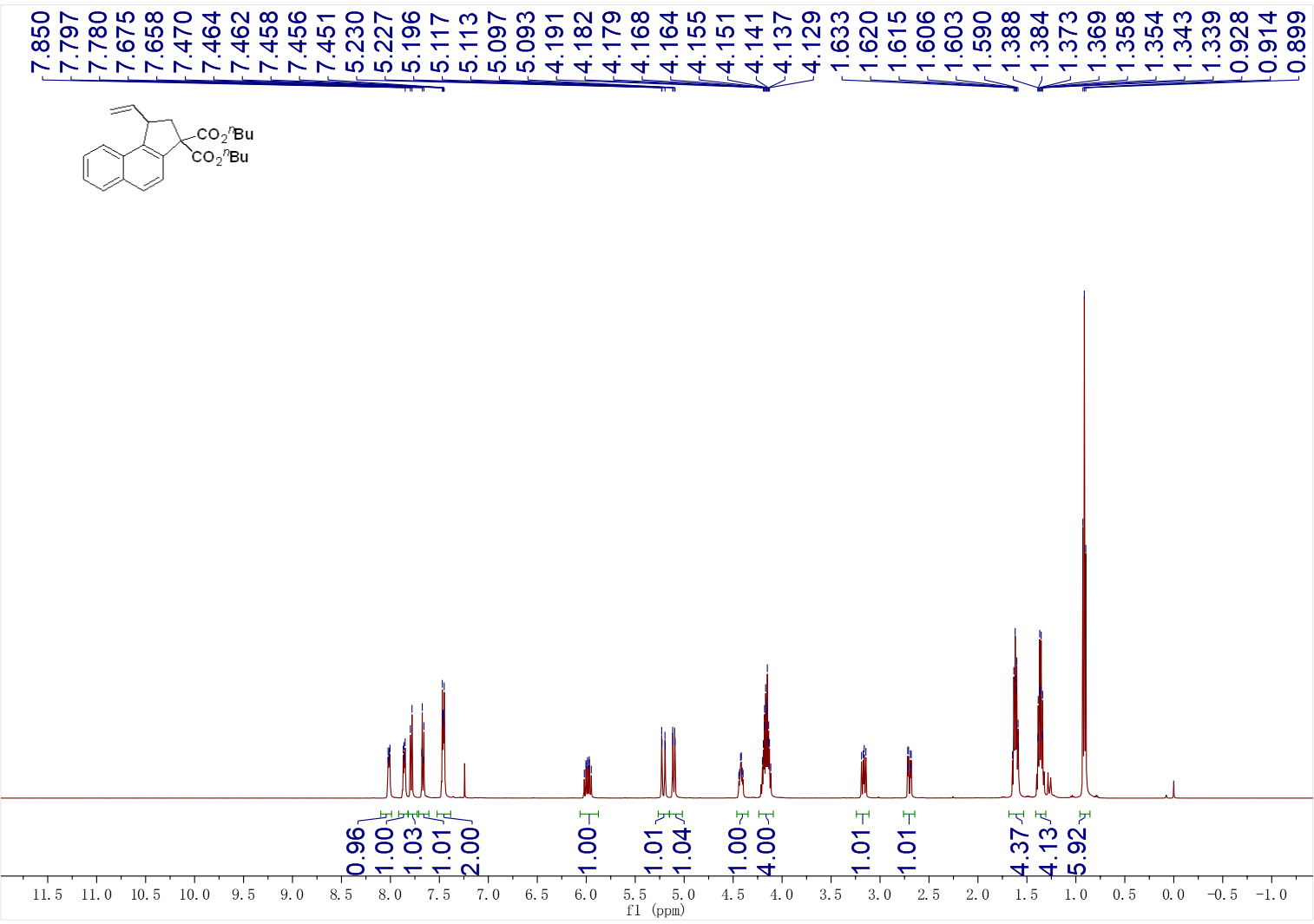


**Supplementary Figure 24. ^1^H NMR spectrum of 3n (500 MHz, CDCl_3_)**


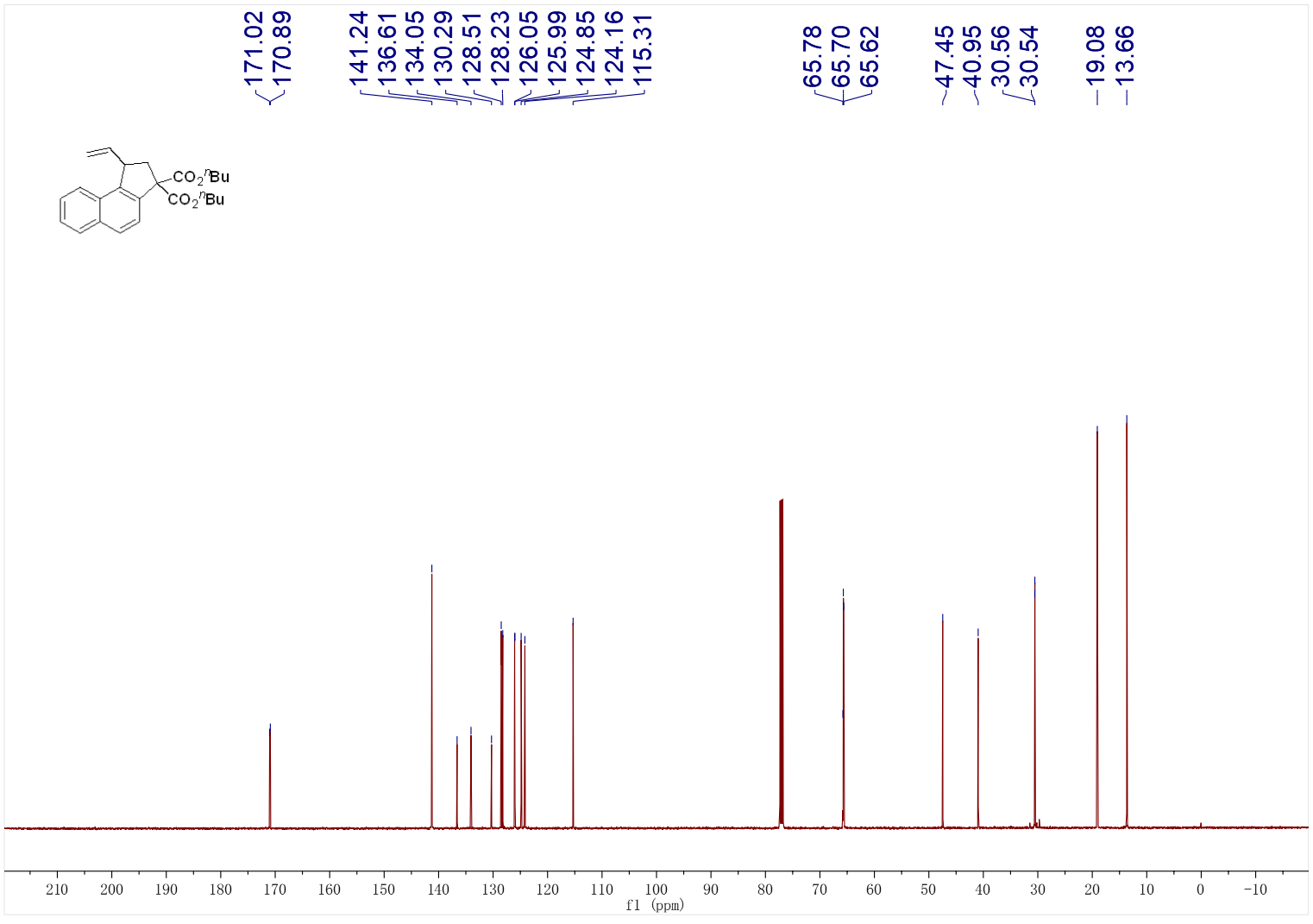


**Supplementary Figure 25. ^13^C NMR spectrum of 3n (125 MHz, CDCl_3_)**


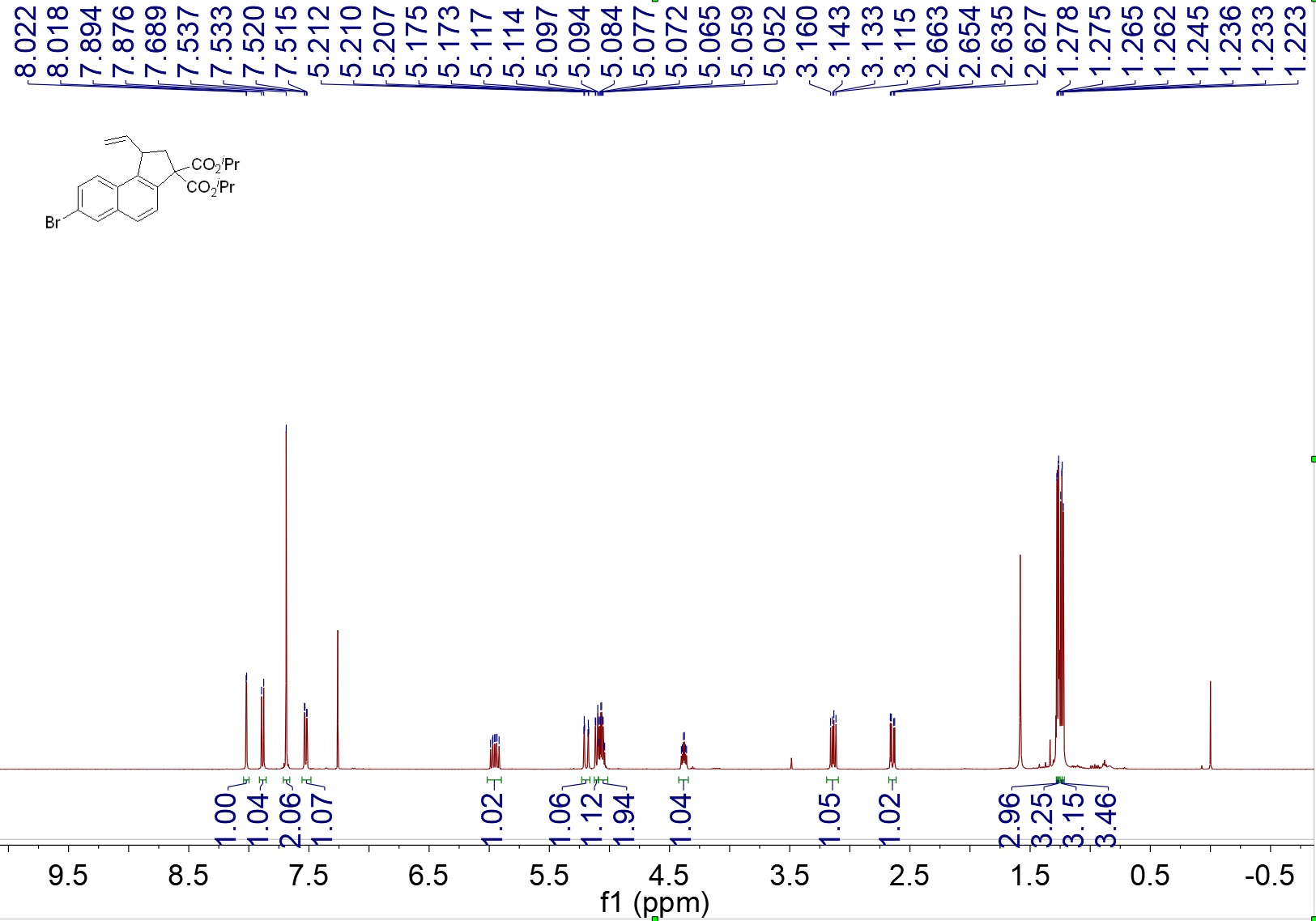


**Supplementary Figure 26. ^1^H NMR spectrum of 3o (500 MHz, CDCl_3_)**


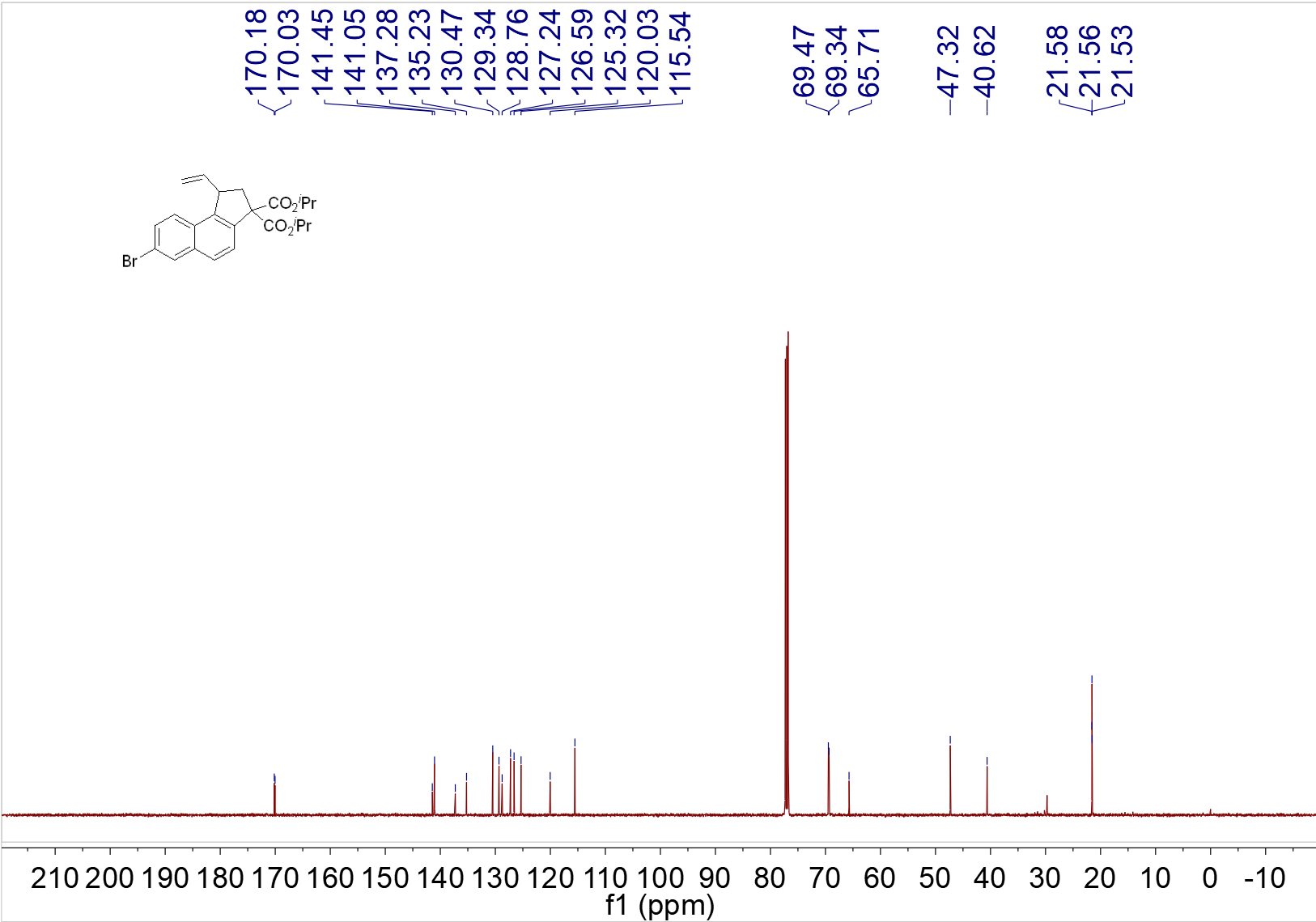


**Supplementary Figure 27. ^13^C NMR spectrum of 3o (125 MHz, CDCl_3_)**


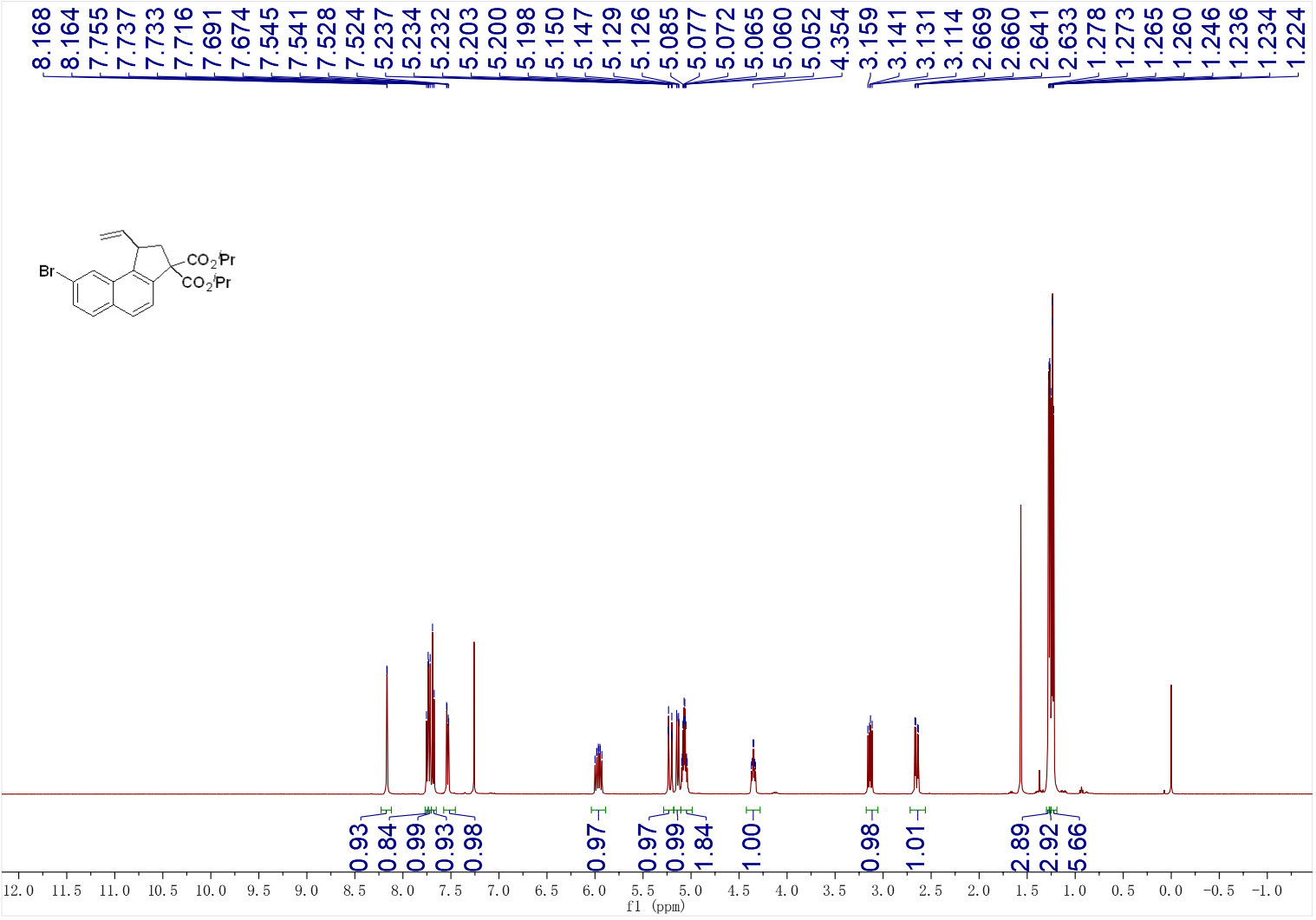


**Supplementary Figure 28. ^1^H NMR spectrum of 3p (500 MHz, CDCl_3_)**


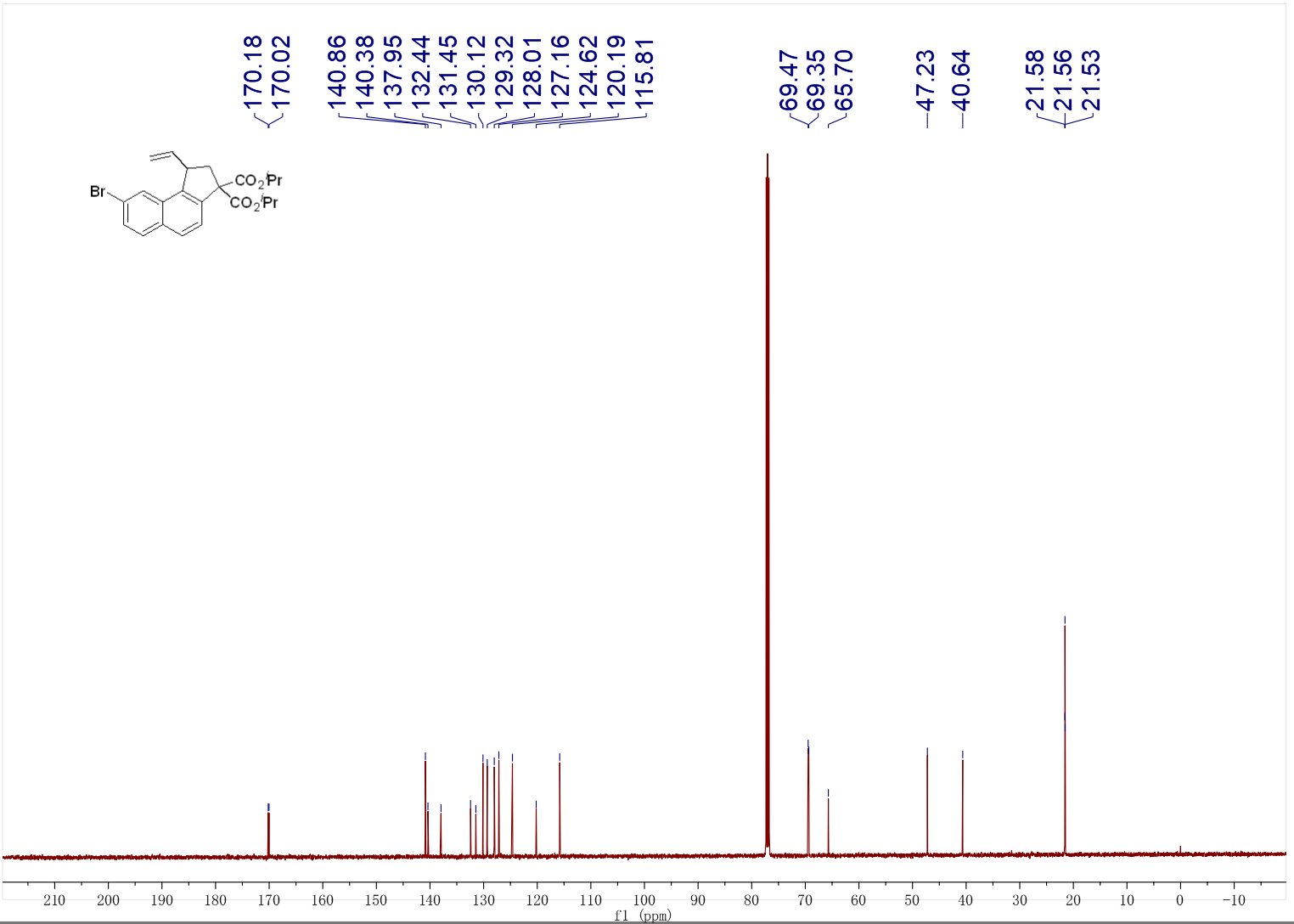


**Supplementary Figure 29. ^13^C NMR spectrum of 3p (125 MHz, CDCl_3_)**


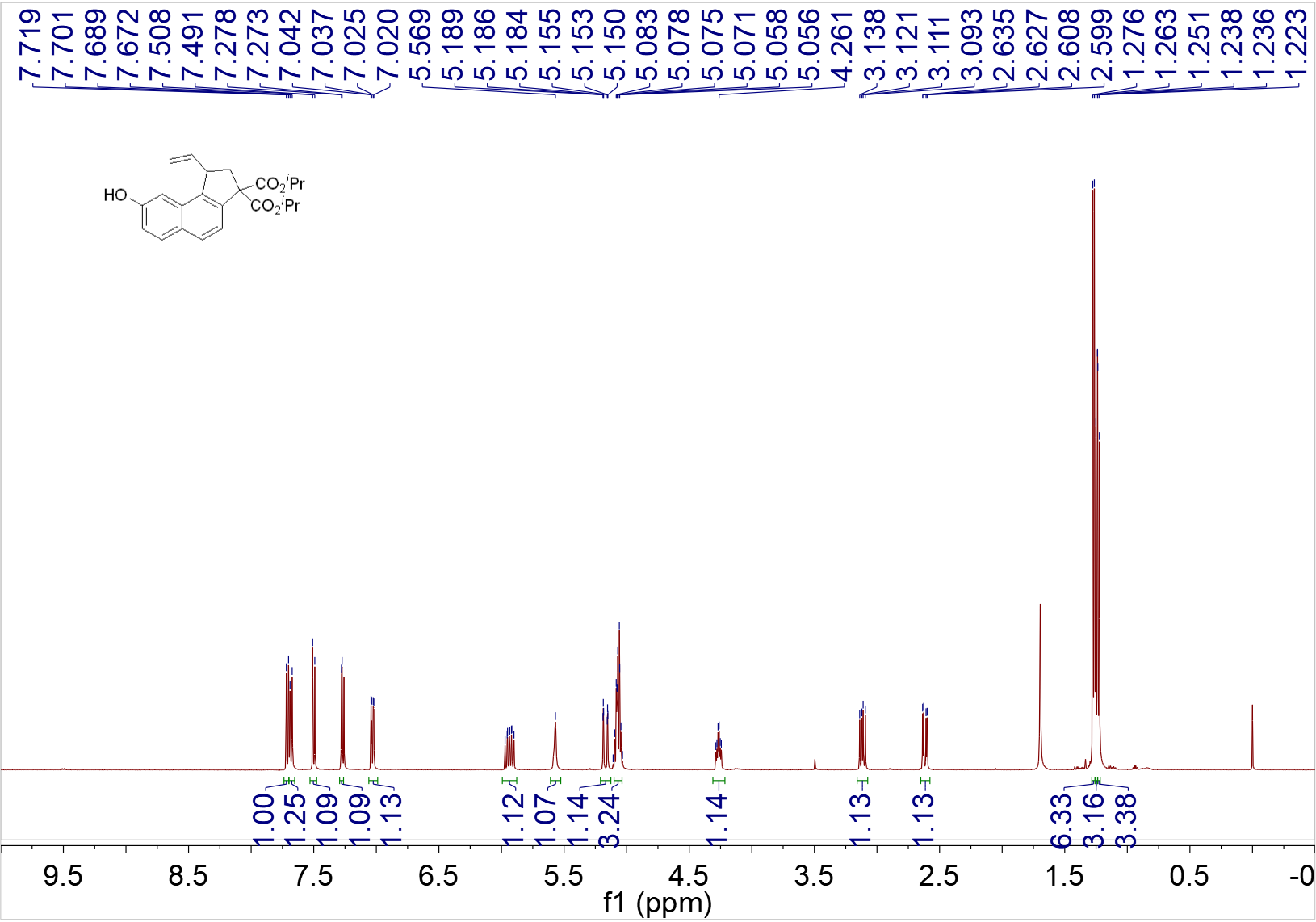


**Supplementary Figure 30. ^1^H NMR spectrum of 3q (500 MHz, CDCl_3_)**


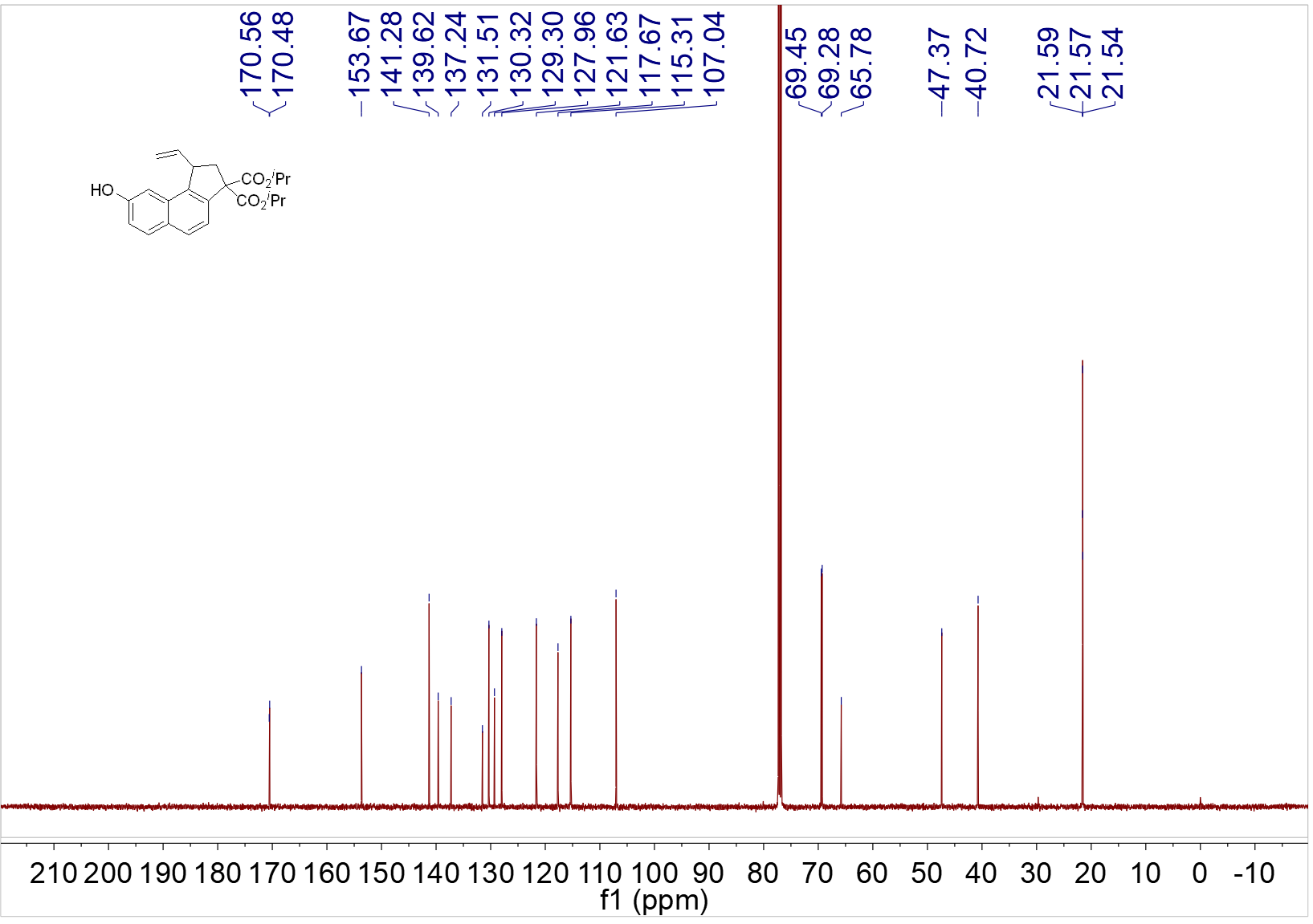


**Supplementary Figure 31. ^13^C NMR spectrum of 3q (125 MHz, CDCl_3_)**


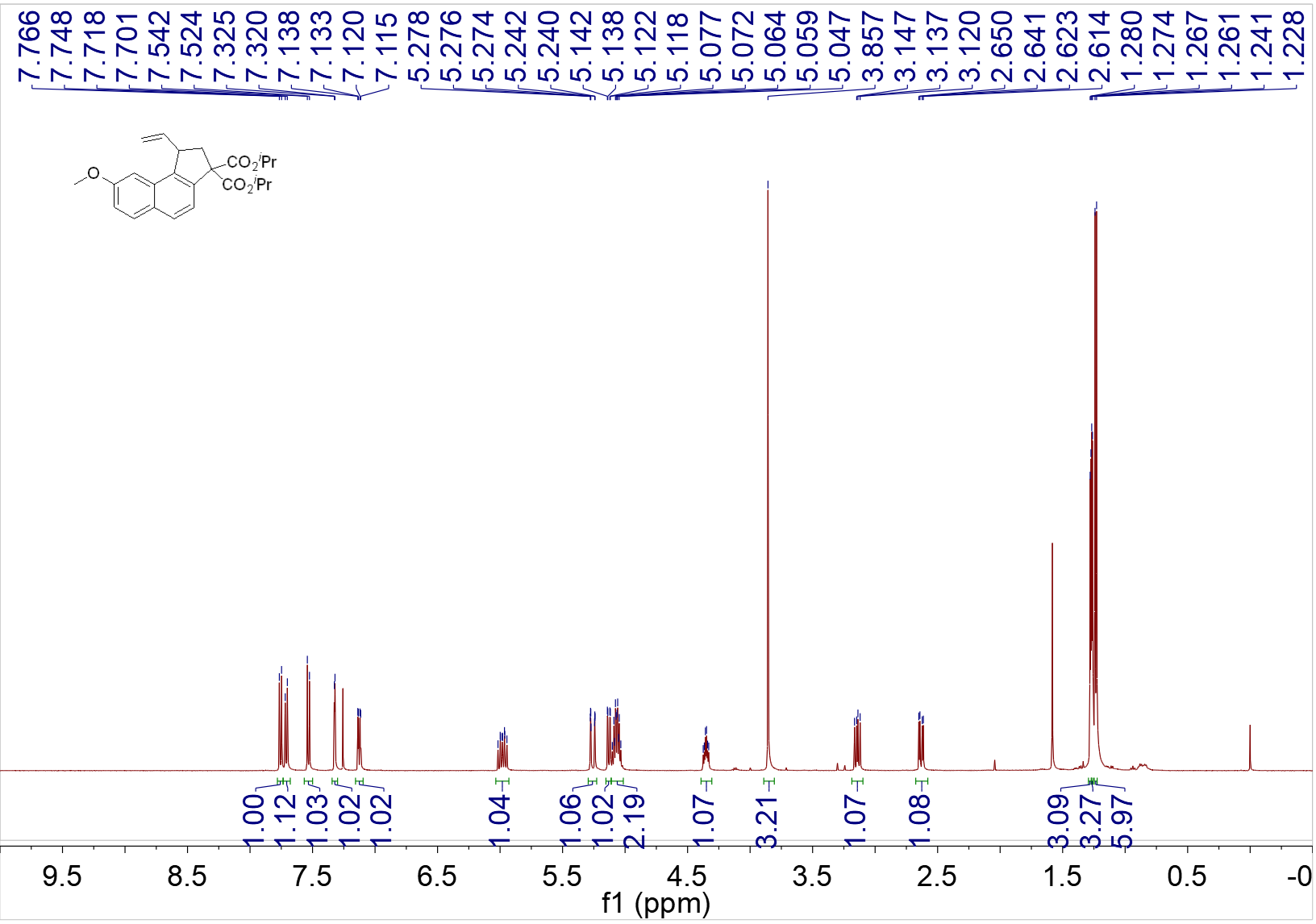


**Supplementary Figure 32. ^1^H NMR spectrum of 3r (500 MHz, CDCl_3_)**


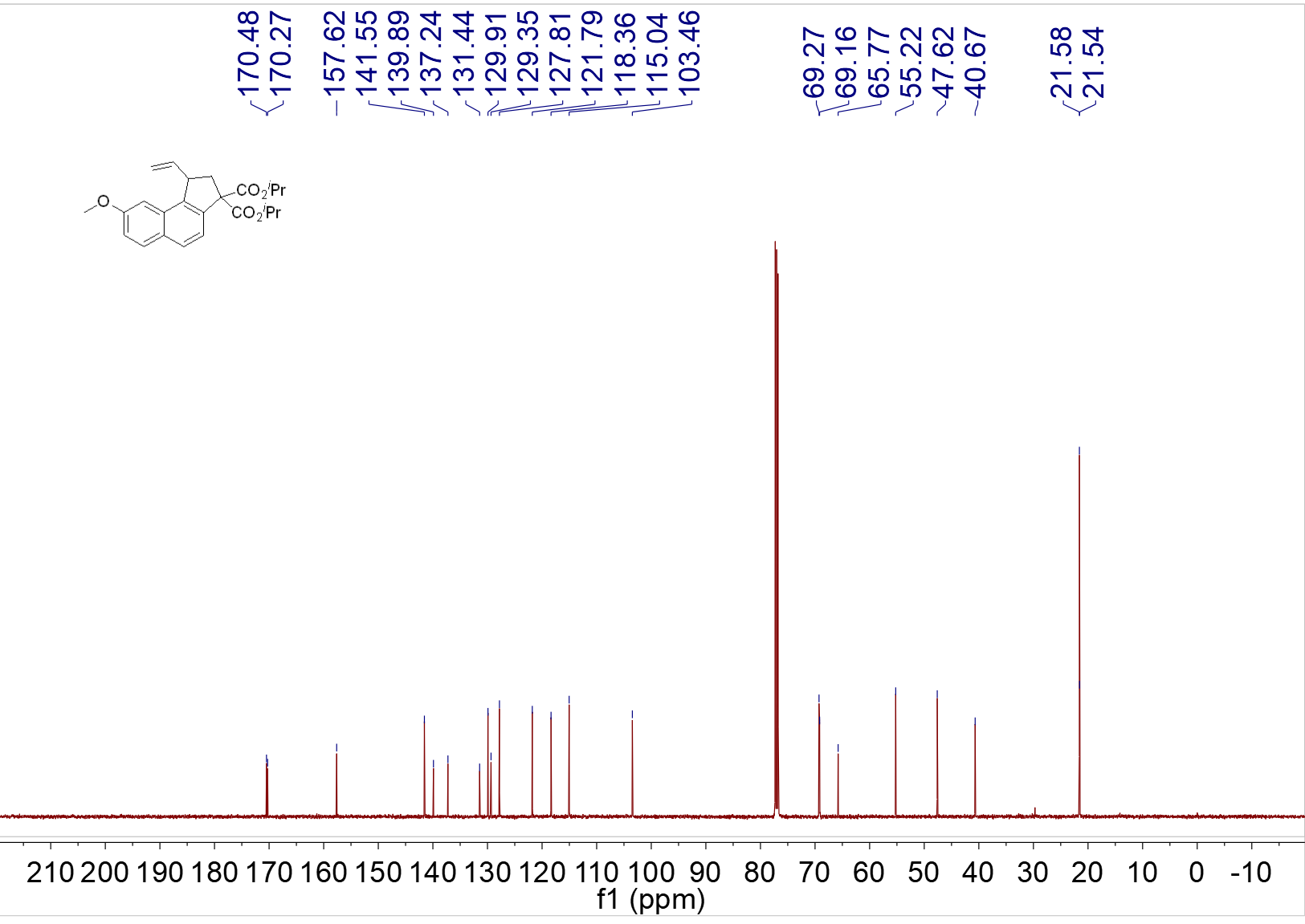


**Supplementary Figure 33. ^13^C NMR spectrum of 3r (125 MHz, CDCl_3_)**


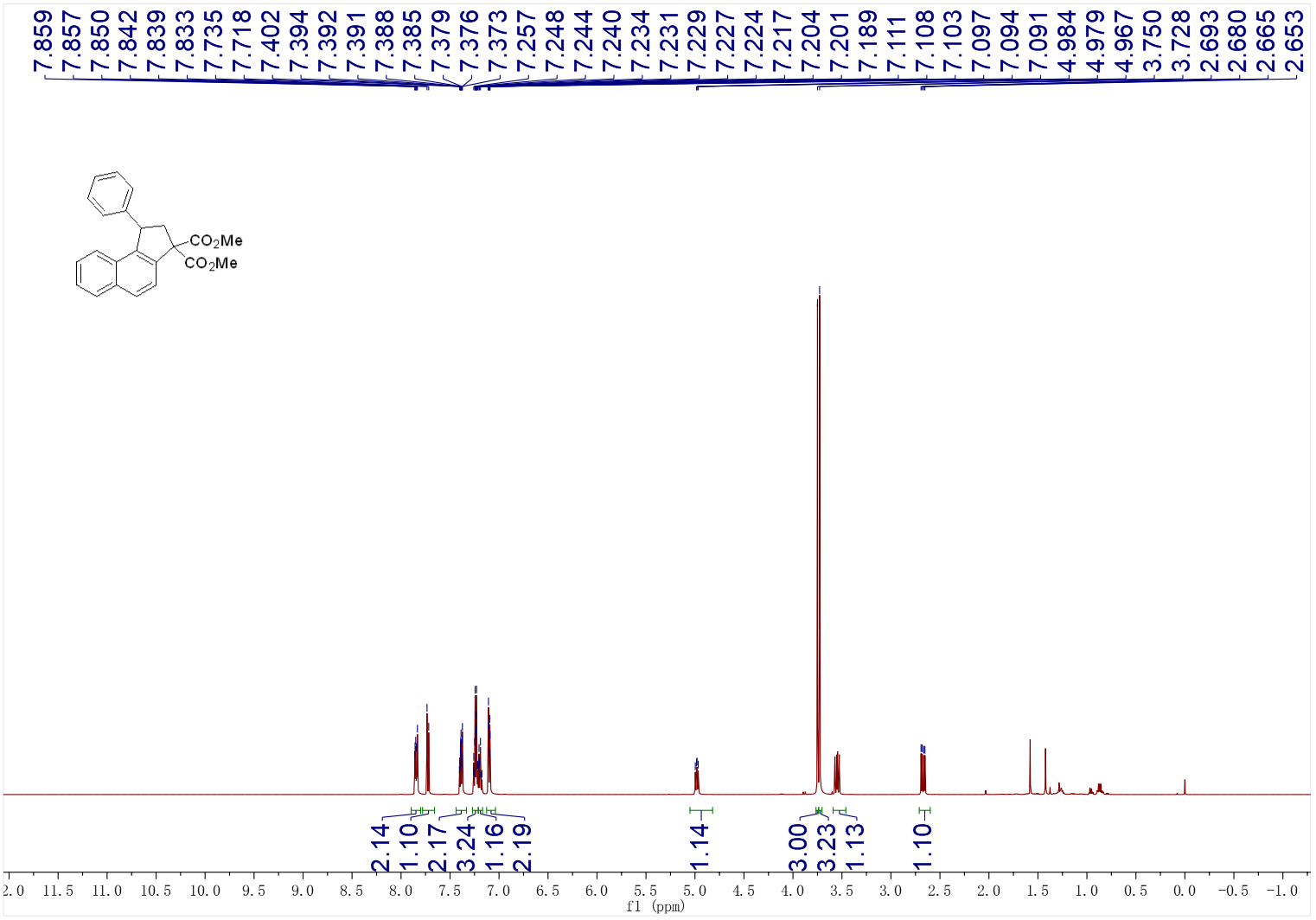


**Supplementary Figure 34. ^1^H NMR spectrum of 3s (500 MHz, CDCl_3_)**


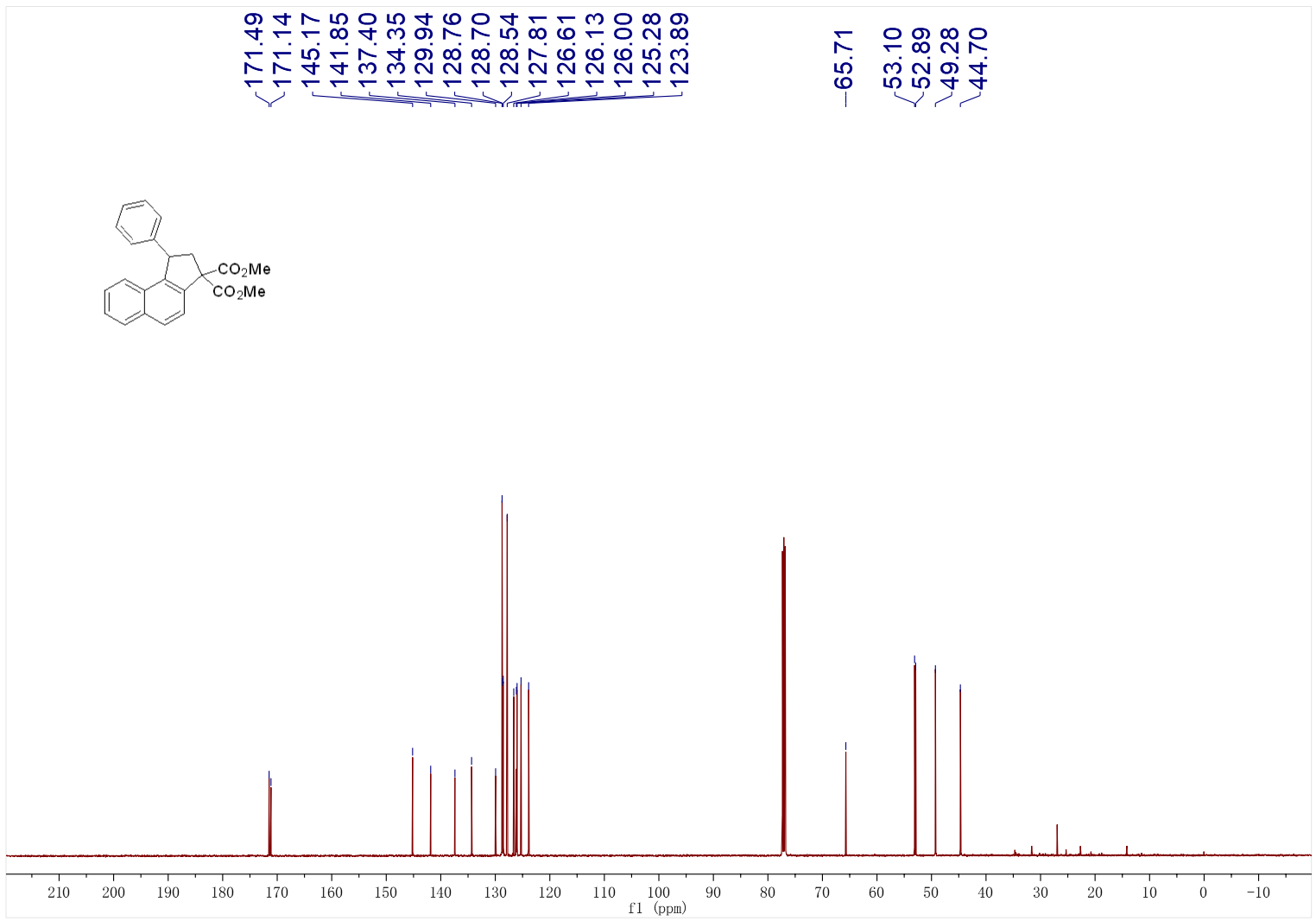


**Supplementary Figure 35. ^13^C NMR spectrum of 3s (125 MHz, CDCl_3_)**


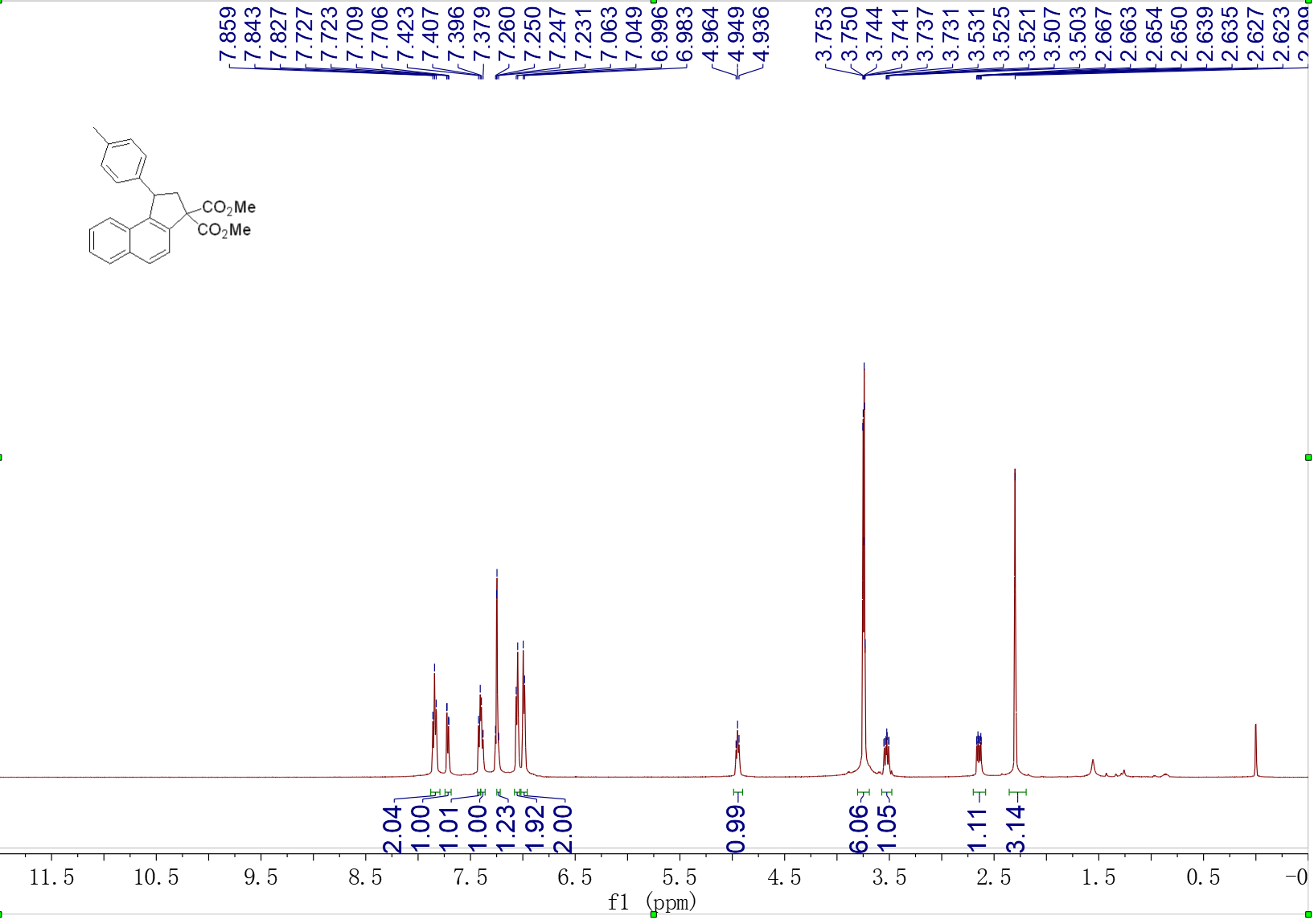


**Supplementary Figure 36. ^1^H NMR spectrum of 3t (500 MHz, CDCl_3_)**


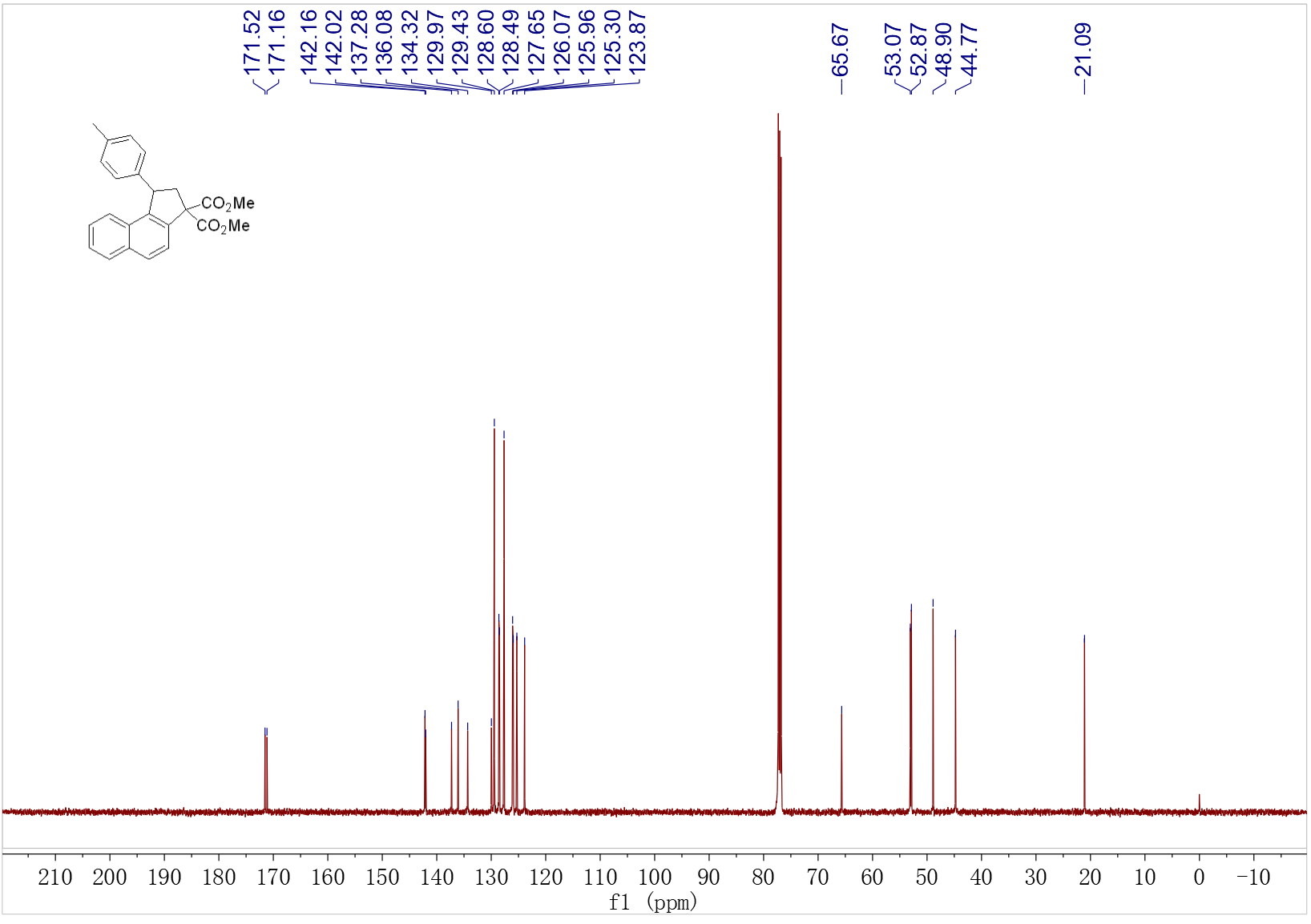


**Supplementary Figure 37. ^13^C NMR spectrum of 3t (125 MHz, CDCl_3_)**


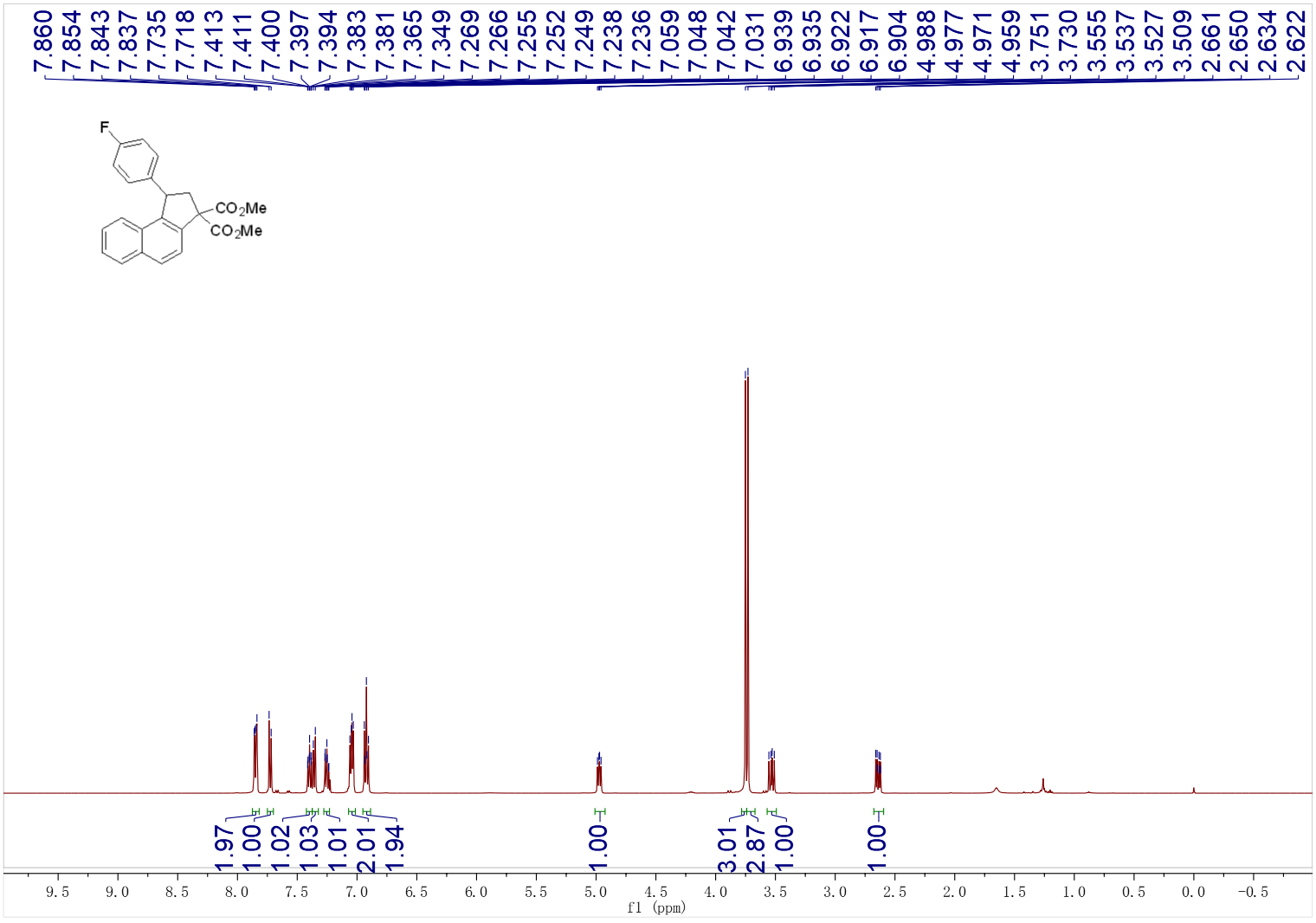


**Supplementary Figure 38. ^1^H NMR spectrum of 3u (500 MHz, CDCl_3_)**


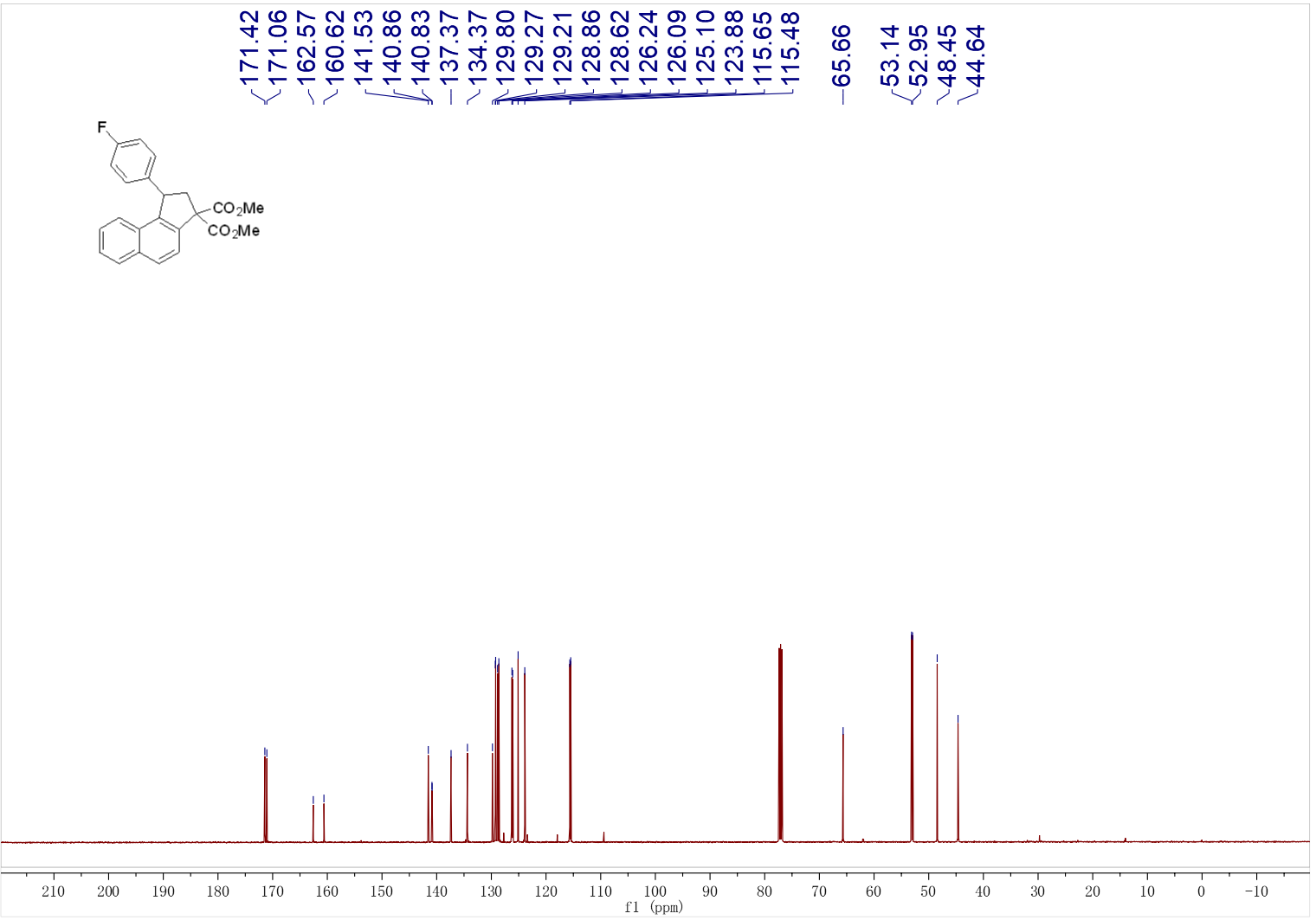


**Supplementary Figure 39. ^13^C NMR spectrum of 3u (125 MHz, CDCl_3_)**


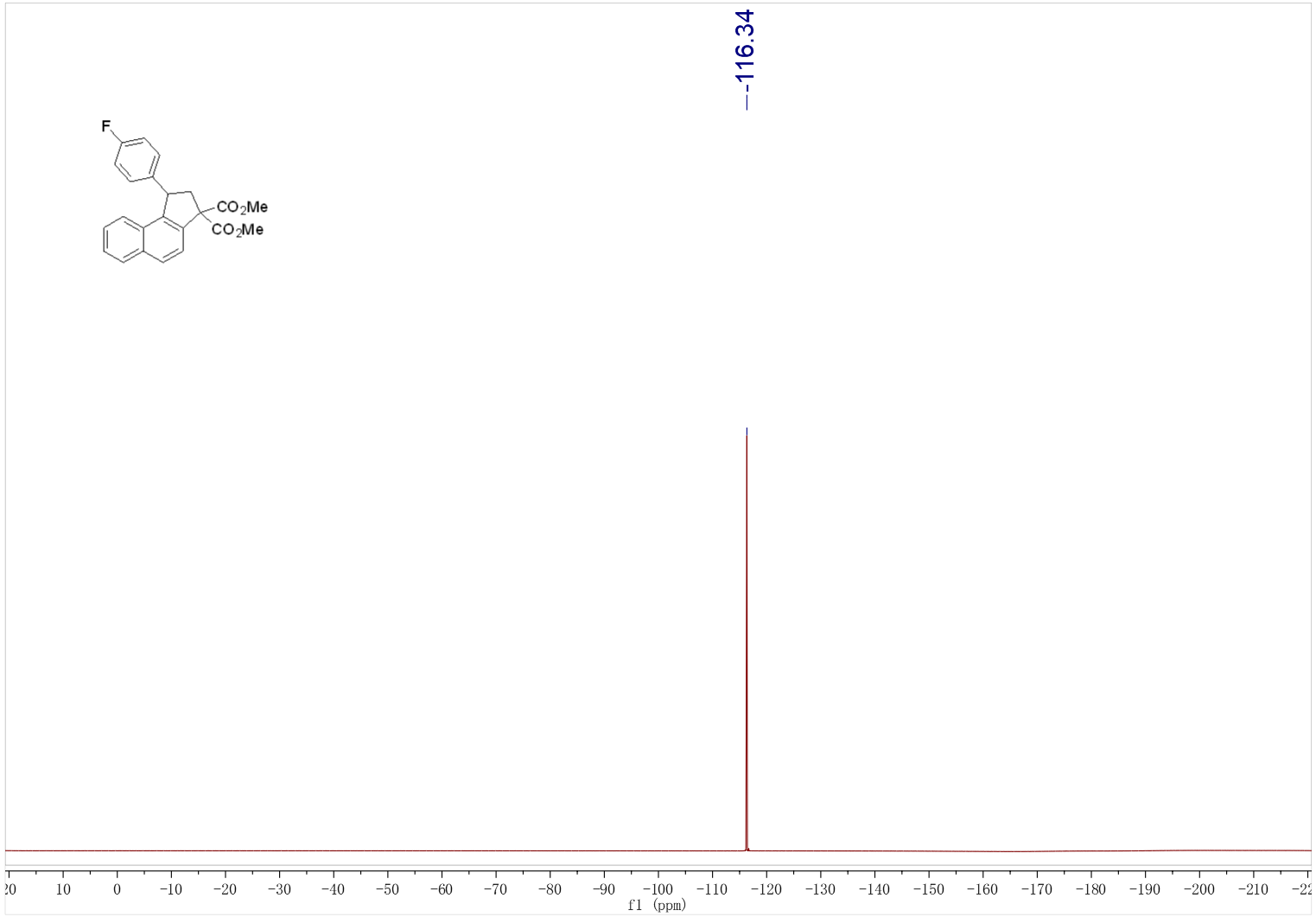


**Supplementary Figure 40. ^19^F NMR spectrum of 3u (470 MHz, CDCl_3_)**


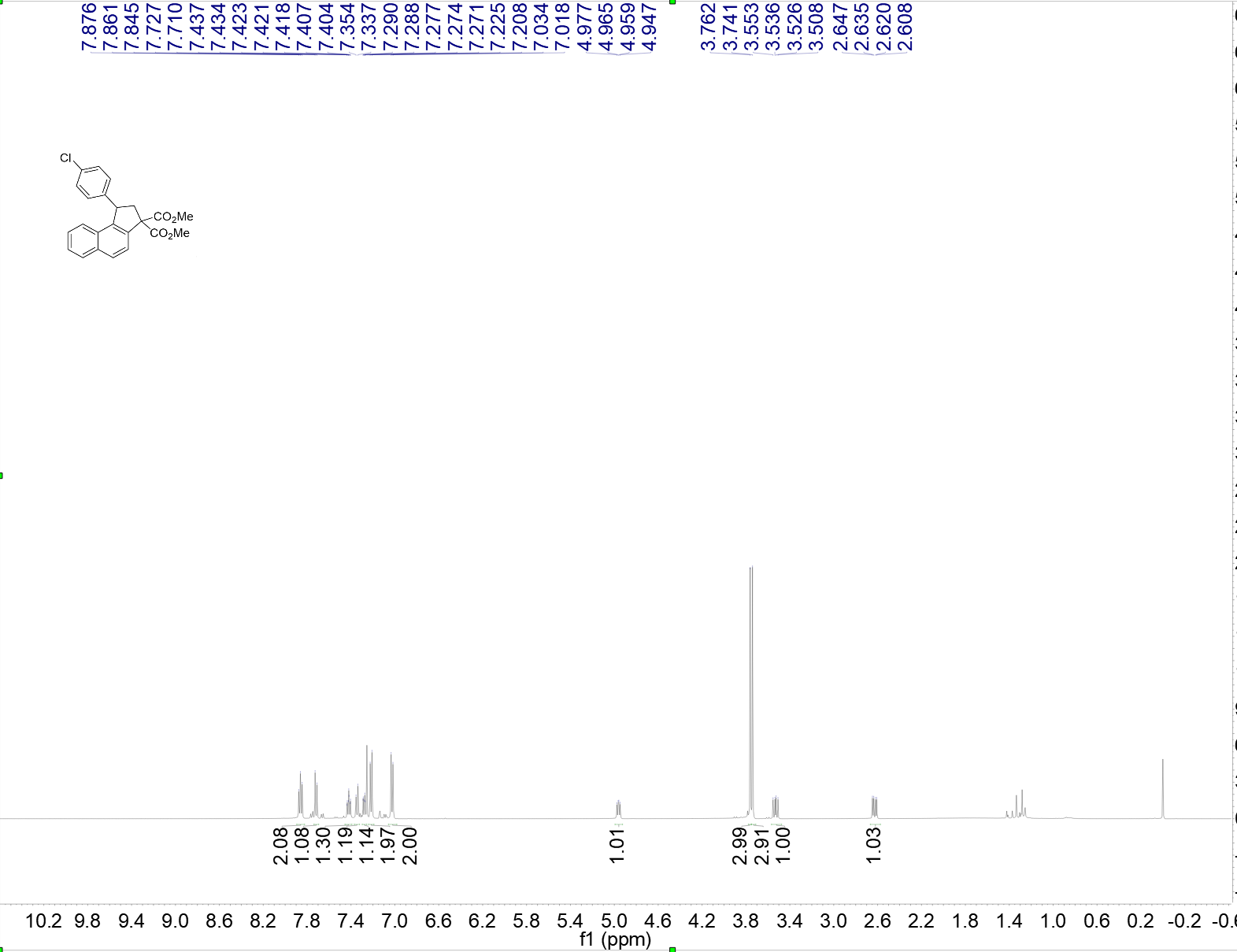


**Supplementary Figure 41. ^1^H NMR spectrum of 3v (500 MHz, CDCl_3_)**


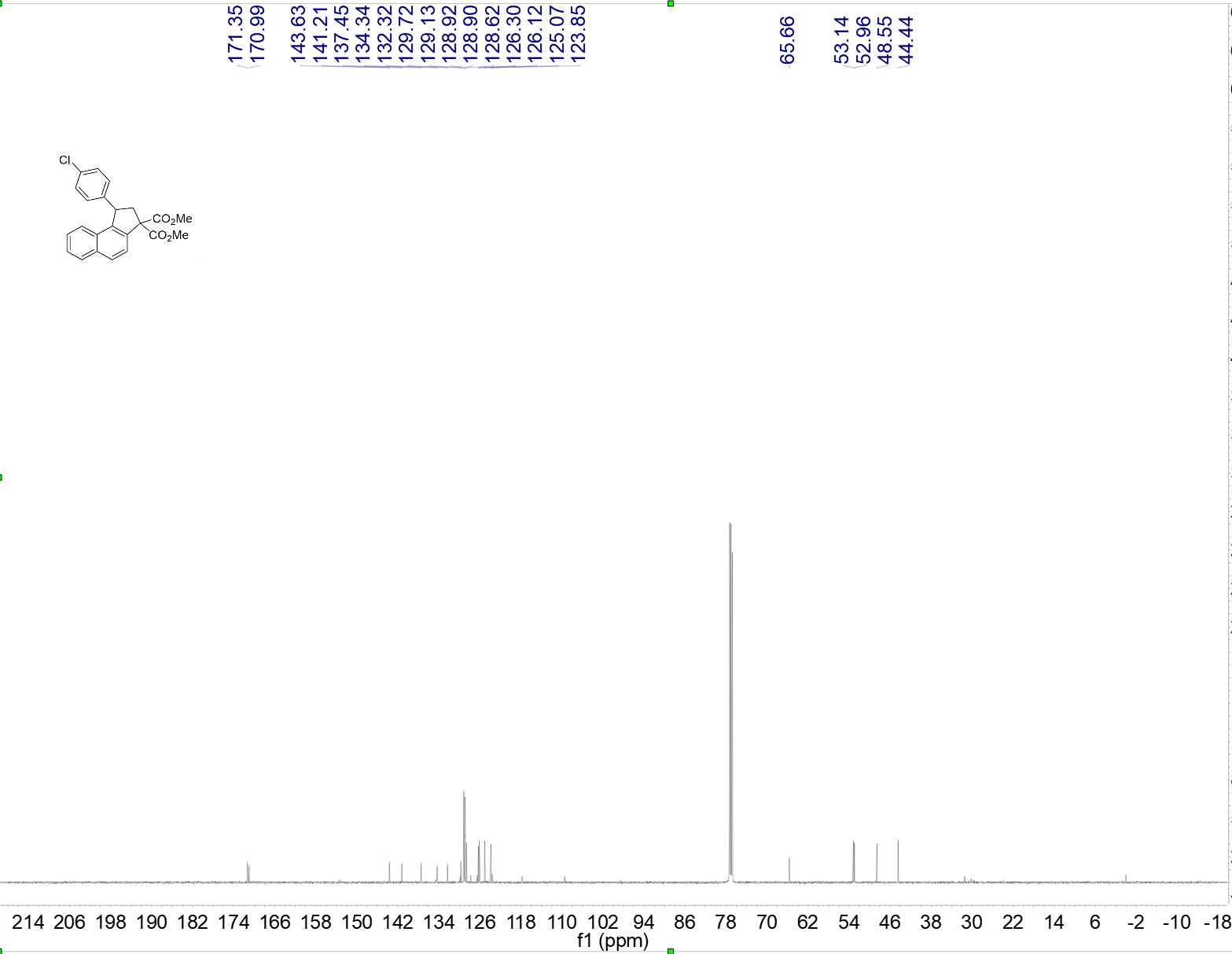


**Supplementary Figure 42. ^13^C NMR spectrum of 3v (125 MHz, CDCl_3_)**


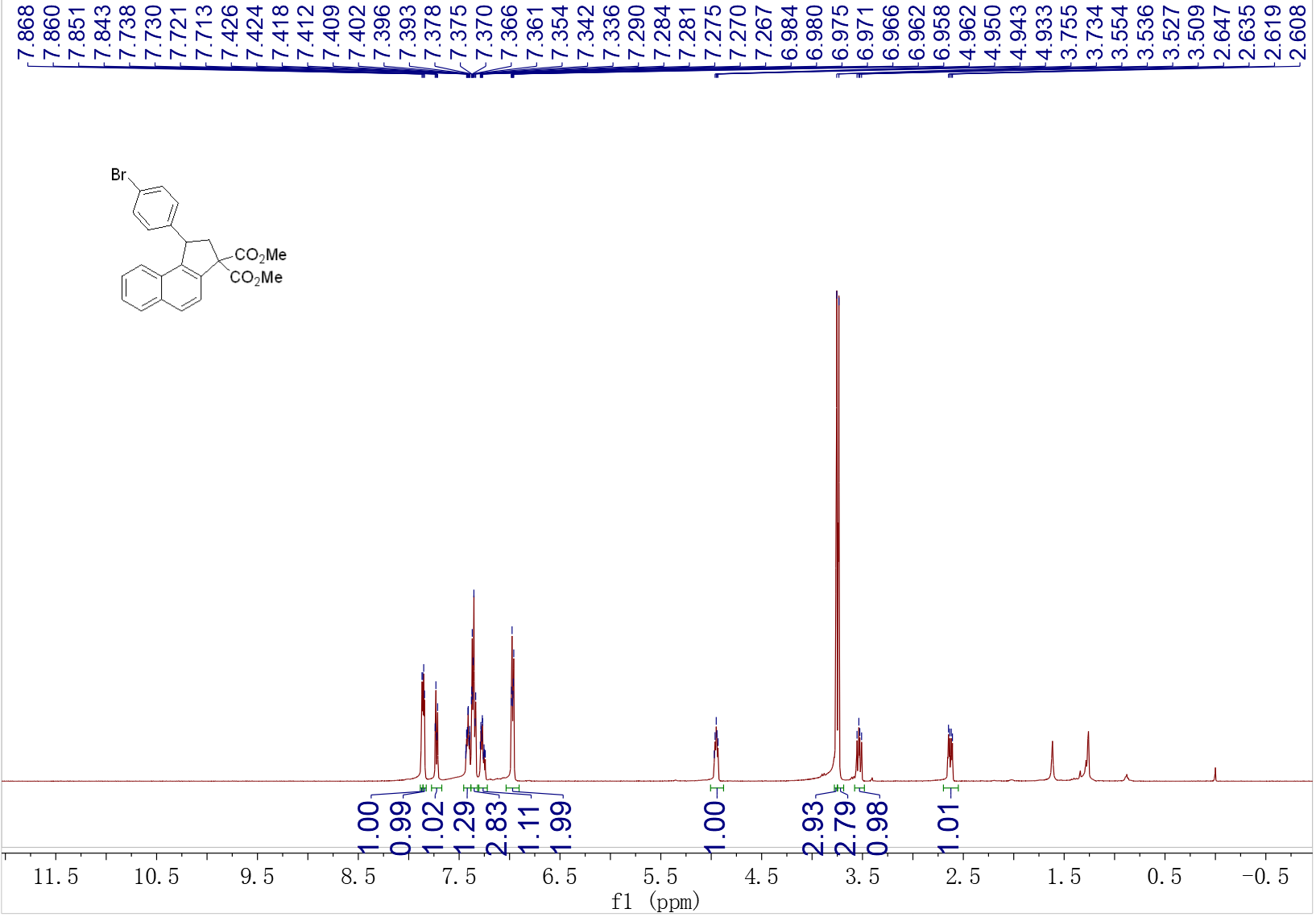


**Supplementary Figure 43. ^1^H NMR spectrum of 3w (500 MHz, CDCl_3_)**


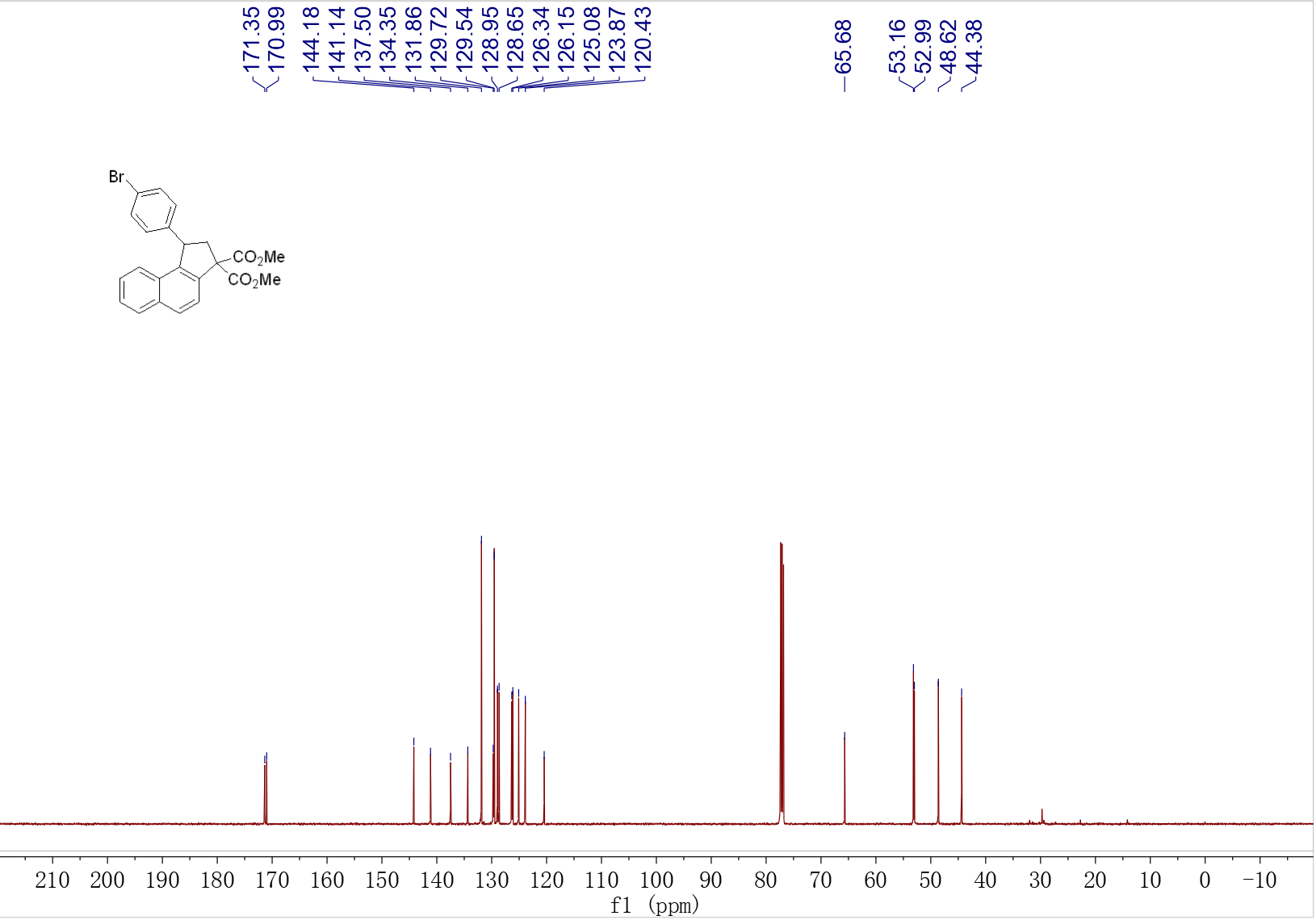


**Supplementary Figure 44. ^13^C NMR spectrum of 3w (125 MHz, CDCl_3_)**


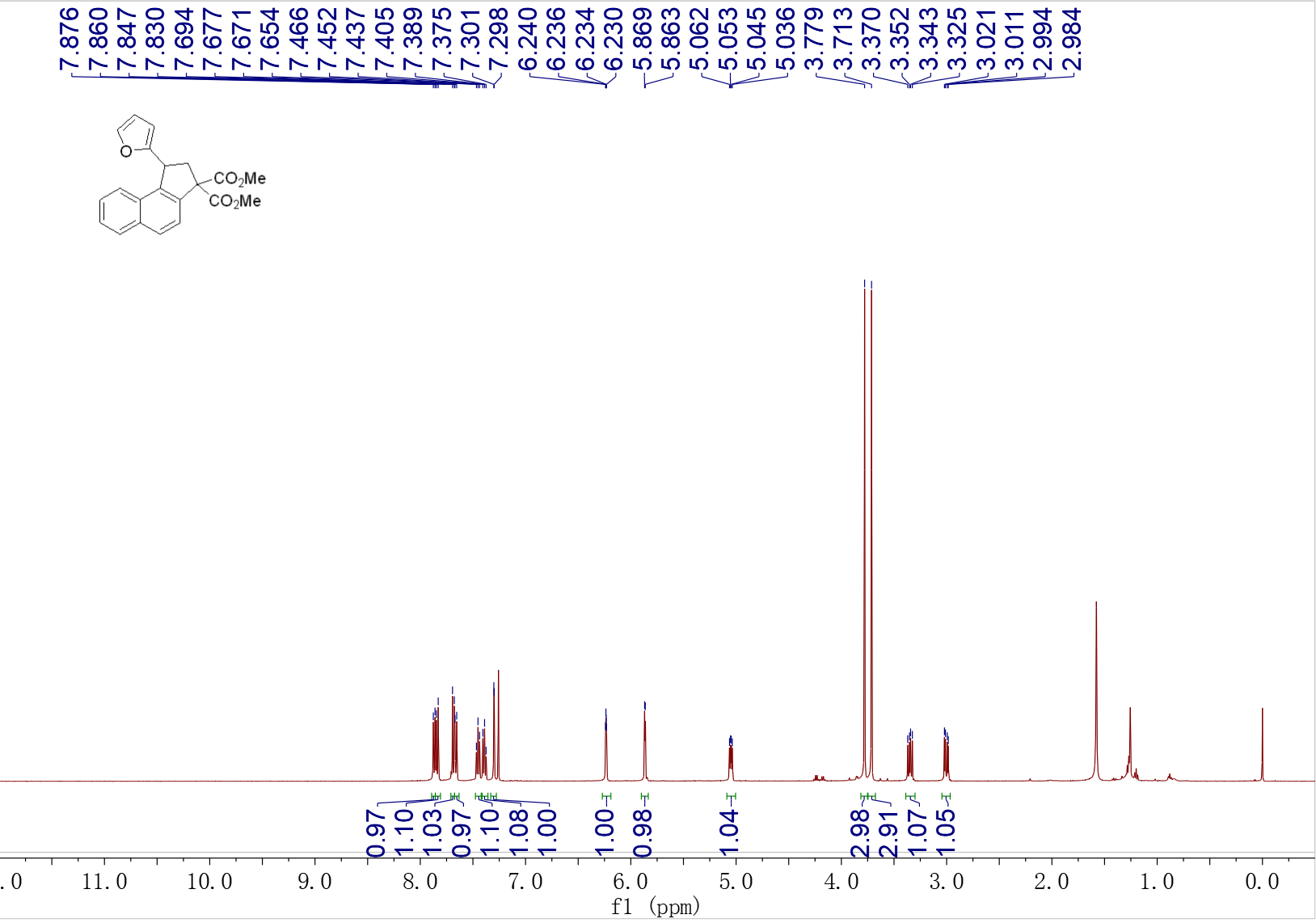


**Supplementary Figure 45. ^1^H NMR spectrum of 3x (500 MHz, CDCl_3_)**


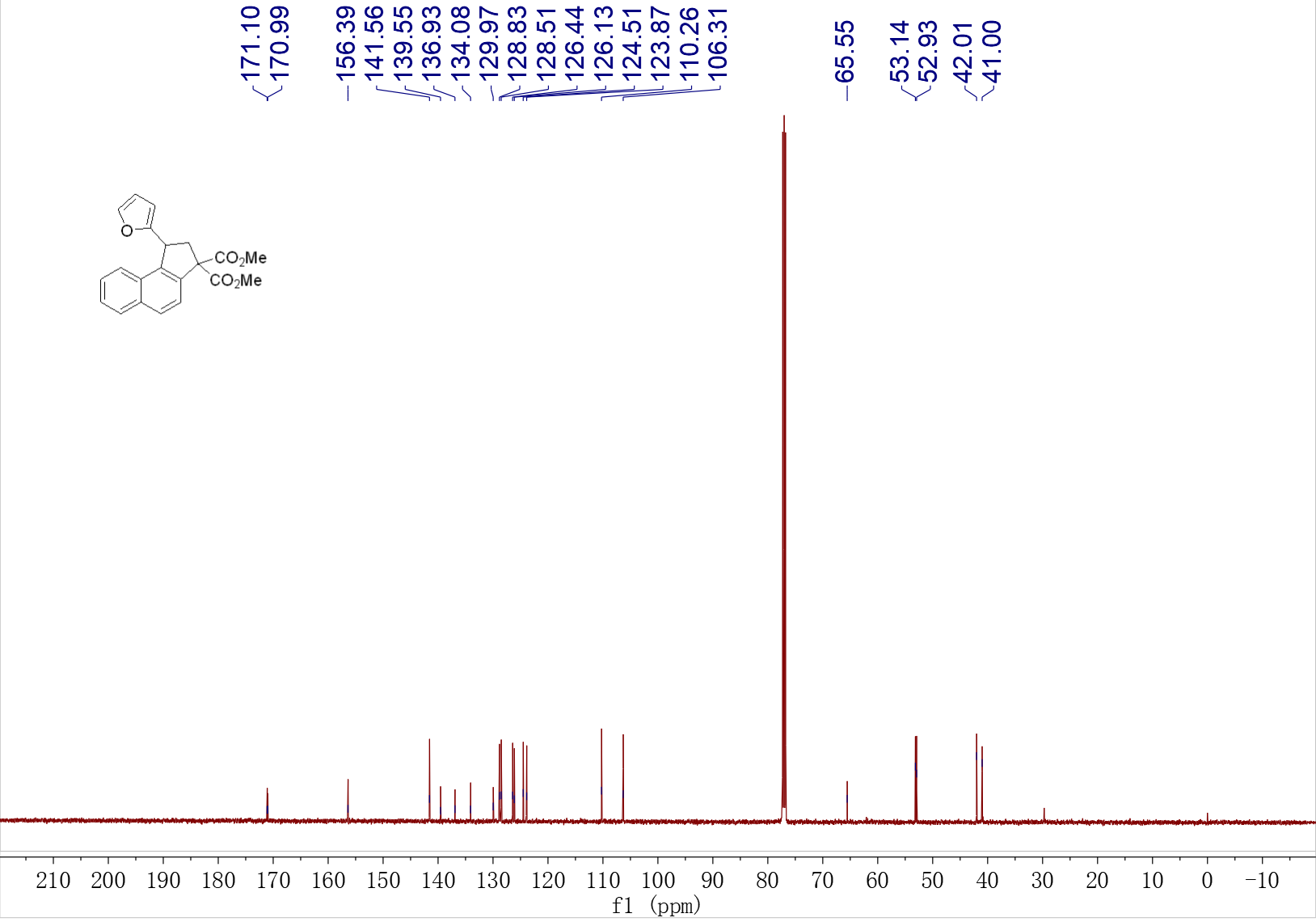


**Supplementary Figure 46. ^13^C spectrum NMR of 3x (125 MHz, CDCl_3_)**


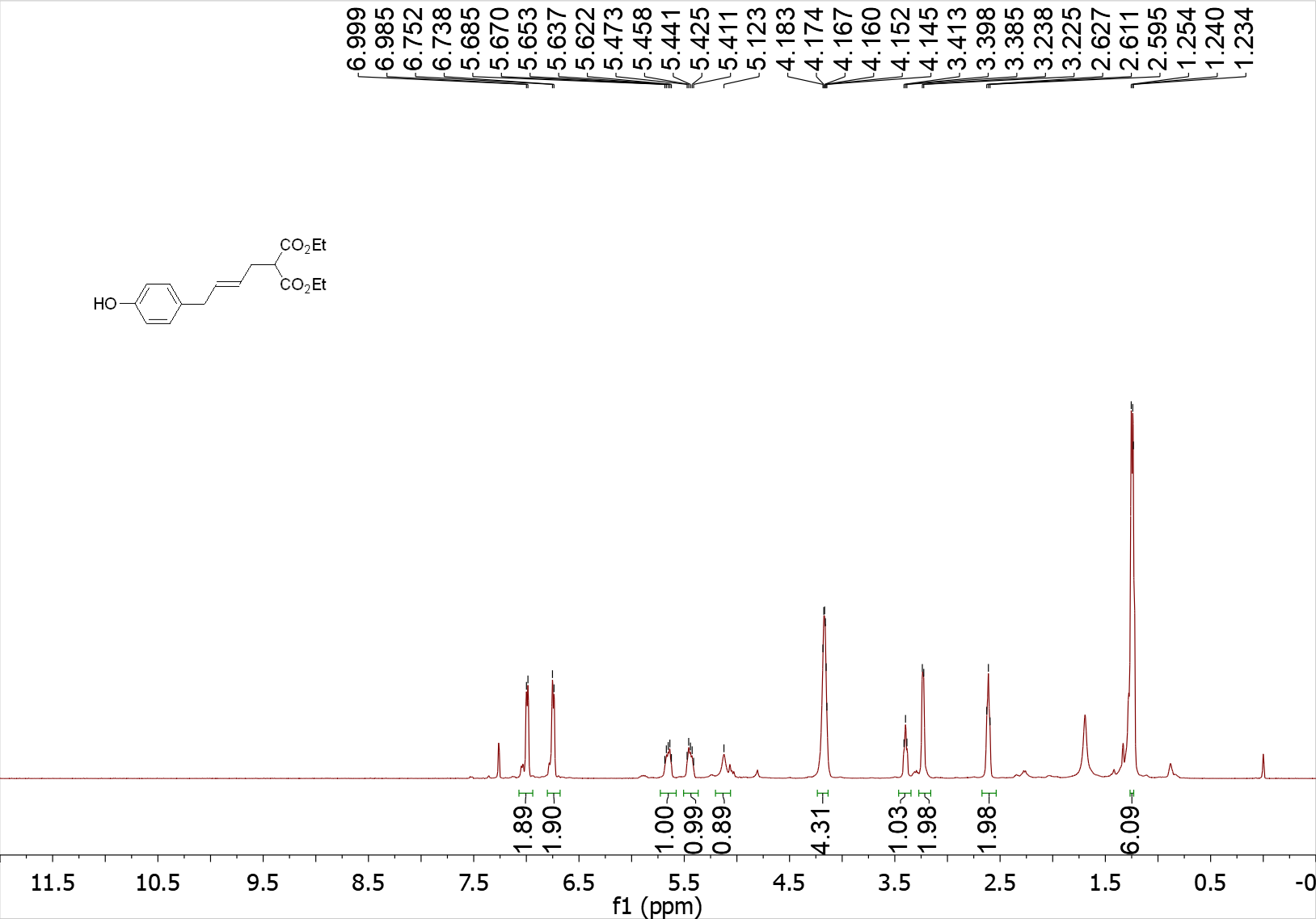


**Supplementary Figure 47. ^1^H NMR spectrum of 5 (500 MHz, CDCl_3_)**


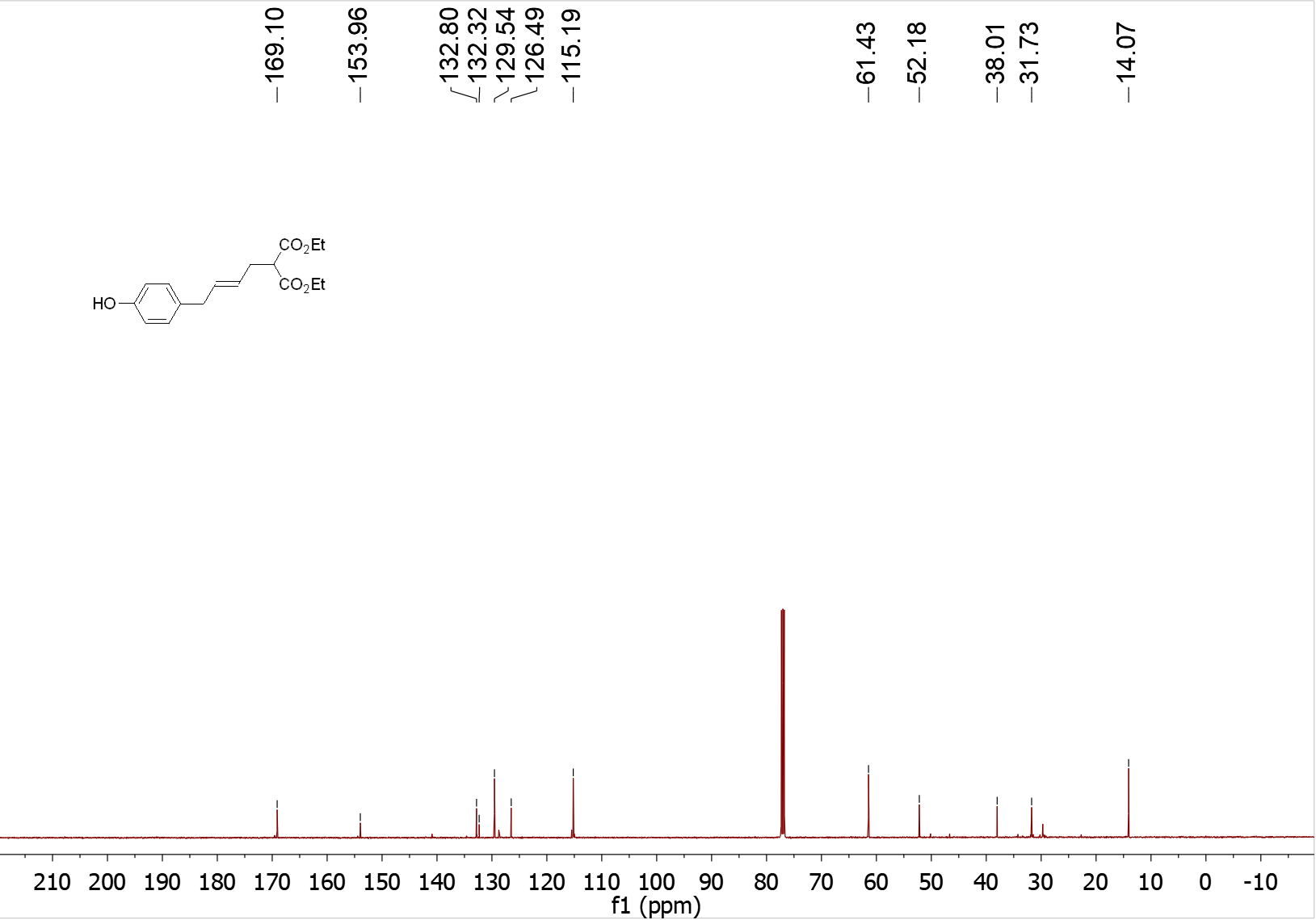


**Supplementary Figure 48. ^13^C NMR spectrum of 5 (125 MHz, CDCl_3_)**


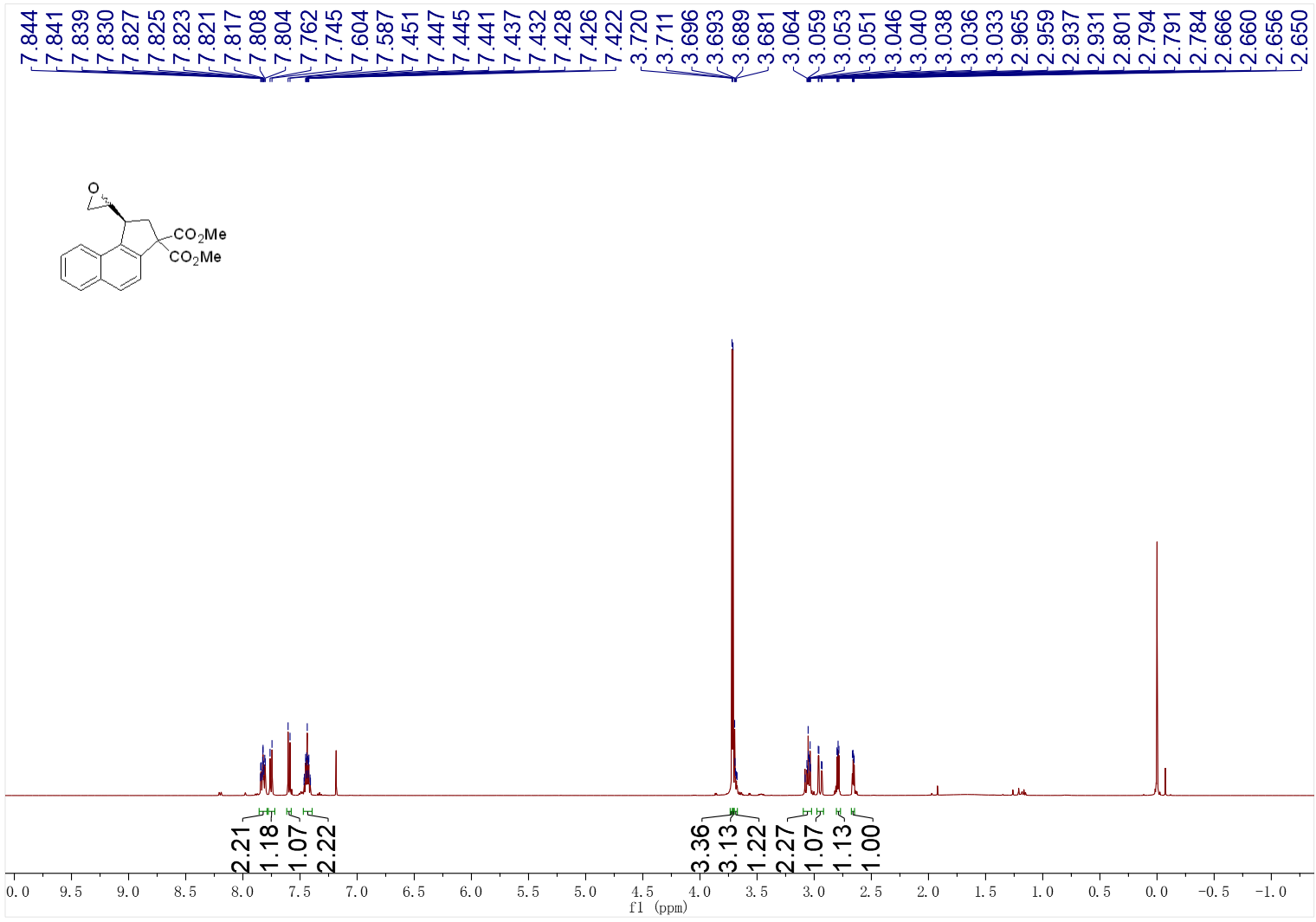


**Supplementary Figure 49. ^1^H NMR spectrum of 6a (500 MHz, CDCl_3_)**


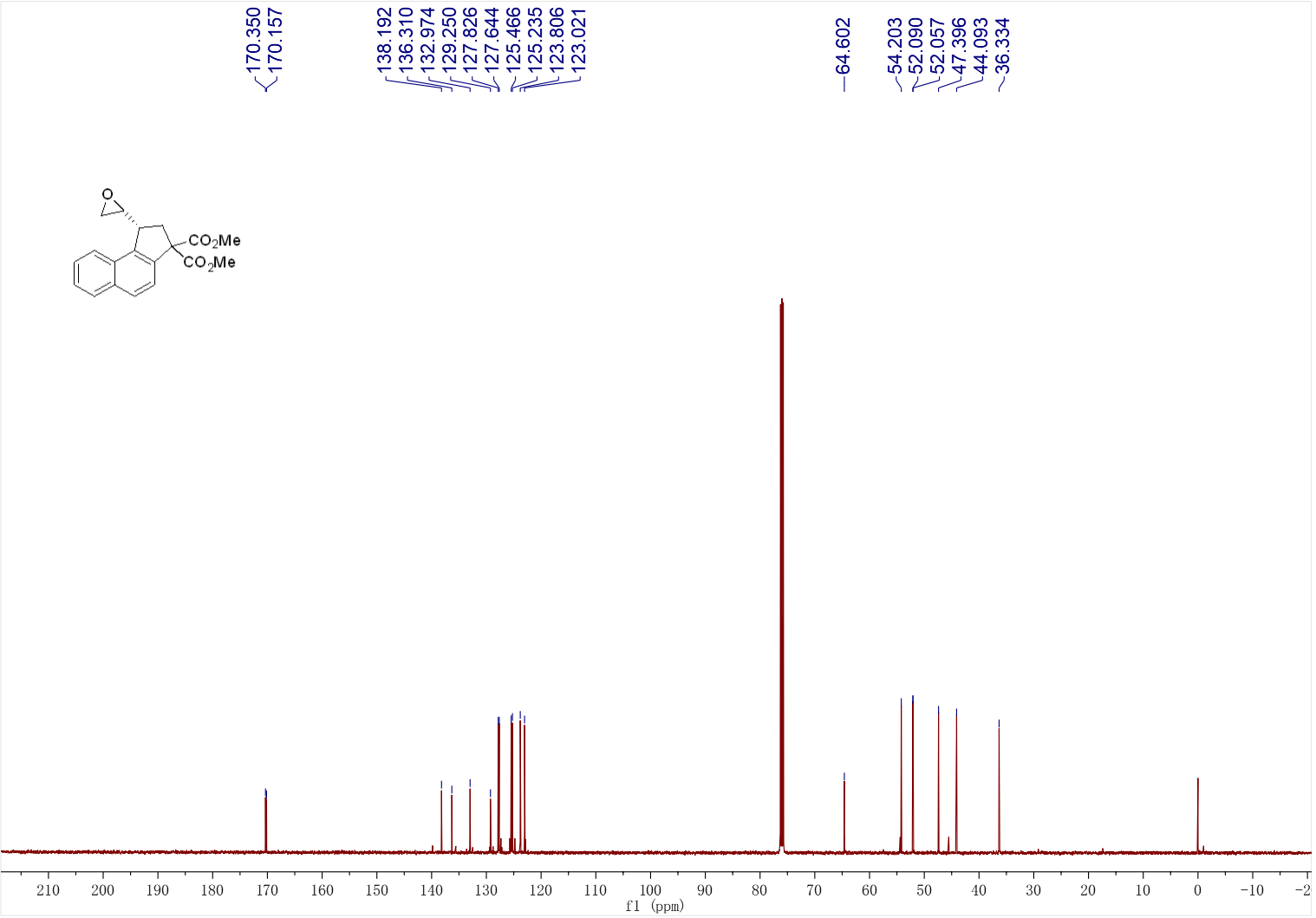


**Supplementary Figure 50. ^13^C NMR of 6a (125 MHz, CDCl_3_)**


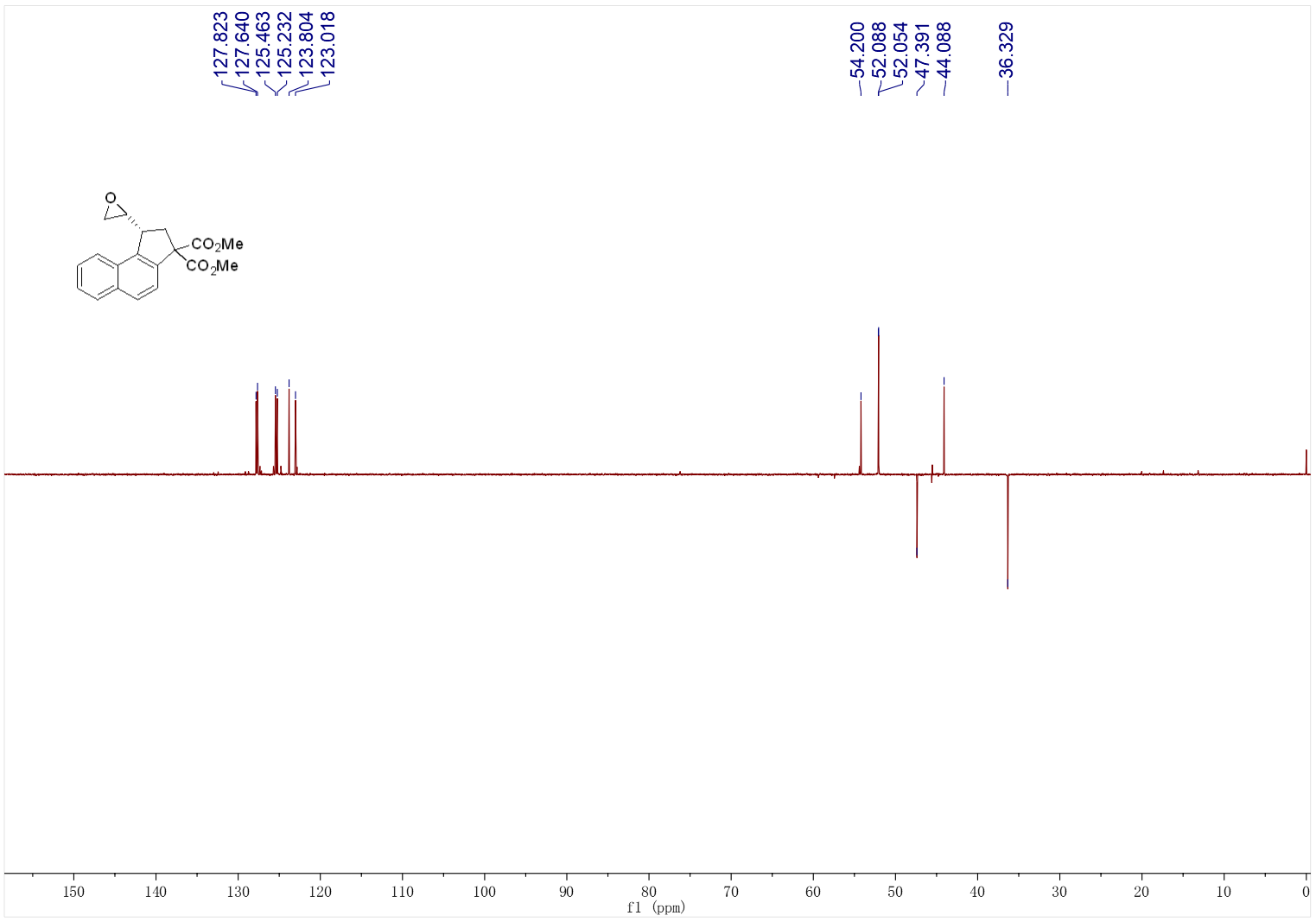


**Supplementary Figure 51. ^13^C DEPT-135 NMR of 6a (125 MHz, CDCl_3_)**


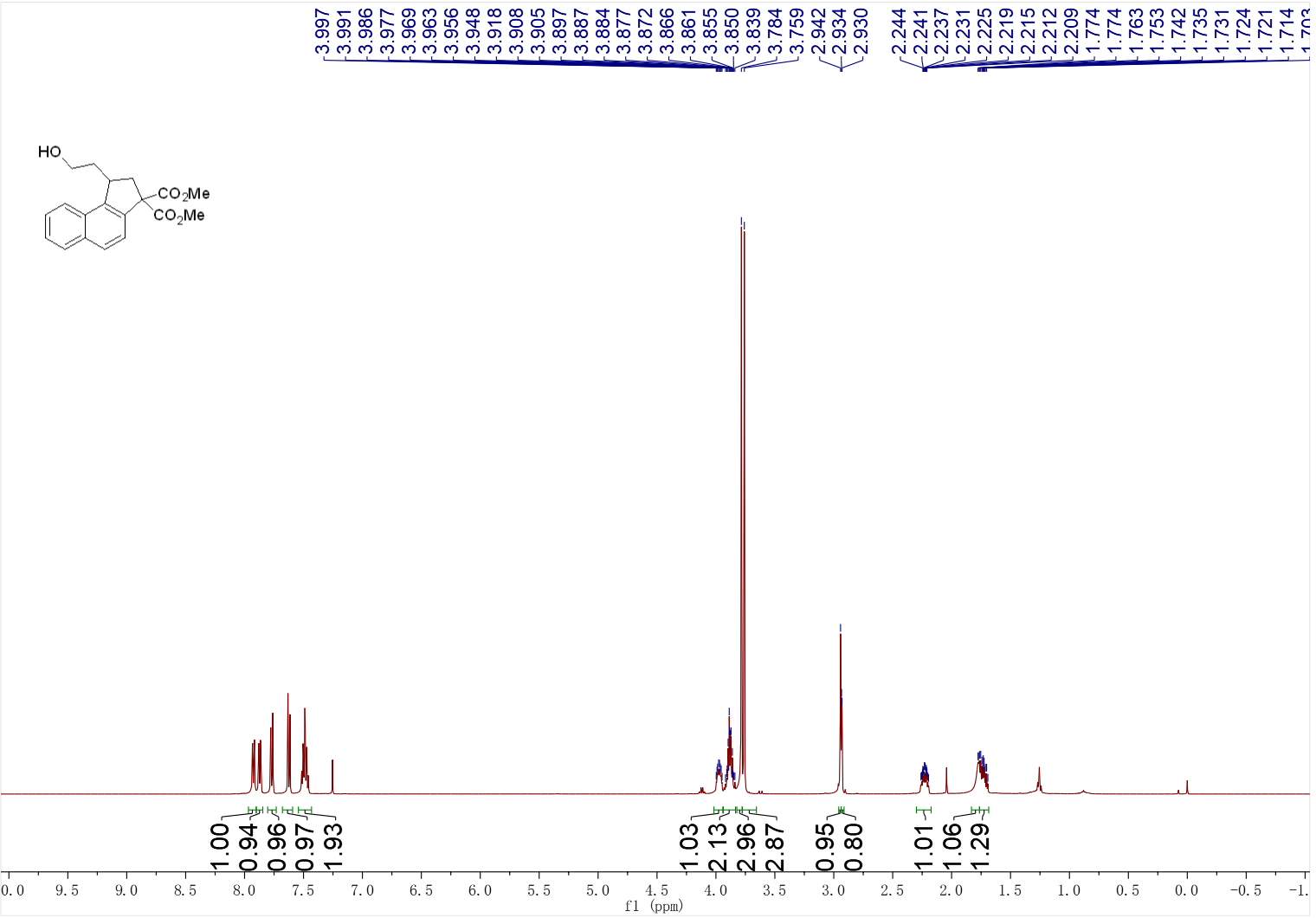


**Supplementary Figure 52. ^1^H NMR spectrum of 6b (500 MHz, CDCl_3_)**


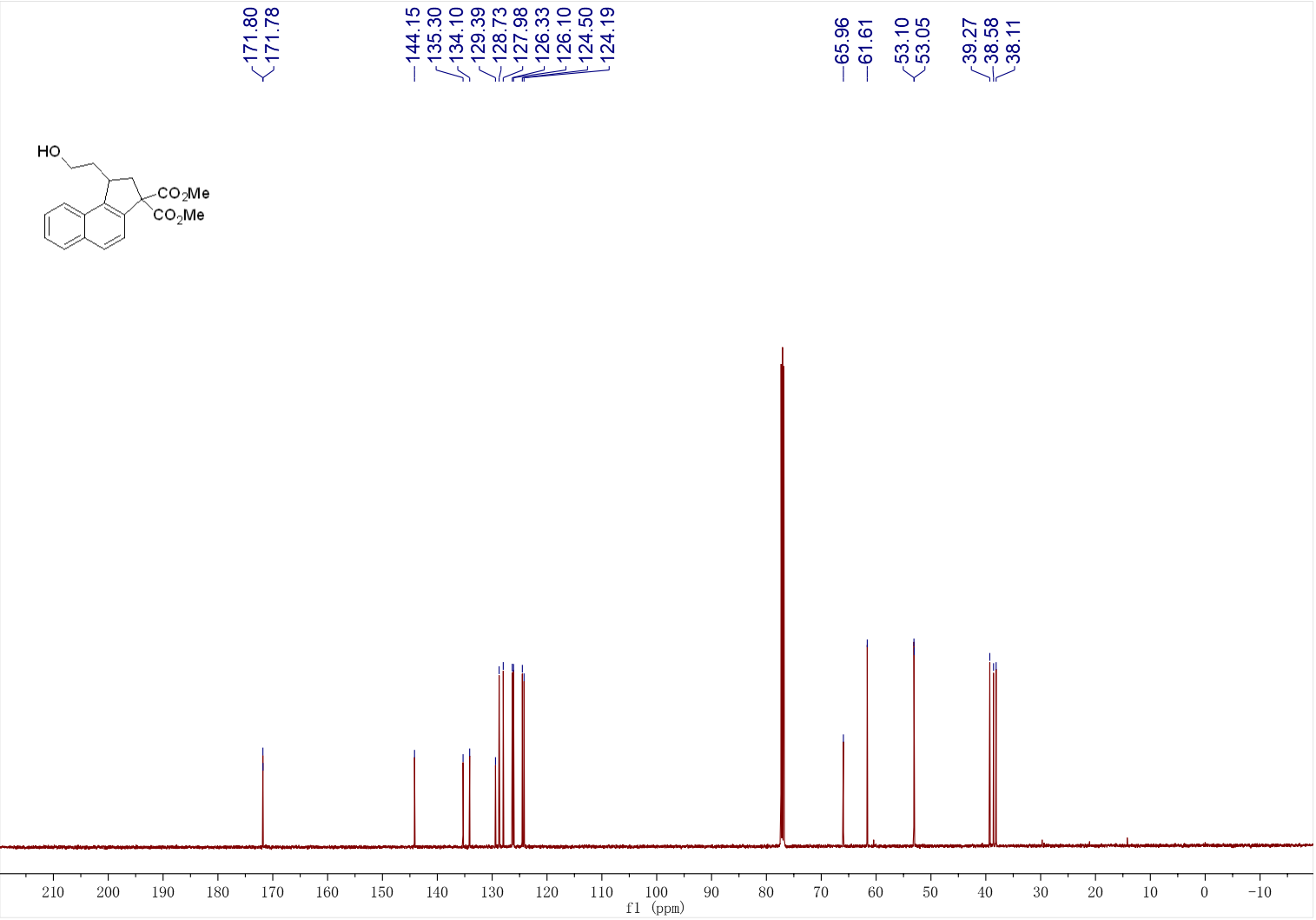


**Supplementary Figure 53. ^13^C NMR of 6b (125 MHz, CDCl_3_)**


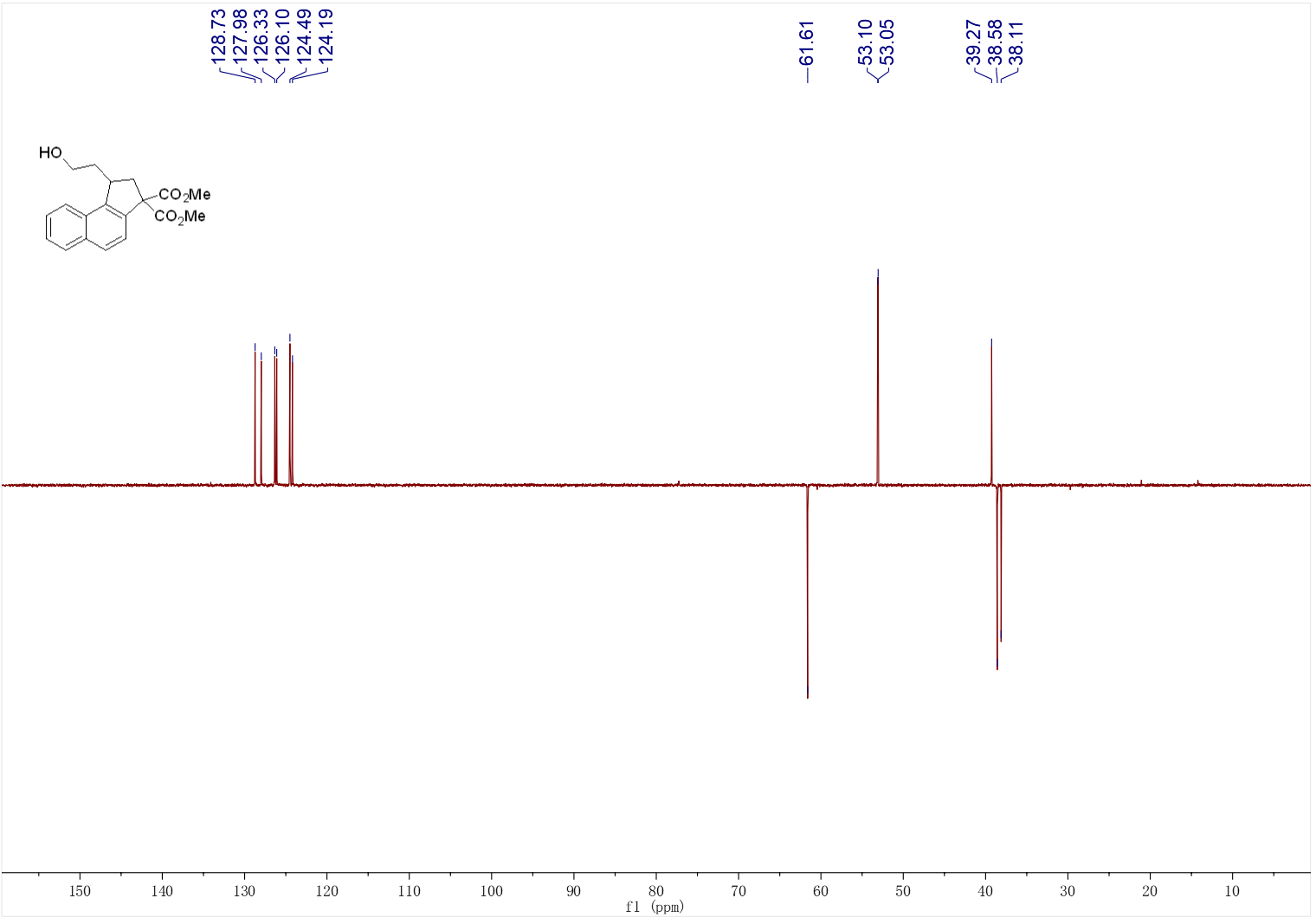


**Supplementary Figure 54. ^13^C DEPT-135 NMR of 6b (125 MHz, CDCl_3_)**


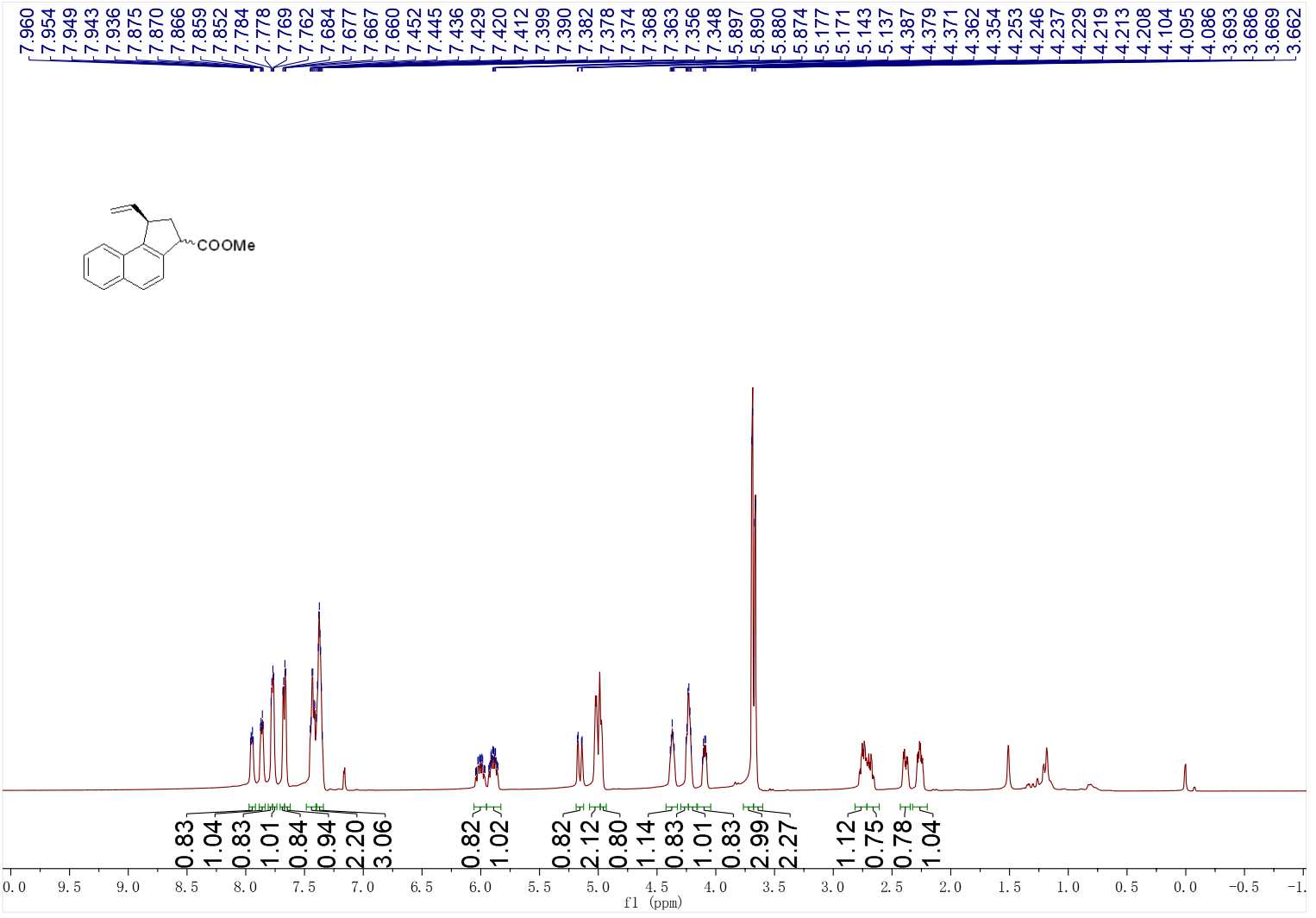


**Supplementary Figure 55. ^1^H NMR spectrum of 6c (500 MHz, CDCl_3_)**


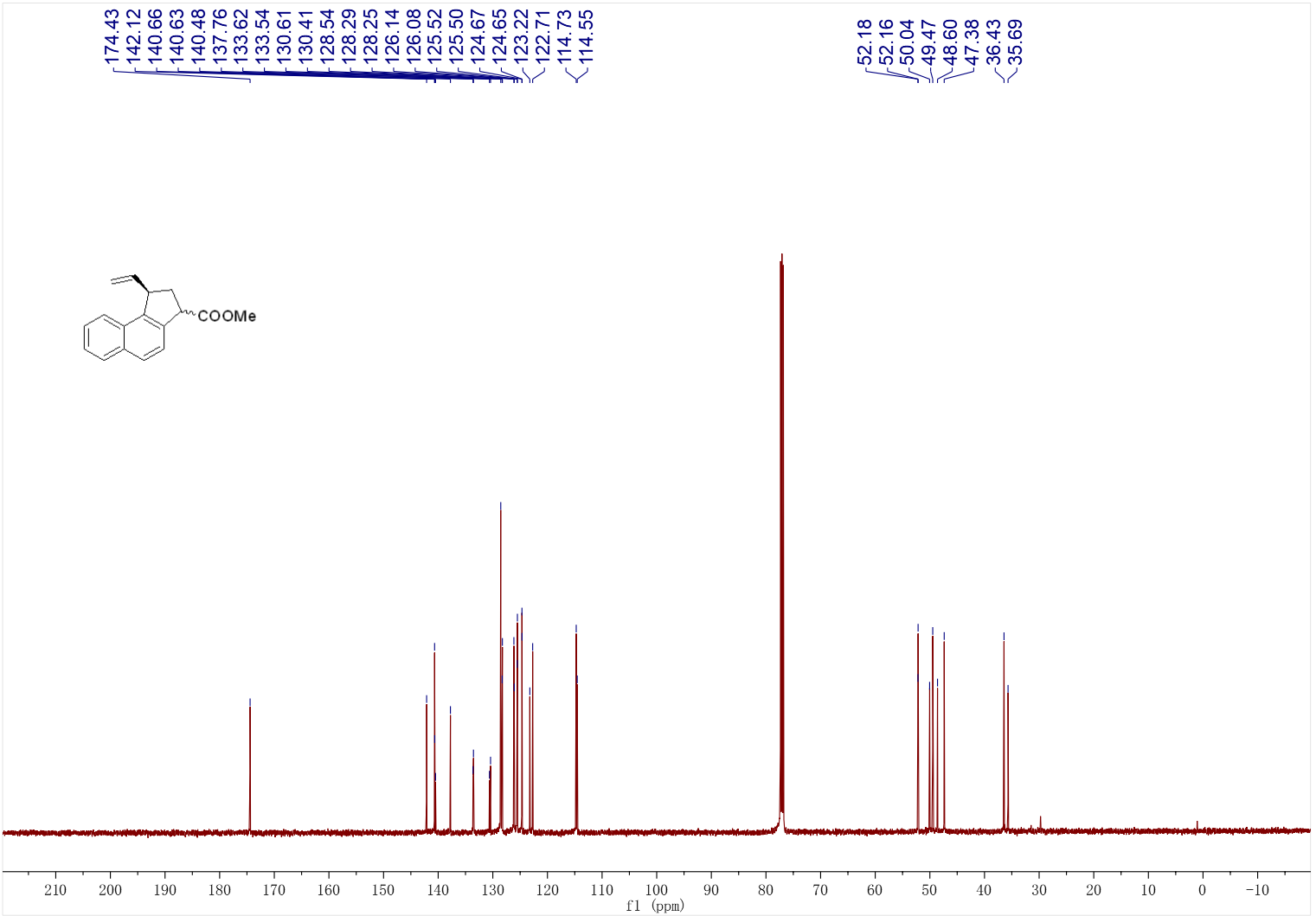


**Supplementary Figure 56. ^13^C NMR spectrum of 6c (125 MHz, CDCl_3_)**


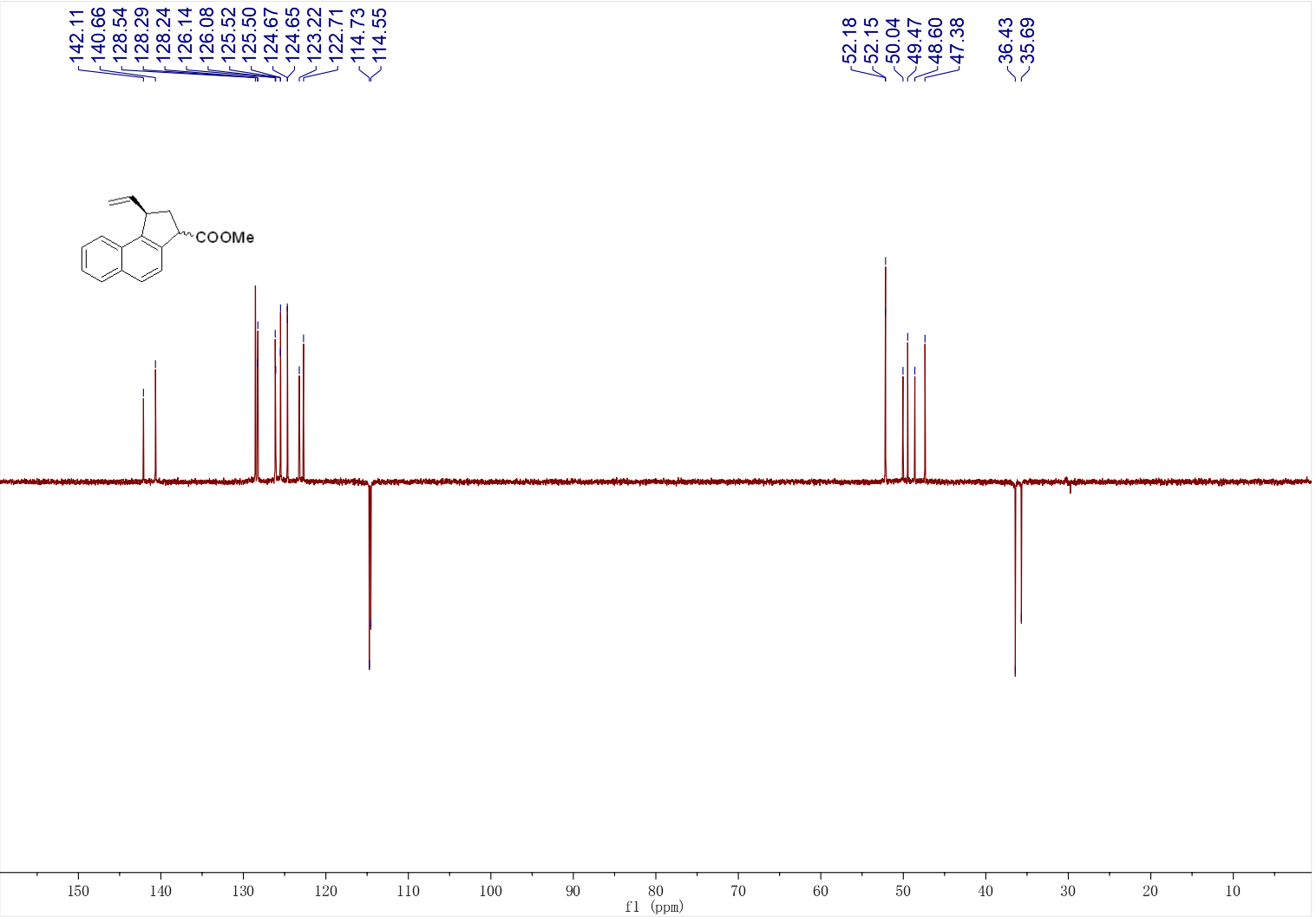


**Supplementary Figure 57. ^13^C DEPT-135 NMR of 6c (125 MHz, CDCl_3_)**


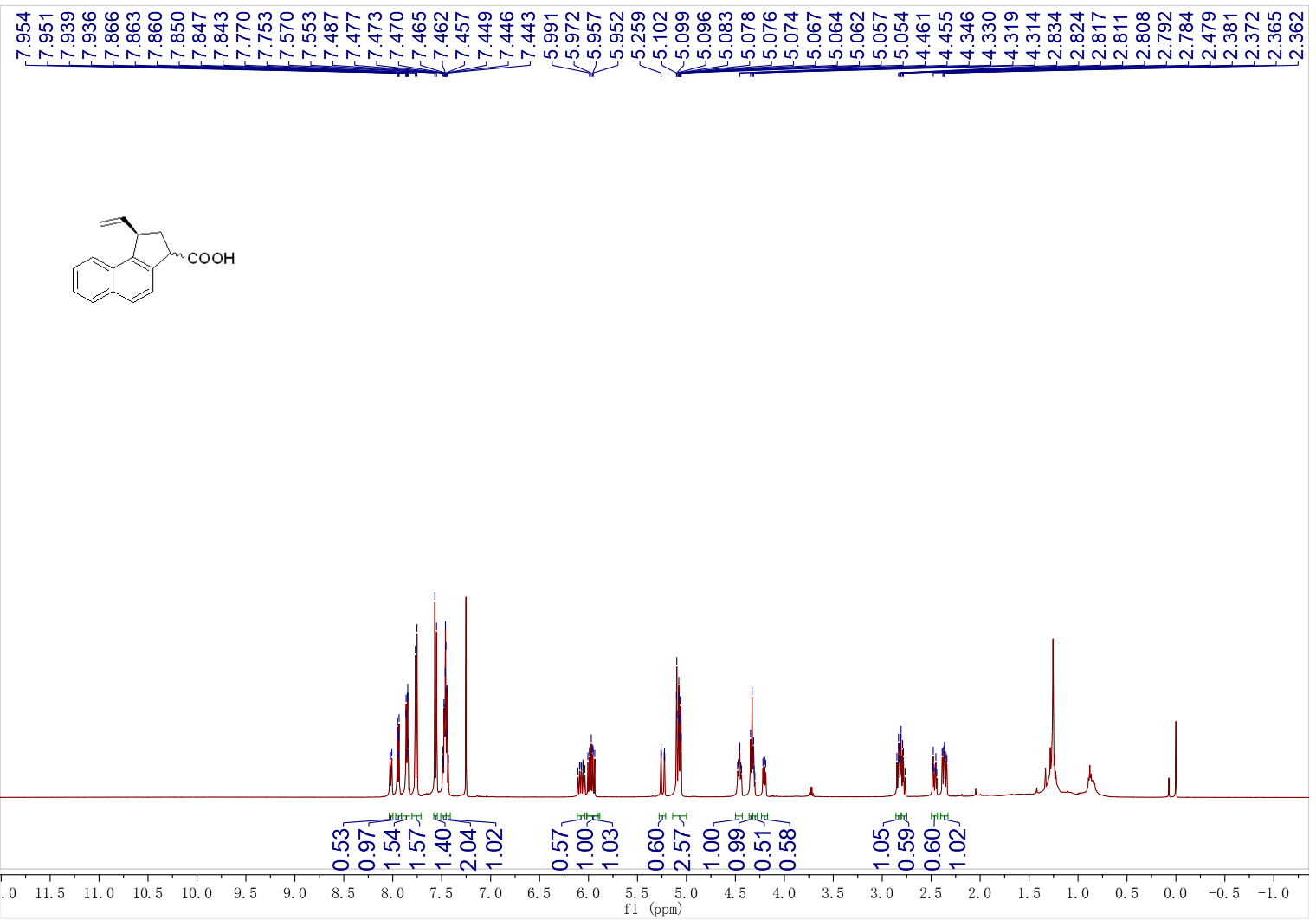


**Supplementary Figure 58. ^1^H NMR spectrum of 6d (500 MHz, CDCl_3_)**


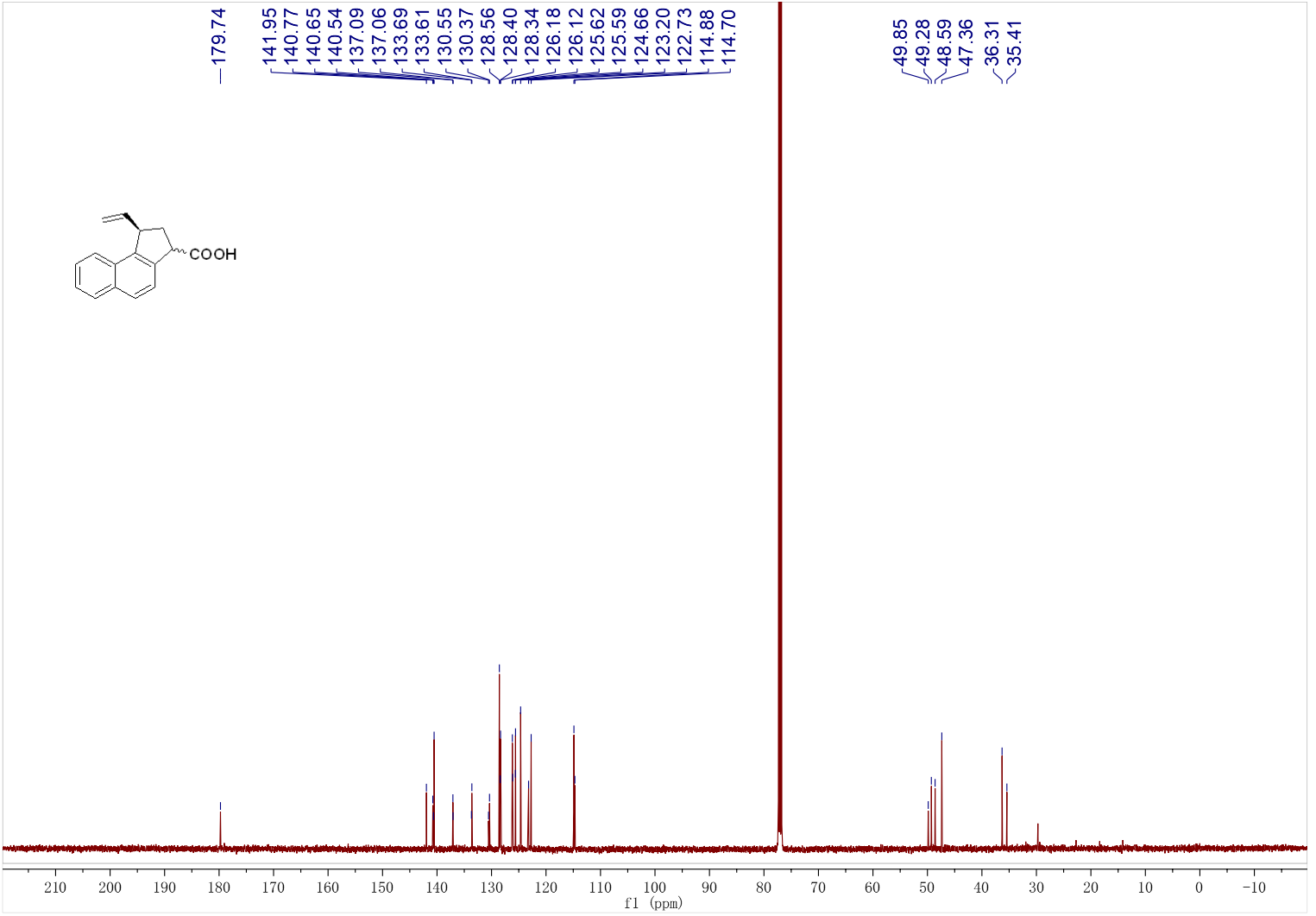


**Supplementary Figure 59. ^13^C NMR spectrum of 6d (125 MHz, CDCl_3_)**


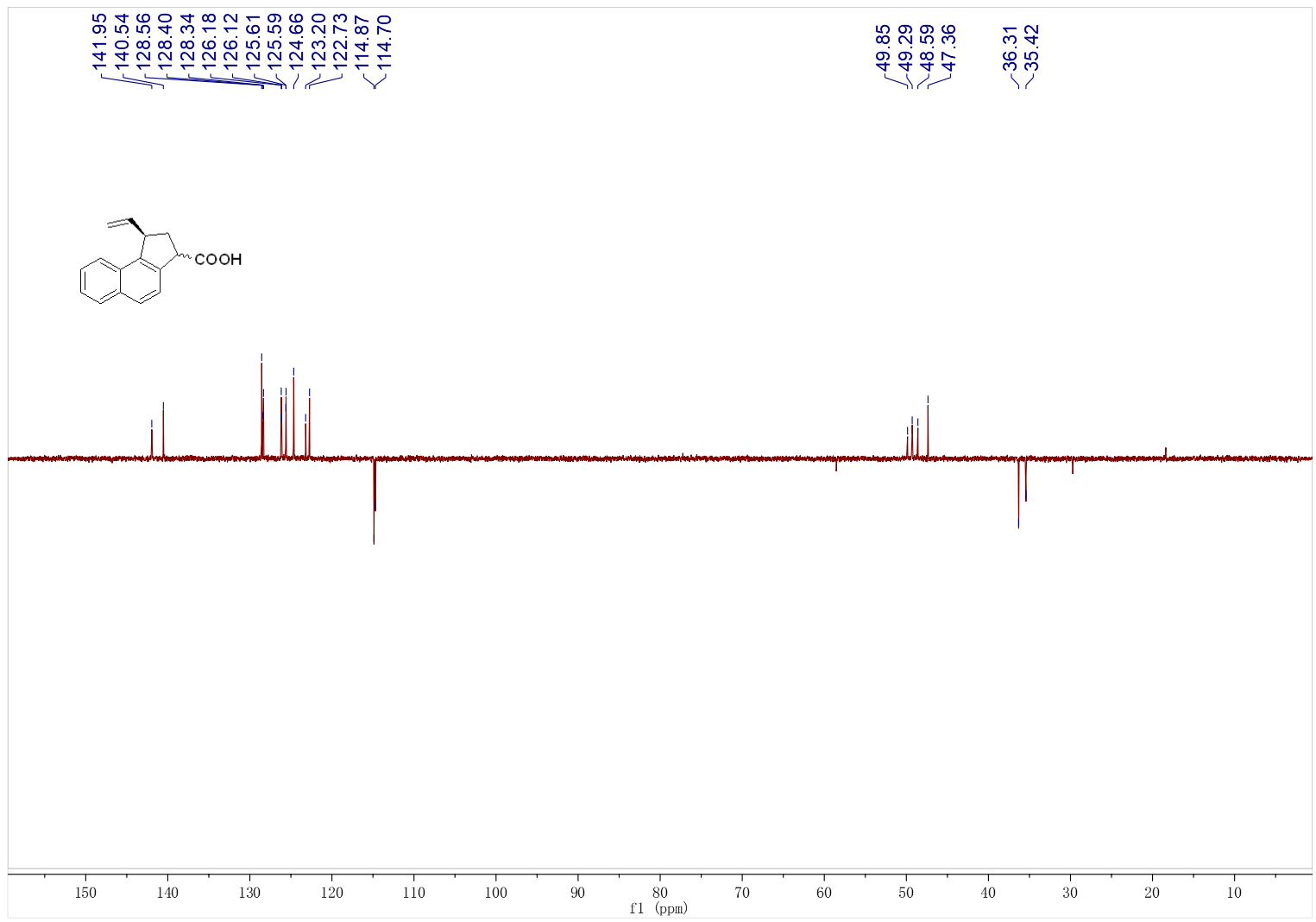


**Supplementary Figure 60. ^13^C DEPT-135 NMR of 6d (125 MHz, CDCl_3_)**
